# UHPLC-ESI-QTOF-MS/MS-Based Molecular Networking Guided Isolation and Dereplication of Antibacterial and Antifungal Constituents of *Ventilago denticulata*

**DOI:** 10.3390/antibiotics9090606

**Published:** 2020-09-15

**Authors:** Muhaiminatul Azizah, Patcharee Pripdeevech, Tawatchai Thongkongkaew, Chulabhorn Mahidol, Somsak Ruchirawat, Prasat Kittakoop

**Affiliations:** 1 Chulabhorn Graduate Institute, Chemical Biology Program, Chulabhorn Royal Academy, Laksi, Bangkok 10210, Thailand; mimi.hufflepuf@gmail.com (M.A.); tawatchait@cgi.ac.th (T.T.); mahidol_natlab@cri.or.th (C.M.); somsak@cri.or.th (S.R.); 2 School of Science, Mae Fah Luang University, Muang, Chiang Rai 57100, Thailand; patcharee.pri@mfu.ac.th; 3 Center of Chemical Innovation for Sustainability (CIS), Mae Fah Luang University, Muang, Chiang Rai 57100, Thailand; 4 Chulabhorn Research Institute, Kamphaeng Phet 6 Road, Laksi, Bangkok 10210, Thailand; 5 Center of Excellence on Environmental Health and Toxicology (EHT), CHE, Ministry of Education, Bangkok 10210, Thailand

**Keywords:** *Ventilago denticulata*, natural products, antibacterial activity, antifungal activity, dereplication, molecular networking, flavonoid glycosides, mass spectrometry, MS fragmentation of sugar

## Abstract

*Ventilago denticulata* is an herbal medicine for the treatment of wound infection; therefore this plant may rich in antibacterial agents. UHPLC-ESI-QTOF-MS/MS-Based molecular networking guided isolation and dereplication led to the identification of antibacterial and antifungal agents in *V. denticulata*. Nine antimicrobial agents in *V. denticulata* were isolated and characterized; they are divided into four groups including (I) flavonoid glycosides, rhamnazin 3-rhamninoside (**7**), catharticin or rhamnocitrin 3-rhamninoside (**8**), xanthorhamnin B or rhamnetin 3-rhamninoside (**9**), kaempferol 3-rhamninoside (**10**) and flavovilloside or quercetin 3-rhamninoside (**11**), (II) benzisochromanquinone, ventilatones B (**12**) and A (**15**), (III) a naphthopyrone ventilatone C (**16**) and (IV) a triterpene lupeol (**13**). Among the isolated compounds, ventilatone C (**16**) was a new compound. Moreover, kaempferol, chrysoeriol, isopimpinellin, rhamnetin, luteolin, emodin, rhamnocitrin, ventilagodenin A, rhamnazin and mukurozidiol, were tentatively identified as antimicrobial compounds in extracts of *V. denticulata* by a dereplication method. MS fragmentation of rhamnose-containing compounds gave an oxonium ion, C_6_H_9_O_3_^+^ at *m/z* 129, while that of galactose-containing glycosides provided the fragment ion at *m/z* 163 of C_6_H_11_O_5_^+^. These fragment ions may be used to confirm the presence of rhamnose or galactose in mass spectrometry-based analysis of natural glycosides or oligosaccharide attached to biomolecules, that is, glycoproteins.

## 1. Introduction

Natural products are important sources of drugs and they provide many building blocks for drug discovery [1]. Statistically, around 50% of approved drugs were derived from natural products [2]. From 1931 to 2013, new chemical entities from natural products approved by the US Food and Drug Administration (FDA), are approximately 47% derived from plants, followed by 30% from bacteria, 23% from fungi and 5% from other natural sources [3]. As per the World Health Organisation (WHO), approximately 65% of the population of the world particularly in developing countries, mostly rely on utilization of plant-derived traditional medicines for health care and ethnomedical-based treatments [4]. Furthermore, in 2015, Youyou Tu received the Nobel Prize award for the discovery of artemisinin as an anti-malarial drug from the plant *Artemisia annua*; this underscores the importance of plant metabolites as sources of modern drugs [2]. According to these data, plants are rich sources of bioactive compounds, contributing significantly to drug discovery.

A conventional approach for drug discovery from natural products takes long time and high cost with hard efforts in purification, isolation and identification of natural products [5]. Moreover, the end of this process may result in the rediscovery of known bioactive compounds [6,7]. To increase the rate of the discovery of new natural products, dereplication technique is an alternative approach. Dereplication enables the identification of known compounds and the potential unknown compounds in crude extracts at the early stage of research before the isolation process [8]. The dereplication technique employs liquid chromatography-mass spectrometry(LC-MS), liquid chromatography-photodiode array detector (LC-PDA), liquid chromatography-nuclear magnetic resonance (LC-NMR) or other spectroscopic techniques [5,6] and LC-MS provides high sensitivity and effectiveness for the identification of natural products [6]. 

There is a limitation for LC-MS based dereplication using parent masses because it yields several molecular formulas when searching in databases [9]; this leads to less effectiveness for compound identification. Since compounds with similar structures tend to have similar MS/MS fragmentation patterns, information from MS/MS data of chemical similarity is used for molecular networking, which is considered as an effective dereplication strategy [6]. MS/MS-based molecular networking emerges as a new technique to supplement the dereplication strategy [10]. The Global Natural Products Social Molecular Networking (GNPS) website (http://gnps.ucsd.edu) is an open-access web-based mass spectrometry, facilitating high-throughput online dereplication and molecular networking analysis [11]. At present, molecular networking has been successfully employed to discover new bioactive compounds from natural sources such as the discovery of penicanesones A-C from *Penicillium canescens* and selaginpulvilins M-T from *Selaginella tamariscina* [12,13]. MS/MS-based molecular networking is involved in an untargeted fragmentation study of all compounds in crude extracts, the MS/MS spectra alignment and assembling the spectra into nodes in the network based on spectral similarity [10]. The result from MS/MS-based molecular networking is the relational networks, which reveal relationship and distribution of each chemical constituent presented in crude extracts [12,13].

Antibiotic resistance has been a public health problem worldwide. By 2050, it is predicted that death because of infection of antibiotic-resistant strains will reach approximately 10 million people per year [14]. Hence, the research on the discovery of novel antibiotics is needed. *Ventilago denticulata* Willd. is a plant in the family Rhamnaceae; previously it was named *Ventilago calyculata*. In Thailand, *V. denticulata* is called “Thao-Wan-Lek” or “Rhang-Dang.” Interestingly, in the West Midnapore district of West Bengal, the Eastern State of India, the plant *V. denticulata* is widely used to treat wound infection, suggesting the presence of antibacterial agents in this plant [15]. Bacterial strains found in wound infection were 37*%* of *Staphylococcus aureus*, 17*%* of *Pseudomonas aeruginosa* and 6*%* of *Escherichia coli* [16]. Bacteria, *Bacillus cereus* and *Salmonella enterica* serovar Typhimurium, were also found in wound infection from immunocompetent patients or diabetes mellitus patients [17,18]. *Candida albicans* was the most widely detected fungus in wound infection especially in diabetic foot ulcers [19]. Therefore, this research aims to explore antibacterial and antifungal agents in *V. denticulata*. Previously, a crude bark extract of *V. denticulata* was reported to show the antibacterial and antifungal activities [20,21]. Our previous work revealed that *V. denticulata* had a few antibacterial agents [22]. Based on these studies, *V. denticulata* could be a potential source of medicinally useful compounds, especially antimicrobial and antifungal agents. This work explores antibacterial agents in crude extracts and fractions of *V. denticulata* using UHPLC-ESI-QTOF-MS/MS analysis, as well as a molecular networking. It is known that different parts of plants may have different chemical constituents and thus exerting different pharmacological effects [23]. We report herein antibacterial and antifungal compounds in both bark and trunk of a plant, *V. denticulata*. 

## 2. Results and Discussions

### 2.1. Dereplication of Compounds from Crude Extracts of V. denticulata and Guided Isolation by UHPLC-ESI-QTOF-MS/MS-Based Molecular Networking

Fresh trunk and bark of *V. denticulata* were sequentially extracted with methanol (MeOH) and dichloromethane (CH_2_Cl_2_). Both MeOH and CH_2_Cl_2_ crude extracts were analyzed by UHPLC-ESI-QTOF-MS/MS. In this research, there were two scan types; first, LC-MS scans a total ion chromatogram (TIC) and base peak chromatogram (BPC). Both positive and negative MS ionization modes were performed because some classes of compounds such as sesquiterpenes and thiophenes were well-detected in a positive ionization mode, whereas flavonoids, phenolic acids and quinic acid could be detected by a negative ion mode [24]. Besides, the mechanism of fragmentation of positive and negative ion modes was dissimilar and they may afford supplementary structural information [25]. Overlay of TIC chromatograms of MeOH and CH_2_Cl_2_ crude extracts of *V. denticulata* is shown in Figure S1, Supplementary Materials. Second, auto-MS^2^ was performed in which the most predominant MS^1^ ions are chosen for MS^2^ fragmentation. From MS/MS spectra, the chemical constituents in crude extracts of *V. denticulata* were tentatively identified; they are listed in Table 1. The putative known and unknown compounds were annotated by the Agilent MassHunter METLIN Metabolomics Database, the Human Metabolome Database (https://hmdb.ca/) and online database Metlin (http://metlin.scripps.edu/index.php), as well as by comparison with standard compounds. The present work has seven standard compounds including (+)-*R*-ventilagolin, emodin, rutin, naringenin, 6-hydroxy flavone, chrysin and (+)-catechin.

**Figure 1:**
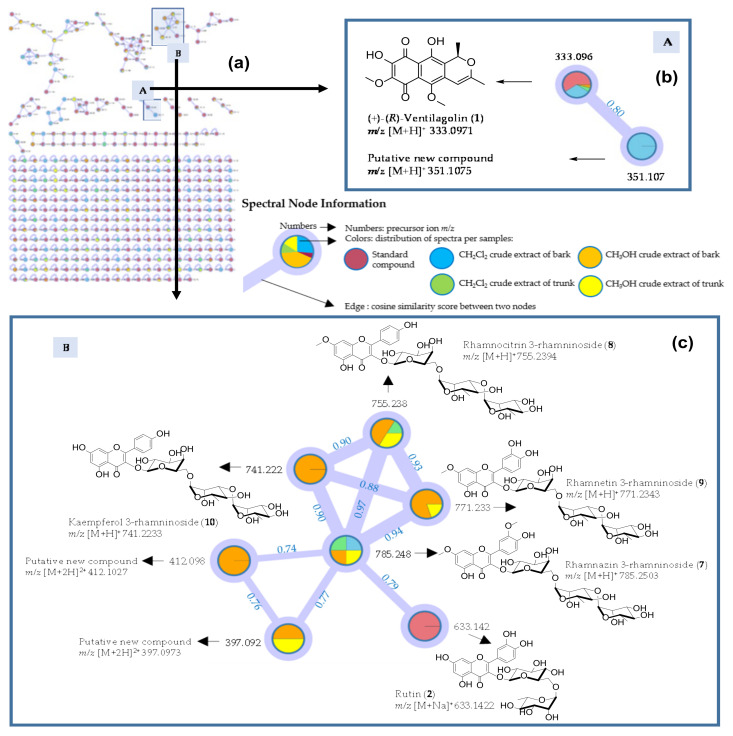
Molecular networking of crude extracts of *V. denticulata* as a complementary method for the dereplication strategy: (**a**) Molecular networking of crude extracts in a positive ionization mode; (**b**) Molecular networking connected to (+)-(*R*)-ventilagolin (**1**) and a putative new naphthalene derivative found in CH_2_Cl_2_ crude extract of bark; (**c**) Molecular networking connected to rutin (**2**) and other flavonol glycosides found in MeOH crude extract of bark.

As shown in Table 1, several compounds in crude extracts were identified in either positive or negative ionization mode. There are 93 tentatively identified compounds listed in Table 1; these metabolites have been reported as plant metabolites. Among the compounds identified in Table 1, emodin, physcion, ventilagodenin A and (+)-(*R*)-ventilagolin previously isolated by our group [22] were indeed found in crude extracts of *V. denticulata* and they underwent MS/MS fragmentation in both positive and negative ionization modes. We performed further analysis using the GNPS website; all acquired MS/MS data were converted into MzXML as an open file format by ProteoWizard. Then, the converted data were uploaded to create molecular networking on the GNPS website (http://gnps.ucsd.edu). All molecular networking data obtained from the GNPS system were imported to Cytoscape 3.7.2 version, in order to visualize and simplify molecular networking in one display. The node colors were set and they represented MS/MS data of compounds present in crude extracts or standard compounds. Cytoscape was used for rapid analysis of the whole profile of metabolites in all crude extracts, as well as for the correlation between standard compounds and their analogs. Result of the molecular networking of crude extracts in a positive mode is shown in Figure 1a, while that of a negative ionization mode is in Supplementary data (Figure S2); they are used as a complementary method for the dereplication strategy.

We employed a molecular networking for the investigation of a profile of chemical constituents in crude extracts of *V. denticulata*, basically with that of crude extracts in a positive ionization mode (Figure 1a). Colors for MeOH extracts of bark and trunk, as well as three crude extracts of bark and trunk, are depicted in Figure 1. In the present work, (+)-(*R*)-ventilagolin (**1**), a naphthalene derivative, was used as a standard compound (purple color, Figure 1b) and it found in MeOH and CH_2_Cl_2_ crude extracts of bark and CH_2_Cl_2_ crude extract of trunk but not in MeOH crude extract of trunk (Table 1). The molecular networking of (+)-(*R*)-ventilagolin (**1**) is in a cluster A (Figure 1b). Rutin (**2**), a flavonol glycoside, was also used as a standard compound and its molecular networking is in a cluster B, as shown in Figure 1c. The dereplication by MS/MS based molecular networking in a positive ionization mode also suggested the presence of a potential new naphthalene derivative (Figure 1b) and flavonol glycoside derivatives (Figure 1c), by inspecting nodes in the clusters connected to (+)-(*R*)-ventilagolin (**1**) and rutin (**2**), respectively. 

**Figure 2:**
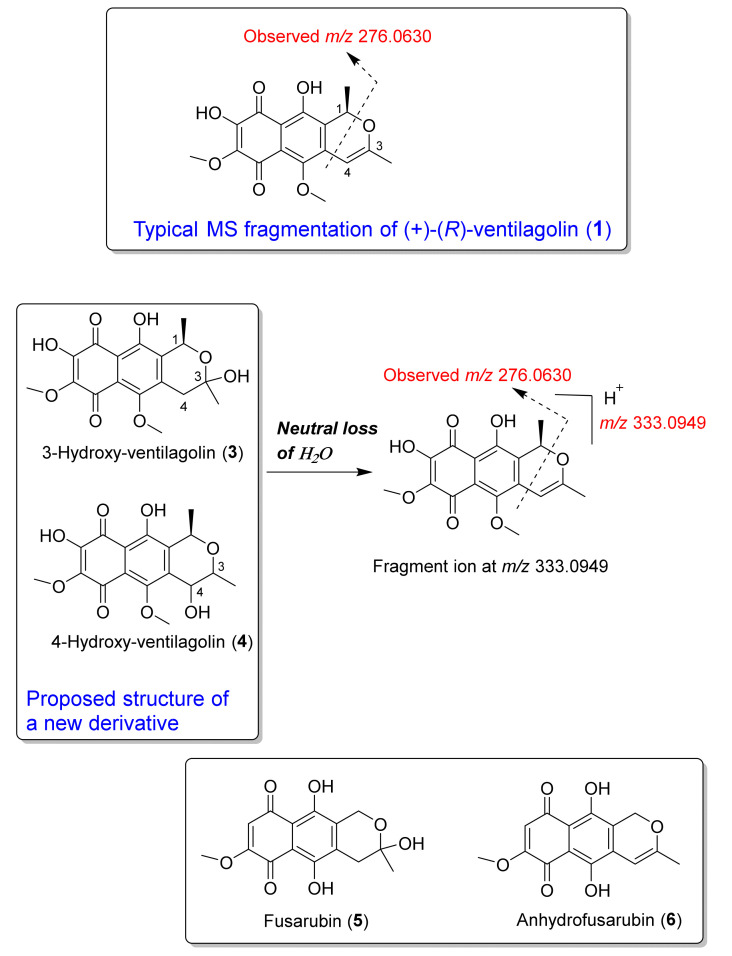
Typical MS fragmentation of (+)-(*R*)-ventilagolin (**1**); possible structure and MS fragmentations of a new derivative, 3-hydroxy-ventilagolin (**3**) or 4-hydroxy-ventilagolin (**4**); and structures of fusarubin (**5**) and anhydrofusarubin (**6**).

The molecular networking of (+)-(*R*)-ventilagolin (**1**) (*m/z* 333.0971 [M+H]^+^) (cluster A; Figure 1b) showed the node of MS/MS spectra related to the ion at *m/z* 351.1075 [M+H]^+^ with cosine similarity score of 0.80. A putative unknown compound observed at *m/z* 351.1075 [M+H]^+^ had a mass difference of 18 from (+)-(*R*)-ventilagolin (**1**) (*m/z* 333.0971 [M+H]^+^, calcd for [C_17_H_16_O_7_ + H]^+^, 333.0974, Δ*_m__/z_* = 0.90 ppm), suggesting that a putative new compound has an additional hydroxyl group. The tentative new derivative had the observed ion at *m/z* 351.1075 [M+H]^+^, calcd for [C_17_H_18_O_8_ + H]^+^, 351.1080, Δ*_m__/z_* = 1.42 ppm and thus having the molecular formula of C_17_H_18_O_8_. MS/MS spectra of both (+)-(*R*)-ventilagolin (**1**) and a putative new derivative showed the ions at *m/z* 276 and 259 (Figures S3 and S4, Supplementary Materials); a typical MS/MS fragmentation of (+)-(*R*)-ventilagolin (**1**) is depicted in Figure 2, showing the ion at *m/z* 276.0630 of [C_14_H_12_O_6_]^+^, 276.0628, Δ*_m__/z_* = 0.72 ppm. Based upon the typical MS fragmentation of (+)-(*R*)-ventilagolin (**1**), the tentative structure of a new derivative observed at *m/z* 351.1075 [M+H]^+^ (cluster A; Figure 1b) is proposed to be either 3-hydroxy-ventilagolin (**3**) or 4-hydroxy-ventilagolin (**4**), as shown in Figure 2. MS/MS spectrum (Figure S4, Supplementary Materials) of a putative new compound showed that it underwent neutral loss of water, giving a fragment ion at *m/z* 333.0949 [M+H]^+^, calcd for [C_17_H_16_O_7_ + H]^+^, 333.0974, Δ*_m__/z_* = 7.50 ppm (Figure 2), which is of (+)-(*R*)-ventilagolin (**1**), which in turn, fragmented to the ion at *m/z* 276.0630, calcd for [C_14_H_12_O_6_]^+^, 276.0628, Δ*_m__/z_* = 0.72 ppm (Figure 2 and Figure S4) that is a typical MS fragmentation for this compound class. Unfortunately, we could not isolate the putative new derivative for detailed NMR analysis. It is worth mentioning that 3-hydroxy-ventilagolin (**3**) has a similar structural feature to a fungal pigment, fusarubin (**5**) (Figure 2) [26,27], which also has an anhydro derivative, anhydrofusarubin (**6**) [27] (Figure 2), whose structure is similar to that of (+)-(*R*)-ventilagolin (**1**). By analogy to the structures of fusarubin (**5**) and anhydrofusarubin (**6**), the putative new compound is possibly 3-hydroxy-ventilagolin (**3**) (Figure 2).

**Figure 3:**
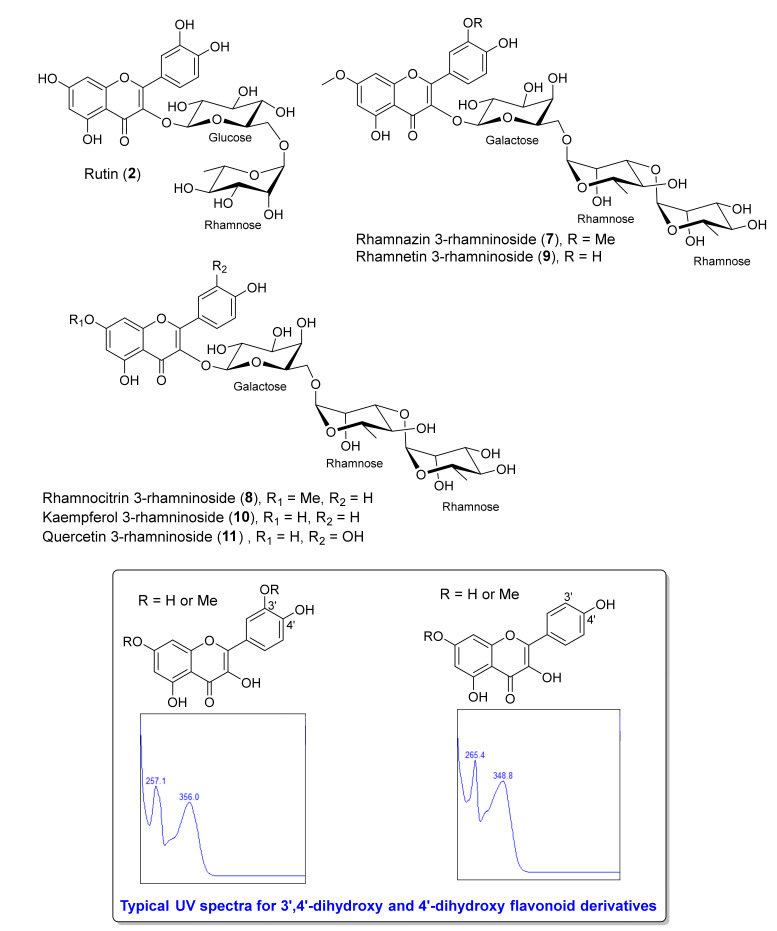
Structures of a standard flavonol glycoside, rutin (**2**) and flavonol glycosides **7**–**11**; and typical UV spectra for 3′,4′-dihydroxy and 4′-dihydroxy flavonoid derivatives showing λ_max_ at 356 nm and 348 nm, respectively.

In a cluster B (Figure 1c), node of MS/MS spectra connected to rutin (**2**) (*m/z* 633.1422 [M+Na]^+^), a standard compound, possessed a precursor ion of xanthorhamnin C or rhamnazin 3-rhamninoside (**7**) (Figure 1c and Figure 3) at *m/z* 785.2503 [M+H]^+^ with a cosine similarity score of 0.79. Rhamnazin 3-rhamninoside (**7**) was isolated and characterized by analysis of 1D and 2D NMR spectroscopy (^1^H, ^13^C NMR and MS spectra are in Figures S10–S12, Supplementary Materials). Spectroscopic data of rhamnazin 3-rhamninoside (**7**) were in good agreement with those reported in the literature [28]. Rhamnazin 3-rhamninoside (**7**) had related precursor ions at *m/z* 755.2394 [M+H]^+^ with a cosine similarity score of 0.97, at *m/z* 771.2343 [M+H]^+^ with a cosine similarity score of 0.94 and at *m/z* 741.2233 [M+H]^+^ with a cosine similarity score of 0.90, which are catharticin or rhamnocitrin 3-rhamninoside (**8**), xanthorhamnin B or rhamnetin 3-rhamninoside (**9**) and kaempferol 3-rhamninoside (**10**), respectively (Figure 1c and Figure 3). Flavonol glycosides **8**-**10** were also isolated and structurally characterized by analysis of 1D and 2D NMR spectroscopy (^1^H, ^13^C NMR and MS spectra are in Figures S13–S21, Supplementary Materials). Spectroscopic data of compounds **8**–**10** were identical to those published in the literature [28,29,30,31]. Moreover, flavovilloside or quercetin 3-rhamninoside (**11**) (Figure 3) was also obtained during the isolation of flavonol glycosides **7**–**10**; its ^1^H, ^13^C NMR and MS spectra are in Figures S22–S24, Supplementary Materials). Spectroscopic data of quercetin 3-rhamninoside (**11**) were in good agreement with published values [28]. However, quercetin 3-rhamninoside (**11**) was not detected by LC-MS/MS analysis; therefore, it is not listed in Table 1 and it does not appear in the molecular networking of a cluster B (Figure 1c) in spite of being a derivative of rutin (**2**). The sugar in a standard flavonol glycoside, rutin (**2**), is glucose, while that in the isolated flavonol glycosides **7**–**11** is galactose (Figure 3). In a cluster B (Figure 1c), compounds with the ions at *m/z* 412.1027 [M+2H]^2^^+^ and 397.0973 [M+2H]^2^^+^ had related precursor ions to rhamnazin 3-rhamninoside (**7**) and they were considered as potential new compounds. Unfortunately, attempts to isolate these compounds for detailed NMR analysis have met with failure. It is worth mentioning that HPLC-PDA method could be used to distinguish 3′,4′-dihydroxy flavonoid (i.e., flavonol glycosides **7**, **9** and **11**) from 4′-dihydroxy flavonoid derivative (i.e., flavonol glycosides **8** and **10**) (Figure 3). 3′,4′-Dihydroxy flavonoid had a typical λ_max_ at 356 nm in the UV spectrum, while 4′-dihydroxy flavonoid derivative showed a typical λ_max_ at 348 nm (Figure 3). 

**Figure 4:**
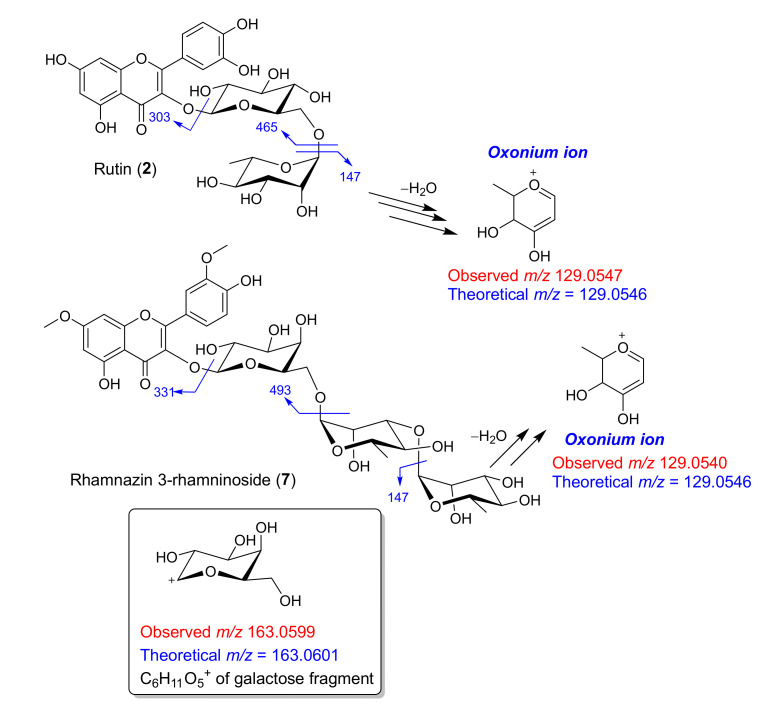
Typical MS fragmentation a standard compound, rutin (**2**), MS fragmentation of rhamnazin 3-rhamninoside (**7**) and an oxonium ion of rhamnose at *m/z* 129.0 and a galactose fragment ion at *m/z* 163.

Analysis of MS/MS spectrum (Figure S5, Supplementary Materials) of a standard flavonol glycoside, rutin (**2**), revealed losses of glucose and rhamnose, showing the ions resulting from the loss of rhamnose (at *m/z* 465 from loss of 146) and of glucose-rhamnose (at *m/z* 303) (Figure 4). Loss of 146 of rhamnose gave the ion at *m/z* 465 and such loss was previously observed for flavonoid glycosides [25] and triterpene saponins [32]. Interestingly, the ion abundance at *m/z* 147.0653, calcd [C_6_H_11_O_4_ +H]^+^, which was of a rhamnose fragment, was 4 times lower than that of the ion at *m/z* 129.0547 (Figure S5, Supplementary Materials). Careful analysis revealed that the observed ion at *m/z* 129.0547 could be of an oxonium ion of a sugar rhamnose, which was from a neutral loss of water of a rhamnose fragment at *m/z* 147.0653, as depicted in Figure 4. The observed ion at *m/z* 129.0547 and the calculated *m/z* value of 129.0546 for C_6_H_9_O_3_^+^ with the mass difference of 0.26 ppm (Figure 4) readily confirmed the structure of an oxonium ion of rhamnose. Normally, oxonium ions of sugar are observed in MS/MS spectra of glycosides [33] and they are useful ions for sugar identification in modern glycoproteomic research [34,35]. To the best of our knowledge, this is the first report on the oxonium ion of rhamnose, C_6_H_9_O_3_^+^ at *m/z ca* 129.05 and it is possibly used as a characteristic fragment ion for rhamnose in mass spectrometry.

Molecular networking of rutin (**2**) (cluster B, Figure 1c) had the precursor ion of xanthorhamnin C or rhamnazin 3-rhamninoside (**7**) at *m/z* 785. 2503 [M+H]^+^ with a cosine similarity score of 0.79. MS/MS spectrum (Figure S6, Supplementary Materials) of rhamnazin 3-rhamninoside (**7**) showed fragment ions analogous to that of rutin (**2**), that is, loss of rhamnose giving the oxonium ion at *m/z* 129.0540 (Figure 4). The major fragments at *m/z* 493 and 331 due to loss of rhamnose-rhamnose followed by loss of galactose were observed in the MS/MS spectrum of rhamnazin 3-rhamninoside (**7**) (Figure 4 and Figure S6). Unlike rutin (**2**), the MS/MS spectrum rhamnazin 3-rhamninoside (**7**) displayed the ion at *m/z* 163.0599 (Figure 4 and Figure S6), C_6_H_11_O_5_^+^, calcd for 163.0601 (mass difference of 1.22 ppm), which was likely to be a fragment of galactose, C_6_H_11_O_5_^+^. Flavonol glycosides **8**–**10** have galactose in their molecules; indeed, the MS/MS spectra of these compounds showed a fragment ion of galactose at *m/z* 163 (Figures S7–S9, Supplementary Materials). While glucose in rutin (**2**) does not have a fragment ion at *m/z* 163, galactose in flavonol glycosides **8**–**10** shows the characteristic fragment ion at *m/z* 163; therefore, the fragment ion at *m/z* 163 might be used for the identification of galactose in mass spectrometry-based analysis of glycosides or oligosaccharide chains attached to biomolecules (i.e., glycoproteins).

**Figure 5:**
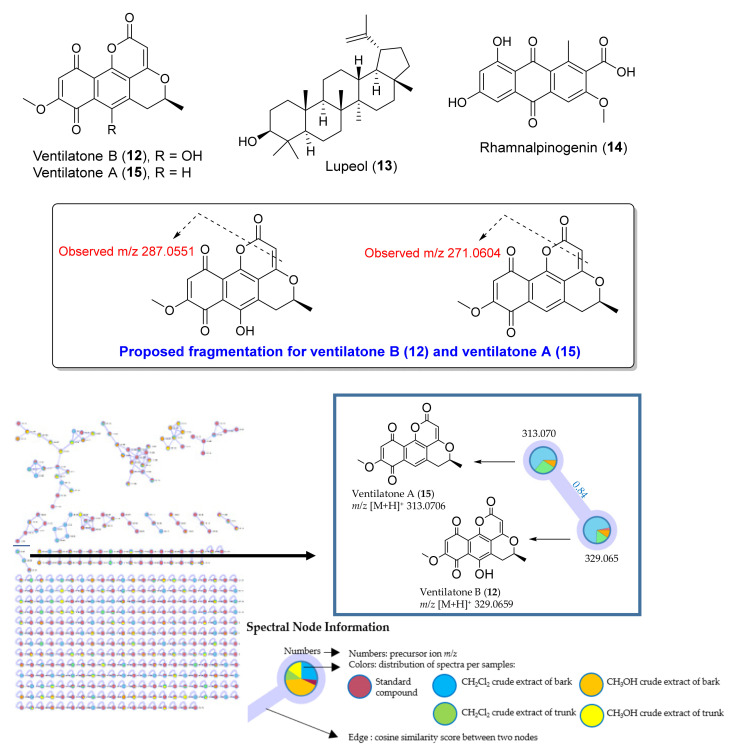
Structures of ventilatone B (**12**), lupeol (**13**), rhamnalpinogenin (**14**) and ventilatone A (**15**); proposed fragmentations and molecular networking of ventilatone B (**12**) and ventilatone A (**15**).

In the present study, ventilatone B (**12**), a triterpene lupeol (**13**) and ventilatone A (**15**) (Figure 5) were also isolated from a CH_2_Cl_2_ extract of bark of *V. denticulata*. Ventilatones B (**12**) and A (**15**) are benzisochromanquinone, which were previously isolated from *V. calyculata* [36]. Structures of ventilatone B (**12**), lupeol (**13**) and ventilatone A (**15**) were characterized by analysis of NMR spectroscopy (^1^H, ^13^C NMR and MS spectra are in Figures S27–S34, Supplementary Materials); their spectroscopic data were in good agreement with those reported in the literature [36,37]. Lupeol (**13**) was previously found in the plant genus *Ventilago*, for example, *V. denticulata* [38] and *V. bombaiensis* [39]. Note that rhamnalpinogenin (**14**) (Figure 5), which has the same molecular formula, C_17_H_12_O_7_, as that of ventilatone B (**12**), was tentatively identified by LC-MS/MS analysis, as revealed by both the Metlin Database and the Human Metabolome Database (Table 1, No. 70), observed at *m/z* 329.0659, calcd for 329.0656 (Δ*_m__/z_* = 1.09 ppm). However, there is a possibility that this putative compound is ventilatone B (**12**) because this benzisochromanquinone was previously isolated from *V. calyculata* [36], which is the same plant used in this work (*V. denticulata* formerly known as *V. calyculata*). The MS/MS spectrum (Figure S25, Supplementary Materials) of the compound with the molecular formula C_17_H_12_O_7_ suggested that it is more likely to be ventilatone B (**12**) because of the loss of C_2_HO, giving the fragment ion at *m/z* 287.0551(Figure 5). In the case of rhamnalpinogenin (**14**), it should undergo a neutral loss of CO_2_ (44 amu) because it has a carboxylic group in its molecule (Figure 5) but none of the fragment ions were observed from the loss of CO_2_. Moreover, the molecular networking of ventilatone B (**12**) is related to the compound with the *m/z* 313.0706 with a cosine similarity score of 0.84 (Figure 5). Analysis of MS/MS spectrum (Figure S26, Supplementary Materials) of the compound with the *m/z* 313.0706 revealed that this compound is likely to be ventilatone A (**15**), which undergoes the loss of C_2_HO, giving the fragment ion at *m/z* 271.0604 (Figure 5) that is analogous to ventilatone B (**12**). Note that the compound at the *m/z* 313.0706 was also listed in Table 1 (No. 61) and it was proposed to be aloe emodin w-acetate by Metlin Database and Human Metabolome Database. However, the MS/MS fragmentation suggested that this compound should be ventilatone A (**15**), not aloe emodin w-acetate.

### 2.2. Structure Elucidation of Ventilatone C (**16**)

**Figure 6:**
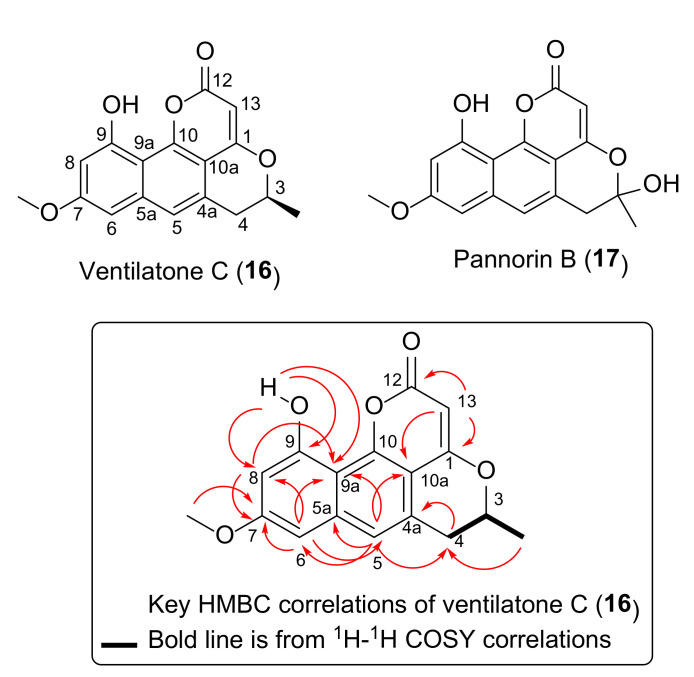
Structures of ventilatone C (**16**) and pannorin B (**17**), as well as key HMBC and ^1^H-^1^H COSY correlations of ventilatone C (**16**). HMBC correlations are from proton(s) to carbon.

In the present work, a new compound—named ventilatone C (**16**)—was isolated from a CH_2_Cl_2_ extract of a bark of *V. denticulata* (Figure 6). Structure elucidation of ventilatone C (**16**) was performed by analysis of NMR and MS data. Ventilatone C (**16**) was obtained as yellow amorphous solid and its molecular formula, C_17_H_14_O_5_, was obtained from ESI-HRMS, showing a pseudo-molecular ion at *m****/****z* 299.0917 (M**+**H)**^+^**, calcd for C_17_H_15_O_5_, *m****/****z* 299.0919. ^1^H and ^13^C NMR spectra of ventilatone C (**16**) were similar to those of ventilatones B (**12**) and A (**15**), particularly on the signals for the fragment of 3-Me/H-3/H-4. ^1^H NMR spectrum in CDCl_3_ of 16 showed signals of a hydroxyl proton at δ_H_ 8.81 (br s), three aromatic protons at δ_H_ 7.24 (H-5), 6.72 (H-6 and H-8), one olefinic proton at δ_H_ 5.74, sp^3^ methine at δ_H_ 4.53 (H-3), non-equivalent methylene at δ_H_**** 3.15 and 3.00 (H-4) and two methyl groups at δ_H_ 3.91 (7-OMe) and 1.56 (3-Me) (Table 2). ^1^H NMR signals in CDCl_3_ for H-6 and H-8 were overlapping at δ_H_**** 6.72, however, these signals were clearly observed in acetone-*d_6_* as a doublet at δ_H_ 6.94 (H-6) and 6.64 (H-8) and the J = 2.3 Hz (Table 2) indicated the presence of *meta* coupling aromatic protons in **16**. ^13^C NMR and DEPT spectra of ventilatone C (**16**) showed seventeen signals attributable to two methyl, five methine, one methylene, nine quaternary carbons. ^1^H-^1^H COSY spectrum of **16** established the fragment of 3-Me/H-3/H-4 (as a bold line in Figure 6). HMBC spectrum of 16 showed the correlations from 3-Me to C-4; H-4 to C-4a; H-5 to C-4, C-5a, C-6, C-9a and C-10a; H-6 to C-5, C-7, C-8 and C-9a; H-8 to C-7 and C-9a; and H-13 to C-1, C10a and C-12 (Figure 6). The HMBC correlation from 7-OMe to C-7 placed the methoxy group at the position C-7, while that from 9-OH proton to C-8, C-9 and C-9a assigned the OH group at C-9. Ventilatone C (**16**) had a positive optical rotation value ([α]^25^_D_ +2.60 (*c* 0.25, CHCl_3_)) similar to those of ventilatones B (**12**) ([α]^25^_D_ +30.62 (*c* 0.5, CHCl_3_) and A (**15**) ([α]^25^_D_ +7.85 (*c* 0.2, CHCl_3_)), both having *3S* stereochemistry, therefore, the C-3 configuration in **16** was assigned to be *S*. Based on these spectroscopic data, the structure of ventilatone C (**16**) was established as shown in Figure 6. Ventilatone C (**16**) has a structure closely related to pannorin B (**17**) [40] (Figure 6). However, pannorin B (**17**) was previously isolated from an endophytic fungus *Penicillium* sp. [40] and its biosynthetic pathway was proposed to be related to that of pannorin [41]. Interestingly, a fungal metabolite, pannorin B (**17**), shares the same chemical skeleton as that of ventilatones B (**12**) and A (**15**), which were isolated from the plant, *V. calyculata* [36].

### 2.3. Antibacterial and Antifungal Activities of Crude Extracts, Fractions and Isolated Compounds

As mentioned in the introduction part, local people in the West Midnapore district of West Bengal, the Eastern State of India, use the plant *V. denticulata* for the treatment of wound infection [15]. Bacteria found in wound infection were *Staphylococcus aureus*, *Pseudomonas aeruginosa*, *Escherichia coli*, *Bacillus cereus* and *Salmonella enterica* serovar Typhimurium [16,17,18], while the fungus *Candida* *albicans* was found in wound infection in diabetic foot ulcers [19]. Therefore, this research evaluated antibacterial and antifungal activities of crude extracts against *S. aureus*, *P. aeruginosa*, *E. coli*, *B. cereus*, *S. enterica* and *C*. albicans (Table 3). Methanol crude extracts of bark (MB) and trunk (MT) exhibited antibacterial activity against *B. cereus*, *S. aureus*, *E. coli*, *S. enterica* and *P. aeruginosa* with inhibition zones of 7–13 mm, 14–15 mm, 8 mm, 7–14 mm and 7–10 mm, respectively (Table 3). A CH_2_Cl_2_ crude extract of bark (DB) displayed antibacterial activity against *B. cereus*, *S. aureus*, *E. coli*, *S. enterica* and *P. aeruginosa* with inhibition zones of 21 mm, 18 mm, 9 mm, 19 mm and 8 mm, respectively (Table 3), while a CH_2_Cl_2_ crude extract of trunk (DT) showed antibacterial activity against *S. aureus* with inhibition zone of 13 mm (Table 3). Crude extracts, MB, DB and MT exhibited antifungal activity against *C. albicans* with inhibition zones of 13 mm, 16 mm and 8 mm, respectively (Table 3). Fractions FM1-FM6 obtained from HPLC isolation of MeOH crude extract of bark were evaluated for antibacterial and antifungal activities. Fractions FM1-FM3 showed antibacterial activity toward the bacterial strains tested with inhibition zones of 8–14 mm, except that the fraction FM1 did not inhibit the growth of *S. enterica* (Table 3). Fraction FM4 exhibited the activity against *S. enterica* with inhibition zone of 14 mm, while fraction FM6 displayed the activity toward bacteria *E. coli* and *S. enterica* with inhibition zone of 9 mm (Table 3). Fraction FM2 exhibited antifungal activity with inhibition zone of 10 mm. Fractions FD1-FD6 from fractionation of a CH_2_Cl_2_ crude extract of bark displayed antibacterial activities toward the bacterial strains tested with inhibition zones of 9–30 mm, while the fractions FD1 and FD6 showed antifungal activity against *C. albicans* with inhibition zones of 17 and 9 mm, respectively (Table 3). 

Flavonoid glycosides **7**–**11** were isolated from MeOH crude extract of bark of *V. denticulata* and they were evaluated for antibacterial and antifungal activities (Table 3). Rhamnazin 3-rhamninoside (**7**) exhibited antibacterial activity against *S. aureus* with inhibition zone of 11 mm, while catharticin or rhamnocitrin 3-rhamninoside (**8**) showed the activity toward *B. cereus* and *E. coli* with respective inhibition zones of 10 mm and 11 mm (Table 3). Xanthorhamnin B or rhamnetin 3-rhamninoside (**9**) displayed antibacterial activity against *B. cereus*, *S. aureus* and *P. aeruginosa* with respective inhibition zones of 9 mm, 9 mm and 13 mm (Table 3). Kaempferol 3-rhamninoside (**10**) and flavovilloside or quercetin 3-rhamninoside (**11**) exhibited antibacterial activity against *B. cereus*, *S. aureus* and *E. coli* with inhibition zones of 9 mm, 11–14 mm and 10–12 mm, respectively (Table 3) but they did not possess antifungal activity toward *C. albicans*. Rhamnazin 3-rhamninoside (**7**), rhamnocitrin 3-rhamninoside (**8**) and xanthorhamnin B or rhamnetin 3-rhamninoside (**9**) displayed antifungal activity against *C. albicans* with inhibition zones of 8 mm, 12 mm and 6 mm, respectively (Table 3). To our knowledge, this is the first report on antibacterial and antifungal activities of flavonoid glycosides **7**–**11**. Recently, xanthorhamnin B or rhamnetin 3-rhamninoside (**9**) was found to have antioxidative and radioprotective properties [42]. Rhamnazin 3-rhamninoside (**7**), rhamnocitrin 3-rhamninoside (**8**) and rhamnetin 3-rhamninoside (**9**) were reported to exhibit antioxidant and free radical-scavenging activities [43]. Glycoside derivatives of kaempferol were previously found to exhibit potent antibacterial activity against methicillin-resistant *S. aureus* and vancomycin-resistant enterococci [44]. Previously, kaempferol, an aglycone of **10**, was found to exhibit antibacterial activity toward *E. coli* and it acted as DNA gyrase inhibitor [45], while quercetin, an aglycone of **11**, exhibited antibacterial and antioxidant activities [46], targeting D-alanine:D-alanine ligase [47]. Interestingly, quercetin diacylglycoside derivatives displayed antibacterial activity by inhibition of DNA gyrase and topoisomerase IV [48]. 

Ventilatone B (**12**), lupeol (**13**), ventilatones A (**15**) and ventilatone C (**16**) isolated from a CH_2_Cl_2_ crude extract of bark of *V. denticulata* displayed antibacterial and antifungal activities (Table 3). Ventilatone B (**12**) exhibited antibacterial activity against *B. cereus*, *S. aureus* and *S. enterica* with inhibition zones of 11 mm, 11 mm and 18 mm, respectively and it also showed antifungal activity against *C. albicans* with inhibition zone of 12 mm (Table 3). Lupeol (**13**) displayed antibacterial activity against *S. aureus* with inhibition zone of 7 mm (Table 3) but did not exhibit antifungal activity. This is the first report on antibacterial and antifungal activities of ventilatone B (**12**). Lupeol (**13**) was previously reported to exhibit antibacterial activity against human pathogenic bacteria [49]. Ventilatones A (**15**) exhibited antibacterial activity against *B. cereus*, *S. aureus* and *S. enterica* with inhibition zones of 13 mm, 17 mm and 18 mm, respectively, while ventilatone C (**16**) displayed antibacterial activity against *B. cereus*, *S. aureus* and *S. enterica* with inhibition zones of 13 mm, 13 mm and 14 mm, respectively (Table 3).

### 2.4. Dereplication of Antibacterial and Antifungal Constituents from HPLC Fractions of V. denticulata

Fractions FM1-FM3 and FD1-FD4 from HPLC separation showed antibacterial and antifungal activities (Table 3); therefore, efforts have been made to identify the compounds in these HPLC fractions. Since the tentatively identified compounds (Table 1) in *V. denticulata* were obtained from LC-MS/MS analysis using Metlin Database and Human Metabolome Database, as well as standard compounds, we employed the accurate mass from ESI-HRMS to identify the compounds in fractions possessing antibacterial and antifungal activities. The ranges of mass difference (Δ) between the observed and calculated m/z values for each compound were ca 0.55–2.42 ppm, which is less than 5 ppm and thus giving the molecular formula of the compounds. ESI-HRMS analysis revealed that the fraction FM1 contained kaempferol, chrysoeriol, kaempferol 3-rhamninoside (**10**), isopimpinellin, 3-hydroxyphloretin, rhamnocitrin 3-rhamninoside (**8**), rhamnetin 3-rhamninoside (**9**) and rhamnazin 3-rhamninoside (**7**) (Table 4). Antibacterial and antifungal activities of flavonoid glycosides **7–10** are already presented in Table 3. Kaempferol was previously found to be an antibacterial agent [45,50]. Antibacterial activity of a flavonoid, chrysoeriol, was recently reported [51,52], while antibacterial and antifungal activities of isopimpinellin were already established [53]. Therefore, all compounds in the fraction FM1 have antibacterial activity, except 3-hydroxyphloretin. Fraction FM2 contained rhamnetin, luteolin and 3,5,7-trihydroxy-4′,6-dimethoxyflavanone (Table 4), as revealed by ESI-HRMS analysis. Rhamnetin was previously found to have antifungal activity and it is a phytoalexin in plants [54], while luteolin was formerly found to exhibit antifungal activity [55]. Luteolin is a known antibacterial agent [56,57] and it is a lead compound for the synthesis of antibacterial derivatives [58]. 3,5,7-Trihydroxy-4′,6-dimethoxyflavanone was formerly isolated from a plant, Prunus domestica [59] but it has never been evaluated for any biological activity. ESI-HRMS analysis showed that the fraction FM3 had emodin, rhamnocitrin and palmidin A (Table 4). Antibacterial activity of emodin was reported by our group [22] and emodin was previously found to inhibit growth of the bacterium Haemophilus parasuis, a causative agent of Glässer’s disease and thus being a potential drug candidate for treating Glässer’s disease [60]. Previously, antibacterial activity of rhamnocitrin was reported [61,62], while the activity of palmidin A has never been reported to date. Overall, eleven antibacterial compounds including flavonoid glycosides **7**–**10**, kaempferol, chrysoeriol, isopimpinellin, rhamnetin, luteolin, emodin and rhamnocitrin are tentatively identified from the active fractions FM1-FM3, suggesting that the dereplication technique by LC-MS/MS analysis rapidly identifies antibacterial agents in extracts and fractions. In the present work and our previous report [22], antibacterial glycosides **7**–**10** and emodin were isolated from *V. denticulata*.

ESI-HRMS analysis for compounds in fractions FD1-FD4 obtained from HPLC separation was performed (Table 4). Fraction FD1 contained eriodyctiol, cartorimine, chrysoeriol, rhamnetin, 3-hydroxyphloretin, xanthotoxol glucoside and furocoumarinic acid glucoside (Table 4); among these compounds, chrysoeriol and rhamnetin were previously found to have antibacterial and antifungal activities [51,52,54]. ESI-HRMS analysis revealed that the fraction FD2 contained ventilagodenin A, physcion, rhamnocitrin, ventilatone A (**15**), 3′,7-dihydroxy-4′,8-dimethoxyisoflavone, rhamnazin, 3,5,7-trihydroxy-4′,6-dimethoxyflavanone and ventilatone B (**12**) (Table 4). Ventilagodenin A was found to be an antibacterial agent by our group [22], while ventilatones B (**12**) and A (**15**) were isolated in the present work; their antibacterial and antifungal activities are reported in Table 3. Rhamnocitrin and rhamnazin were formerly found as antibacterial agents [61,62]. FD3 was found to contain afzelechin, (+)-(R)-ventilagolin and mukurozidiol (Table 4); among these compounds, (+)-(R)-ventilagolin (**1**) was found to be an antibacterial compound by our research group [22], whereas mukurozidiol or byakangelicin was previously reported to have antibacterial activity [63]. FD4 contained emodin, 6α-hydroxymaackiain, 2′,3,5-trihydroxy-5′,7-dimethoxyflavanone and palmidin A (Table 4); however, only emodin was found to be an antibacterial agent [22,60]. Overall, ten antibacterial compounds including chrysoeriol, rhamnetin, ventilagodenin A, rhamnocitrin, rhamnazin, mukurozidiol, emodin, (+)-(R)-ventilagolin (**1**) and ventilatones B (**12**) and A (**15**) were identified from fractions FD1-FD4.

## 3. Materials and Methods

### 3.1. General Experimental Procedures

UHPLC-MS/MS was carried out using Agilent 1290 infinity II connected to Agilent 6545 QTOF. HPLC column is ACE Excel C_18_ AR (100 × 2.1 mm, 1.7 µm) column. MS data were processed using MassHunter data acquisition software. ESI-HRMS spectra were acquired from Bruker MicroTOF mass spectrometer processed using Bruker daltonics data analysis 3.3 software. HPLC was performed by Waters 1525 binary pump connected to a 2998 photodiode array detector. A semi-preparative column is SunFire C_18_ (19 × 250 mm, 5.0 µm); the HPLC chromatogram was processed by Empower 2 software. NMR spectra were obtained from Bruker Avance 400 MHz NMR spectrometer, processed by TopSpin software. Sephadex LH-20 was packed for column chromatography. Specific optical rotation of compound **16** was obtained from a JASCO P-1020 polarimeter. 

Methanol hypergrade LiChrosolv (LC-MS grade) and formic acid LiChropur (LC-MS grade) were used as the mobile phase for LC-MS analysis. Methanol-*d*_4_, CDCl_3_, acetone-*d_6_* were used as solvents for NMR analysis. 

### 3.2. Plant Materials and Extraction of Plant

The plant *Ventilago denticulata* was collected from Prachin Buri province, Thailand. It was characterized by Forest Herbarium, Bangkok, Thailand, in April 2019. *V. denticulata* (Voucher specimen number: CRI712) was deposited at Chulabhorn Research Institute (CRI), Thailand. Fresh trunks of *V. denticulata* were separated from their barks and then cut into small pieces (around ± 0.5 cm). Fresh plant samples were sequentially with MeOH and CH_2_Cl_2_; this is because fresh samples contain water and MeOH, a water-miscible solvent, was first used as a solvent. Trunk (1.7 kg) was macerated sequentially with MeOH (2 × 1.5 L) and CH_2_Cl_2_ (2 × 1.5 L) at room temperature for 2 days to give 34.66 g of MeOH crude extract of trunk and 15.03 g of CH_2_Cl_2_ crude extract of trunk. Bark (0.5 kg) was macerated sequentially with MeOH (2 × 1.0 L) and CH_2_Cl_2_ (2 × 1.0 L) at room temperature for 2 days to give 24.38 g of MeOH crude extract of bark and 1.24 g of CH_2_Cl_2_ crude extract of bark. All crude extracts were stored and kept in a freezer (−18 °C). 

### 3.3. Crude Extract and Preparation of Standard Compounds for LC-MS/MS Analysis

1 mg of each crude extract was dissolved in 1 mL of methanol to make a stock solution with a concentration of 1 mg/mL. 100 µL of each stock solution was diluted with 900 µL MeOH to obtain the final concentration of 100 µg/mL. This solution was filtered through 0.22 µm and transferred into 2 mL-LC vial.

Stock solutions of the following compounds (1 mg each), ((+)-*R-*ventilagolin, emodin, rutin, naringenin, 6-hydroxy flavone, chrysin and (+)-catechin) were dissolved in 1 mL of methanol. 100 µL of each stock solution was diluted with 900 µL methanol to obtain the final concentration of 100 µg/mL. These solutions were filtered through 0.22 µm filter and transferred into 2 mL-LC vial.

### 3.4. UHPLC-ESI-QTOF-MS/MS Conditions

Crude extracts and standard compounds were analyzed by UHPLC connected to Q-TOF MS. UHPLC column was ACE Excel C_18_ AR (100 × 2.1 mm, 1.7 µm) and a flow rate was 0.2 mL/min with an injection volume of 0.5 µL. The gradient elution was performed using the following conditions: (i) linear gradient from 40% CH_3_CN (0.1% formic acid) in H_2_O (0.1% formic acid) to 100% CH_3_CN (0.1% formic acid) for 0–25 min, (ii) isocratic elution of 100% CH_3_CN (0.1% formic acid) for 5 min (at time of 25–30 min), (iii) a linear gradient from 100% CH_3_CN (0.1% formic acid) to 40% CH_3_CN (0.1% formic acid) in H_2_O (0.1% formic acid) for 4 min (at time of 30–34 min) and (iv) equilibrium time by isocratic elution with 40% CH_3_CN (0.1% formic acid) in H_2_O (0.1% formic acid) for 6 min (at time of 34–40 min). The total run time was 40 min.

Dual AJS (Agilent Jet Stream) ESI was used as an ion source arranged with sheath gas flow of 12 L/min, capillary temperature at 325 °C, the gas flow rate of 10 L/min, sheath gas temperature of 250 °C, sheath gas flow of 12 L/min, nebulizer of 45 psig, capillary voltage of 3.5 kV, fragmentor of 150 V, skimmer of 65 V and nozzle voltage of 1 kV. MS relative threshold and MS absolute threshold were set to 0.010% and 100, respectively.

LC-MS scan total ion chromatogram (TIC) and base peak chromatogram (BPC) with a scan range of 100–1100 *m/z* and the analysis was performed in both positive and negative ionization modes. MS scan rate is 2 spectra per min. Auto-MS^2^ was performed using fixed collision energy at 20 keV, at which the most predominant MS^1^ ions are chosen for MS^2^ fragmentation. Auto-MS^2^ acquisition shows MS/MS data around 80–95% of precursor ions. The MS/MS data were acquired with a scan rate of 3 spectra per second with MS/MS scan range at 100–1100 *m/z*. Isolation width MS/MS was set at medium (*ca* 4 amu). The maximum precursor was 3 per cycle. The MS/MS relative threshold was set to 0.01% and MS/MS absolute threshold was set to 5.

The reference mass correction was performed and set as auto recalibration using a reference solution with minimal height of 1000 counts and the detection window of 100 *m/z*. The ions at *m/z* 121.0509 (purine) and *m/z* 922.0098 (HP-0921) were selected as standard ion peaks in a positive ion mode, while the ions at *m/z* 112.9856 (TFA anion) and *m/z* 1033.9881 (HP-0921 + TFA anion) were selected as standard ion peaks in a negative ion mode. In the auto MS/MS preferred/exclude table, these reference masses must be written as exclusion mass [64].

### 3.5. Molecular Networking

#### 3.5.1. Converting MS/MS Data

All acquired MS/MS data was converted into MzXML format for further analysis in the GNPS website by ProteoWizard supported by NET Framework 3.5 SP1 using the following parameters [7]:

- 32-Bit was selected for binary encoding precision and zip compression was unchecked.
- Peak picking was set as a filter to make the output data become centroid.
- MS-Levels 1 and 2 should be checked.

#### 3.5.2. Molecular Networking by GNPS (Global Natural Products Social Molecular Networking)

FTP client, WinSCP, was used to upload the converted MS/MS data to the MzXML format using the host ost ccms-ftp01.ucsd.edu; these data to system were then transferred automatically to the GNPS system. The uploaded data were available in GNPS website readily for uploading data to create the molecular networking on the GNPS website (http://gnps.ucsd.edu). 

In the basic option setting, precursor ion and fragment ion mass tolerance were set to 0.5 and 0.02, respectively. The advanced network setting systems were set to minimum pairs cos of 0.7, network TopK of 10, maximum connected component size of 100, minimum matched fragment ions of 4, the minimum cluster size of 2 [64].

For further analysis, the spectra were searched and matched toward GNP spectral library. They were set to the library search minimum matched of 4, search analog of “do search,” score threshold of 0.7, maximum analog search mass difference of 100. Cosine similarity score that shows closer score to 1 indicates higher similarity matched with the library spectra or representing identical spectra, whereas the score closer to 0 indicates no similarity. The calculation of cosine similarity was considered based on fragment ions, precursor ions and peak intensities [64].

#### 3.5.3. Visualization of Molecular Networking Using Cytoscape

The molecular networking data obtained from the GNPS system were imported to Cytoscape 3.7.2 to visualize and simplify molecular networking in one display. Cytoscape was used for analyzing the whole profile of metabolites in all crude extracts and correlation between standard compounds and their analogs [7]. 

### 3.6. Isolation of (+)-(R)-Ventilagolin (**1**), Flavonoid Glycosides (**7**–**11**), Ventilatone B (**12**), Lupeol (**13**), Ventilatone A (**15**) and Ventilatone C (**16**)

A MeOH crude extract of bark (10.23 g) was subjected to Sephadex LH-20 (5 × 55 cm) column chromatography (CC), eluted with MeOH to give 65 fractions. Fraction 9 (271 mg) and fraction 10 (206 mg) containing flavonol glycosides and they were further purified using semi-preparative C_18_ HPLC column (Sunfire 5 µm, 19 × 250 mm). The gradient elution was performed using the following conditions: (i) isocratic elution of 30% MeOH/H_2_O for 0–10 min, (ii) a linear gradient from 30% MeOH/H_2_O to 60% MeOH/H_2_O over 60 min (at time of 10–70 min), (iii) a linear gradient from 60% MeOH/H_2_O to 100% MeOH/H_2_O for 15 min (at time of 70–85 min), (iv) a further linear gradient from 100% MeOH/H_2_O to 30% MeOH/H_2_O for 5 min (at time of 85–90 min) and (v) an isocratic elution with 30% MeOH/H_2_O over 10 min (at time of 90–100 min). The total run time was 100 min. UV detector was set at 276 nm and a flow rate was 10 mL/min. The injection volume was 400 µL. This HPLC purification yielded quercetin 3-rhamninoside (**11**, t_R_ 41 min, 8.6 mg), kaempferol 3-rhamninoside (**10**, t_R_ 46 min, 13.4 mg), rhamnetin 3-rhamninoside (**9**, t_R_ 60 min, 25.5 mg), rhamnocitrin 3-rhamninoside (**8** t_R_ 66 min, 18.2 mg), rhamnazin 3-rhamninoside (**7**, t_R_ 70 min, 35.9 mg). 

A CH_2_Cl_2_ crude extract of bark (782 mg) was subjected to Sephadex LH-20 (2 × 132 cm) CC, eluted with MeOH to give 33 fractions. Fraction 7 was identified as lupeol (**13,** 3.0 mg). Fraction 23 was identified as (+)-ventilatone B (**12**, 3.7 mg). Fraction 12 (58.9 mg) containing naphthalene derivatives was further purified by semi-preparative C_18_ HPLC using a reversed-phase column (Sunfire 5 µm, 19 × 250 mm). UV detector was set at 276 nm and a flow rate was 10 mL/min. The injection volume was 400 µL. The isocratic elution was performed using 60% MeOH/H_2_O at a flowrate of 10 mL/min to give (+)-(*R*)-ventilagolin (**1**, t_R_ 9 min, 2.3 mg). An insoluble part of fraction 12 was also identified as (+)-(*R*)-ventilagolin (**1**, 24.9 mg). 

An insoluble CH_2_Cl_2_ crude extract of bark (189 mg) was purified by semi-preparative C_18_ HPLC (Sunfire 5 µm, 19 × 250 mm), eluted with an isocratic elution with 70% of CH_3_CN/H_2_O and a flow rate was 10 mL/min. This HPLC purification gave ventilatone A (**15**, t_R_ 6 min, 4.1 mg), ventilatone B (**12**, t_R_ 7 min, 10.5 mg) and ventilatone C (**16**, t_R_ 9 min, 5.0 mg).

### 3.7. Spectroscopic Data of a New Compound, Ventilatone C (**16**)

Yellow amorphous solid; [α]^25^_D_ +2.60 (*c* 0.25, CHCl_3_); UV (LC-UV, H_2_O:CH_3_CN, 30:70) λ_max_ 364.1, 288.0 and 233.5 nm; ESI-HRMS: *m/z* 299.0917 (M+H)^+^, calcd *m/z* 299.0919 for C_17_H_15_O_5_; ^1^H and ^13^C NMR spectroscopic data, see Table 2. 

### 3.8. Structure Elucidation of the Isolated Compounds

Structures of isolated compounds **7**–**13, 15** and **16** were elucidated by analysis of spectroscopic data (1D and 2D NMR, UV and ESI-HRMS spectroscopic techniques). ^1^H and ^13^C NMR spectra of compounds **7**–**13** and **15**, as well as 1D and 2D NMR of a new compound, ventilatone C (**16**), are in the Supplementary Materials. 

### 3.9. HPLC Fractionation of V. denticulata Extracts

100 mg of MeOH crude extract of bark of *V. denticulata* was dissolved in 60% MeOH and filtered through 0.45 µm filter before HPLC fractionation. A semi-preparative HPLC column, SunFire C_18_ (19 × 250 mm, 5.0 µm), was used. A gradient elution was performed using the following conditions: (i) linear gradient from 40% CH_3_CN (0.1% formic acid) in H_2_O (0.1% formic acid) to 100% CH_3_CN (0.1% formic acid) for 0–25 min, (ii) isocratic elution of 100% CH_3_CN (0.1% formic acid) for 5 min (at time of 25–30 min), (iii) a linear gradient from 100% CH_3_CN (0.1% formic acid) to 40% CH_3_CN (0.1% formic acid) in H_2_O (0.1% formic acid) for 4 min (at time of 30–34 min and (iv) an isocratic elution with 40% CH_3_CN (0.1% formic acid) in H_2_O (0.1% formic acid) for 6 min (at time of 34–40 min). The total run time was 40 min. The flow rate was 10 mL/min. The injection volume was 400 µL. The UV detector was set at wavelength of 200–400 nm, monitoring at 276 nm. This process yielded fractions FM1-FM7, which were obtained from HPLC fractionation of a MeOH crude extract of bark eluted at retention times (t_R_) of 1.0–6.0 min (FM1), 6.0–8.5 min (FM2), 8.5–12.0 min (FM3), 12.0–20.0 min (FM4), 20.0–28.0 min (FM5) and 28.0–34.0 min (FM6), respectively. Weights of fractions FM1-FM6 were 23.9 mg, 15.7 mg, 13.8 mg, 6.9 mg, 6.6 mg and 6.5 mg, respectively. The fractions FM1-FM6 were subsequently tested for antibacterial and antifungal activities and results are shown in Table 3. 

Fractionation of CH_2_Cl_2_ crude extract (100 mg) of bark of *V. denticulata* was carried out in the same manner as that of a MeOH crude extract, giving fractions FD1-FD10 with retention times (t_R_) of 1.0–5.5 min (FD1), 5.5–6.6 min (FD2), 6.6–7.1 min (FD3), 7.1–8.3 min (FD4), 8.3–9.5 min (FD5) and 9.5–13.0 min (FD6), respectively. Weights of fractions FD1-FD6 were 15.6 mg, 14.6 mg, 12.7 mg, 8.4 mg, 6.7 mg and 6.4 mg, respectively. Fractions FD1-FD6 were tested for antibacterial and antifungal activities and results are shown in Table 3. 

### 3.10. ESI-HRMS Analysis for the Identification of Compounds in HPLC Fractions

The fractions FM1-FM3 and FD1-FD4 from HPLC separation showing antibacterial and antifungal activities were subsequently analyzed by ESI-HRMS. The compounds in these fractions were tentatively identified by ESI-HRMS analysis based on the putative compounds listed in Table 1. The parameters setting were capillary exit of −110.0 V, skimmer of −35.0 V, hexapol RF of −110.0 V, hexapol 1 of −24 V, set corrector fill of 63 V, set pulsar pull of 405 V, set pulsar push of 405 V, set reflector of 1.3 kV, set flight tube of 9 kV, set detector TOF of 1.99 kV and scan range 100–1000 *m/z*. Results are displayed in Table 4.

### 3.11. In-Vitro Antibacterial and Antifungal Assays

#### 3.11.1. Preparation of Bacteria and Fungi for Bioassay

The bacterial strains used for an antibacterial assay were *P. aeruginosa* (TISTR No. 357), *E. coli* (TISTR No. 117), *S. enterica* serovar Typhimurium (TISTR No. 1470), *S. aureus* (TISTR No. 746) and *B. cereus* (TISTR No. 035). *C. albicans* (TISTR No. 5554) was the fungal strain used in an antifungal assay. All bacteria and *C. albicans* were purchased from the Thailand Institute of Scientific and Technological Research (Pathum Thani, Thailand). The stocks of bacteria and fungus were stored and kept in the freezer (−20 °C). Each of the bacterial and fungal strains was taken from the stocks and cultivated in a nutrient agar plate at temperature 37 °C for 24 h for bacteria and 48 h for *C. albicans*. A single colony of bacteria and *C. albicans* was selected and transferred into 10 mL 0.85% normal saline. Suspension of bacteria and fungi were adjusted to make the same turbidity with a 0.5 McFarland standard using spectrophotometer UV-Vis at wavelength of 600 nm [21].

#### 3.11.2. Disk Diffusion Method for Antibacterial and Antifungal Assays

The disk diffusion method was performed to screen antibacterial and antifungal activities. Amphotericin B was used as a standard drug for the antifungal test. Tetracycline and chloramphenicol were used as standard drugs for antibacterial assay. 20% ethanol in DMSO was used as a negative control. Sample and standard solutions were prepared at a concentration of 10 mg/ 100µL in the solvent (20% ethanol in DMSO). Each sample (10 µL) was impregnated in a sterile disk (Whatman antibiotic assay disk, diameter 6 mm) to give 1 mg/10 µL as a final concentration of each disk. However, compounds **12**, **13**, **15** and **16** were tested at a concentration of 0.5 mg/10 µL due to the limited amount of the compounds obtained. Bacterial or fungal solution (*ca* 10^8^ CFU/mL) was spread on a nutrient agar plate. Each disk was carefully placed on the plate containing bacteria or fungal solution and the plate was then incubated at 37 °C for 24 h. The diameter of a clear zone was measured as an indicator of inhibition toward bacteria or fungi [21]. Standard drugs for antibacterial activity were tetracycline and chloramphenicol and a standard drug for antifungal activity was amphotericin B; their activities are shown in Table 3. 

## 4. Conclusions

Nine antibacterial and antifungal natural products in the plant, *V. denticulata*, were isolated using UHPLC-ESI-QTOF-MS/MS-Based molecular networking guided isolation and dereplication. Five antimicrobial flavonoid glycosides (**7**–**11**), two benzisochromanquinone, ventilatones B (**12**) and A (**15**), a new naphthopyrone ventilatone C (**16**) and a triterpene lupeol (**13**) were isolated from *V. denticulata*. Dereplication technique also tentatively identified antimicrobial compounds in *V. denticulata*, including kaempferol, chrysoeriol, isopimpinellin, rhamnetin, luteolin, emodin, rhamnocitrin, ventilagodenin A, rhamnazin and mukurozidiol. The present work suggests that the molecular networking guided isolation and dereplication could assist the identification of antibacterial and antifungal agents in extracts of a plant. The presence of many antibacterial and antifungal compounds in the plant, *V. denticulata*, supports the traditional use of this plant as an herbal medicine for the treatment of wound infection.

Mass spectrometry-based molecular networking is a powerful dereplication strategy; it not only identifies known metabolites in complex mixtures but also suggests the presence of related analogues [6]. This work demonstrates that the molecular networking effectively assists the identification of antimicrobial compounds in plant extracts. 

## Figures and Tables

**Table 1:** Tentatively identified compounds in the bark and trunk of *V. denticulata* obtained from LC-MS/MS analysis. Compounds identified by Metlin Database [M], Human Metabolome Database [H] and standard compounds [S].

No.	t_R_ (min)	Compounds	Molecular Formula	Mass	Adduct Ions	Observed *m/z*	Calculated *m/z*	Δ (ppm)	Fragment Ions (*m/z*)	Found in Extracts ^a^
1	1.086	Unidentified	C_18_ H_3_N O_14_	487.1895	(M-H)^-^	486.1826	486.1828	0.50	341.1082, 179.0561, 144.0663, 119.0346, 101.0242	DT
2	1.091	Unidentified	C_37_H_36_N_2_ O_11_	684.2316	(M-H)^-^	683.2244	683.2246	0.40	341.1086, 179.0556, 119.0346	MB, MT
3	1.357	2′-Methoxy-3-(2,4-dihydroxyphenyl)-1,2-propanediol 4′-glucoside [M, H]	C_16_ H_24_ O_9_	360.1420	(M+Na)^+^	383.1313	383.1313	−0.01	306.9908, 248.9974, 207.0666, 185.0403, 102.0900	MB, MT
4	1.371	Kaempferol-3-rhamninoside [M]	C_33_ H_40_ O_19_	740.2162	(M+H)^+^	741.2233	741.2237	0.50	595.1677, 449.1072, 346.0867, 287.0557, 147.0649	MB
					(M-H)^-^	739.2080	739.2091	1.49	285.0396, 255.0315	MB, MT
5	1.405	Rhamnetin 3-rhamninoside [M]	C_34_ H_42_ O_20_	770.2269	(M+H)^+^	771.2343	771.2342	−0.09	479.1186, 317.0657, 239.0928, 163.0602, 147.0653, 129.0548	MB, MT
					(M-H)^-^	769.2192	769.2197	0.66	315.0505, 299.0186	MB, MT, DT
6	1.414	1,2,10-Trihydroxydihydro-trans-linalyl oxide 7-*O*-β-D-glucopyranoside [M, H]	C_16_ H_30_ O_10_	382.1841	(M+Na)^+^	405.1733	405.1731	−0.54	355, 0125, 273.1298, 129.0543	MB
					(M-H)^-^	381.1762	381.1766	1.15	322.0691, 249.1343, 205.3362, 161.0450, 113.0235, 101.0243	MB, DB
7	1.493	Rhamnocitrin 3- rhamninoside [M]	C_34_ H_42_ O_19_	754.2320	(M+H)^+^	755.2394	755.2393	−0.19	463.1233, 301.0709, 163.0600, 147.0651, 129.0543	MB, MT, DT
					(M-H)^-^	753.2239	753.2248	1.12	557.2233, 299.0554, 283.0236	MB, MT, DT
8	1.499	Unidentified	C_27_ H_34_ N_7_ O_21_	792.1803	(M+2H)^+^^2^	397.0973	397.0977	0.97	647.1279, 575.1043, 545.1010, 501.0683, 399.0395, 339.0179, 201.0041, 121.0495	MB, MT
9	1.535	Furocoumarinic acid glucoside [M, H]	C_17_H_18_ O_9_	366.0955	(M+H)^+^	367.1024	367.1024	−0.21	349.0928, 331.0806, 307.0803, 289.0703, 275.0556, 263.0559, 217.0494, 161.0594	DB
					(M-H)^-^	365.0871	365.0878	1.96	350.0639, 306.0746, 289.0707, 274.0482, 246.0522, 161.0181	DB
10	1.540	Unidentified	C_27_ H_50_ Cl_2_ N_9_ O_8_ S	762.2601	(M+Na)^+^	785.2493	785.2493	−0.03	493.1342, 331.0815, 147.0664	DB
11	1.547	Unidentified	C_29_ H_30_ N_16_ O_11_	778.2272	(M+2H)^+^^2^	412.1027	412.1032	1.32	677.1394, 575.1073, 429.0485, 369.0279, 266.0451, 201.0073, 129.0543	MB
12	1.558	3,3′,4′-Trihydroxyflavone 3-*O*-[α-L-rhamnopyranosyl-(1→2)[*α*-L-rhamnopyranosyl-(1→6)]-*β*-D-glucopyranoside] [M, H]	C_33_ H_40_ O_18_	724.2206	(M+CH_3_COO)^-^	783.2345	783.2353	1.06	453.1600, 329.0657, 314.0425, 145.0503, 101.0246	MB, MT, DB, DT
13	1.564	5,7,8-Trihydroxyflavanone 7-glucoside [M, H]	C_21_H_22_ O_10_	434.1212	(M-H)^-^	433.1138	433.1140	0.59	313.0719, 271.0556, 270.0528, 231.0611, 139.0402	MB, DT
14	1.575	Rhamnazin 3-rhamninoside [M]	C_35_ H_44_ O_20_	784.2430	(M+H)^+^	785.2503	785.2503	0.00	493.1341, 331.0814, 163.0599, 147. 0653, 129.054	MB, MT, DB, DT
					(M-H)^-^	783.2349	783.2348	−0.13	537.1992, 453.1584, 329.0664, 234.1049, 145.0490	MB, MT, DB, DT
15	1.626	Astragalin [M, H]	C_21_ H_20_ O_11_	448.1009	(M+H)^+^	449.1081	449.1078	−0.58	317.0661, 287.0553, 269.0444, 195.0657	MB, MT, DT
16	1.629	Unidentified	C_23_ H_30_ N_7_ O_8_	532.2155	(M+Na)^+^	555.2043	555.2048	0.88	381.1307, 286.0742, 207.0619, 147.0433	MB
17	1.634	Kaempferol 5-glucoside [M, H]	C_21_ H_20_ O_11_	448.0996	(M+HCOO)^-^	493.0979	493.0988	1.76	346.8297, 327.0481, 298.0487, 285.0402, 240.0460	MB, MT, DT
18	1.635	Naringenin 4′-*O*-glucuronide [M, H]	C_21_ H_20_ O_11_	448.1002	(M+Na)^+^	471.0895	471.0898	0.57	339.0471, 309.0368, 294.0188, 249.1094, 161.9958	MB, MT
19	1.708	Aloesol [M, H]	C_13_ H_14_ O_4_	234.0891	(M+H)^+^	235.0964	235.0965	0.45	217.0860, 191.0705, 163.0754, 151.0385, 135.0804, 107.0847	MB, MT, DT
					(M-H)^-^	233.0818	233.1819	0.60	189.0552, 161.0593, 149.0251	MB, MT, DB
20	1.765	Zingerone glucoside [M, H]	C_17_ H_24_ O_8_	356.1468	(M+Na)^+^	379.1361	379.1363	0.63	323.9212, 278.3414, 235. 8741, 217.0847, 111.0775	MB, DB
21	1.810	Unidentified	C_33_ H_46_ N_4_ O_6_	594.3419	(M+H)^+^	595.3492	595.349	−0.35	577.3542, 536.2739, 173.1640, 120.0805	MB
22	1.852	Unidentified	C_21_ H_28_ O_8_	408.1771	(M+Na)^+^	431.1674	431.1676	0.53	317.1031, 275.0908, 205.0465	DB
23	1.909	Xanthotoxol glucoside [M, H]	C_17_ H_16_ O_9_	364.0795	(M+H)^+^	365.0866	365.0867	0.36	305.0661, 291.0851, 277.0713, 259.0606, 215.0704, 132.0900	DB
					(M-H)^-^	363.0716	363.0722	1.51	304.0583, 287.0556, 272.0320, 261.0404, 244.0375, 228.0435, 201.0195	DB
24	2.000	Unidentified	C_24_ H_18_ N_8_ O_4_	482.1448	(M+Na)^+^	505.1338	505.1343	0.96	419.1317, 343.1048, 257.0809, 127.0393	MB
25	2.006	Isoliquiritin [M, H]	C_21_ H_22_ O_9_	418.1261	(M+H)^+^	419.1334	419.1337	0.71	335.0877, 257.0804, 239.0703, 191.0702, 127.0390	MB
					(M-H)^-^	417.1184	417.1191	1.66	297.0764, 255.0643	MB, MT, DB
26	2.042	6”-*O*-Acetyldaidzin [M, H]	C_23_ H_22_ O_10_	458.1209	(M+HCOO)^-^	503.1192	503.1195	0.67	418.1190, 297.0765, 255.0690	MB
27	2.190	Glucoemodin [M, H]	C_21_ H_20_ O_10_	432.1051	(M-H)^-^	431.0979	431.0984	1.07	344.8229, 311.0557, 269.0448, 227.1067	MB, MT, DT
28	2.282	Kievitol [M, H]	C_20_ H_22_ O_7_	374.1356	(M-H)^-^	373.1284	373.1293	2.29	359.0953, 246.0522, 193.0504, 179.0714, 164.0475, 149.0600, 134.0368	DB
29	2.291	Wharangin [M, H]	C_17_ H_12_ O_8_	344.0536	(M+H)^+^	345.0608	345.0605	−0.74	303.0497, 327.0487, 299.0544, 275.0543, 261.0401, 195.0290	DB, DT
					(M-H)^-^	343.0454	343.0459	1.69	330.0381, 301.0348, 287.0196, 273.0040, 158.0608	DB
30	2.314	4″-Methyl-6″-(3,4-dihydroxy-E-cinnamoyl)isoorientin [M, H]	C_31_ H_28_ O_14_	624.1474	(M-H)^-^	623.1400	623.1406	1.02	517.8187, 458.3673, 375.3759, 298.0471, 295.0808, 285.0416, 241.0516	MB
31	2.376	Chrysoeriol [M, H]	C_16_ H_12_ O_6_	300.0640	(M+H)^+^	301.0712	301.0707	−1.80	273.0397, 260.0310, 255.0651, 245.0442, 227.0698	DB
					(M-H)^-^	299.0561	299.0561	0.00	270.0168, 258.0166, 255.0661, 240.0428, 227.0346, 214.0269, 151.0033	DB
32	2.377	6”-Malonylcosmosiin [M, H]	C_24_ H_22_ O_13_	518.1048	(M-H)^-^	517.0975	517.0988	2.44	473.1078, 432.1734, 385.1729, 269.0452, 225.0402	MB
33	2.382	Cicerin 7-(6-malonylglucoside) [M, H]	C_26_ H_26_ O_15_	578.1269	(M+H)^+^	579.1341	579.1344	0.64	437.0247, 342.9891, 331.0819, 147.0531, 127.0390	MB
34	2.416	Unidentified	C_40_ H_38_ N O_5_ S	644.2467	(M+Na)^+^	667.2356	667.2363	1.04	553.2780, 425.0864, 329.1411, 129.0528	MB, DT
35	2.445	Quercetin [M, H]	C_15_ H_10_ O_7_	302.0422	(M+H)^+^	303.0494	303.0499	1.86	276.8345, 240.8436, 229.0471, 195.0268, 182.9751, 139.8692	MB
					(M-H)^-^	301.0351	301.0354	0.76	273.0382, 229.0518, 178.9980, 151.0032, 121.0300, 107.0132	MB
36	2.552	Unidentified	C_18_ H_40_ N_5_ O_18_	614.2359	(M+Na)^+^	637.2247	637.2261	2.19	537.1811, 410.0280, 339.1044, 145.0475, 110.0979	MB, DT
37	2.563	Emodinanthranol [M, H]	C_15_ H_12_ O_4_	256.0738	(M+H)^+^	257.0810	257.0808	−0.66	242.0590, 217.0500, 214.0612, 198.9313, 145.0656, 101.0594	DB
					(M-H)^-^	255.0658	255.0663	1.84	213.0555, 187.0768, 183.0814	DB
38	2.753	*α*-Hydrojuglone 4-*O*-*β*-D-glucoside [H]	C_13_ H_18_ O_5_	338.0995	(M-H)^-^	337.0922	337.0929	1.98	250.0844, 221.081, 163.0765	MB, DB, DT
39	3.039	Unidentified	C_13_ H_20_N_3_ O_8_ S	378.0958	(M+H)^+^	379.1025	379.1044	4.86	319.0809, 291.0861, 202.0630, 111.0421	DB
40	3.087	Unidentified	C_17_ H_18_ O_8_	350.1003	(M+H)^+^	351.1075	351.1074	−0.19	333.0949, 301.0702, 276.0630, 259.0604, 215.0700	DB
					(M-H)^-^	349.0924	349.0929	1.35	334.0694, 319.0457, 291.0506, 219.0304	DB
41	3.131	Unidentified	C_28_ H_24_ O_12_	552.1264	(M-2H)^-^^2^	275.0558	275.0561	1.17	338.0072, 262.0703, 232.0368, 218.0236, 188.0462	DB
42	3.233	Isopimpinellin [M, H]	C_13_ H_10_ O_5_	246.0522	(M+CH_3_COO)^-^	305.0660	305.0667	2.34	245.0447, 201.0512, 173.0585, 129.0714	DB
43	3.274	Kaempferol [M, H]	C_15_ H_10_ O_6_	286.0480	(M+H)^+^	287.0554	287.0550	−1.39	227.8855, 165.0174, 153.0172, 121.0271	MB, MT
					(M-H)^-^	285.0401	285.0405	1.37	257.0426, 241.0493, 229.0487, 211.0396, 151.0029	MB, MT, DB
44	3.291	Coriandrone C [M, H]	C_13_ H_10_ O_5_	246.0536	(M+H)^+^	247.0609	247.0601	−3.39	229.0499, 219.0262, 201.0552, 173.0586, 158.0695, 137.1239	DB
45	3.364	Eriodictyol [M, H]	C_15_ H_12_ O_6_	288.0631	(M-H)^-^	289.0704	289.0707	0.84	271.0589, 259.0603, 257.0465, 231.0641, 229.0488, 173.0582	DB
					(M-H)^-^	287.0558	287.0561	0.93	259.0604, 243.0653, 201.0582, 177.0550, 151.0041, 125.0243	MB, MT, DT
46	3.583	Coumesterol [M, H]	C_15_ H_8_ O_5_	268.0373	(M+H)^+^	269.0446	269.0444	−0.46	243.1493, 241.0487, 213.0553, 185.0602, 157.0644	MB, MT
47	3.594	Citreorosein [M, H]	C_15_ H_10_ O_6_	286.0482	(M+H)^+^	287.0555	287.0550	−1.65	269.0447, 213.0536, 185.0593	MB, MT, DT
					(M-H)^-^	285.0404	285.0405	0.35	241.0503, 172.9762	MB, MT, DB, DT
48	3.608	Physcion [M]	C_16_ H_12_ O_5_	284.0688	(M+H)^+^	285.0761	285.0757	−1.4	257.0808, 243.0644, 239.0696, 229.0496, 211.0750	DB
					(M-H)^-^	283.0612	283.0612	−0.07	255.0650, 241.0503, 239.0703, 227.0345, 224.0477	DB
49	3.698	*R*-Angolensin [M]	C_16_ H_16_ O_4_	272.1051	(M+H)^+^	273.1124	273.1121	−0.81	255.1016, 231.1015, 227.1068, 189.0915, 174.0667, 111.8671	DB
50	3.894	(±)-Sphaerosin [M, H]	C_17_ H_18_ O_5_	302.1153	(M+H)^+^	303.1225	303.1227	0.80	285.1117, 261.1129, 257.1174, 219.1029, 204.0783, 163.0361	MB, DB
51	3.919	Unidentified	C_34_ H_36_ O_10_	604.2310	(M+Na)^+^	627.2204	627.2201	−0.58	325.1052	DB
52	3.926	3-Hydroxyphloretin [M, H]	C_15_ H_14_ O_6_	290.0785	(M+HCOO)^-^	335.0767	335.0772	1.67	268.0917, 259.0604, 248.0686, 220.0728, 205.0504, 147.0429	MB, DB
53	3.996	3′,7-Dihydroxy-4′,8-dimethoxyisoflavone [H]	C_17_ H_14_ O_6_	314.0785	(M-H)^-^	313.0712	313.0718	1.82	300.0246, 269.0808, 254.0571, 239.0326	MB, DB
54	4.062	Unidentified	C_19_ H_22_ O_10_	410.1214	(M+Na)^+^	433.1106	433.1105	−0.21	401.0840, 369.0571, 341.0618, 250.5698	MB
55	4.064	Unidentified	C_37_ H_32_ N_3_ O_15_	758.1834	(M-H)^-^	757.1760	757.1761	0.13	713.1893, 458.1202, 410.6138, 373.7386, 299.7235, 254.0514, 191.1313	MB, MT
56	4.075	Unidentified	C_25_ H_30_ N_8_ O_7_	650.1400	(M+H)^+^	651.1473	651.1472	−0.03	337.0683	DB
57	4.132	5,6,7,8-Tetrahydroxy-3′,4′-dimethoxyflavone [M, H]	C_17_ H_14_ O_8_	346.0681	(M-H)^-^	345.0610	345.0616	1.85	331.0413, 298.0119, 270.0171, 242.0246	MB
58	4.134	5-Hydroxy-4′,7,8-trimethoxyflavone [M, H]	C_18_ H_16_ O_6_	328.0939	(M-H)^-^	327.0867	327.0874	2.06	312.0620, 286.0477, 271.0240, 268.0732, 253.0500, 225.0558	DB
59	4.178	Unidentified	C_12_ H_8_ N_5_ O_6_ S	350.0196	(M+H)^+^	351.0265	351.0268	0.77	297.3586, 261.9442, 245.8488, 222.0035, 181.0472, 135.0783	MB
60	4.203	Unidentified	C_34_ H_24_ O_12_	624.1270	(M+Na)^+^	647.1162	647.1160	−0.38	335.053	DB
61	4.251	Aloe emodin w-acetate [M, H]; or Ventilatone A (isolation)	C_17_ H_12_ O_6_	312.0636	(M+H)^+^	313.0706	313.0707	0.29	285.0759, 271.0604, 243.0659, 215.0685, 167.8890	MB, DB
					(M-H)^-^	311.0559	311.0561	0.72	297.0393, 269.0438, 268.0373, 253.0140, 224.0472	DB
62	4.532	Cartorimine [M, H]	C_15_ H_14_ O_6_	290.0794	(M+Na)^+^	313.0686	313.0683	−1.23	276.9105, 212.8751, 123.1149	MB, MT
					(M-H)^-^	289.0712	289.01718	1.81	273.0402, 259.0239, 245.0457, 201.0550,	MB, MT, DB
63	4.619	Rhamnetin [M]	C_16_ H_12_ O_7_	316.0581	(M+H)^+^	317.0654	317.0654	0.56	271.0590, 243.0679, 167.0342, 121.0279	MB
					(M-H)^-^	315.0505	315.0510	1.57	300.0261, 166.0221, 121.0293, 112.9849	MB, DB
64	4.721	Luteolin [M, H]	C_15_ H_10_ O_6_	286.0473	(M-H)^-^	285.0401	285.04005	1.34	270.0163, 257.0450, 241.0499, 213.0526, 151.9236	MB, MT, DB, DT
65	4.752	Unidentified	C_18_ H_14_ O_7_	342.0744	(M+Na)^+^	365.0636	365.0632	−1.14	321.0373, 305.0419, 156.0637	DB
66	4.874	5,4′-Dihydroxy-3,3′-dimethoxy-6:7-methylenedioxyflavone [M, H]	C_18_H_14_O_8_	358.0688	(M+Na)^+^	381.0579	381.0581	0.39	349.0312, 333.4380, 328.4933, 273.3009, 243.5325, 189.0203	DB
67	4.999	1,3,5-Trihydroxy-6,7-dimethoxy-2-methylantraquinone [H]	C_16_ H_10_ O_7_	330.0734	(M-H)^-^	329.0661	329.0667	1.74	314.0427, 299.0207, 288.0280, 285.077, 273.0031, 270.0525, 258.0168	MB, TB, DB, DT
68	5.007	Ventilagodenin A; or 5-De-*O*-methyltoddanol [M, H]	C_15_ H_16_ O_5_	276.1000	(M+H)^+^	277.1074	277.1071	−1.21	259.0957, 244.0731, 235.0973, 199.0748, 171.0804	MB, TB, DB, DT
					(M-H)^-^	275.0922	275.0925	1.14	259.0609, 245.0447, 231.0661, 192.6885, 175.0355	MB, TB, DB
69	5.097	Unidentified	C_18_ H_10_ N O_4_	304.0612	(M+Na)^+^	327.0504	327.0502	−0.46	287.0555, 259.0604, 255.0288, 245.0422, 167.0345	DB
70	5.129	Rhamnalpinogenin [M, H]; or Ventilatone B (isolation)	C_17_ H_12_ O_7_	328.0589	(M+H)^+^	329.0659	329.0656	−1.09	311.0551, 287.0551, 259.0607, 167.0345	MB, TB, DB, DT
					(M-H)^-^	327.0508	327.0510	0.63	312.0273, 284.0326, 269.0092, 256.0378, 185.0239	DB
71	5.135	Unidentified	C_12_ H_8_ N_5_ O_7_ S	366.0142	(M+H)^+^	367.0212	367.0217	1.49	352.3162, 309.0637, 277.0991, 235.8736, 186.9023, 123.1163	MB
72	5.152	3,5,7-Trihydroxy-4′,6-dimethoxyflavanone [M, H]	C_17_ H_16_ O_7_	332.0891	(M+HCOO)^-^	377.0873	377.0878	1.30	317.0660, 306.0738, 259.0245, 174.9557, 130.9658	MB, DB
73	5.265	Mukurozidiol (M, H)	C_17_ H_18_ O_7_	334.1051	(M+H)^+^	335.1123	335.1125	0.77	303.0866, 285.0752, 275.0914, 261.0750, 245.0448, 233.0425	MB, DB, DT
					(M+HCOO)^-^	379.1026	379.1035	2.23	308.0893, 305.0640, 277.0688, 262.0477, 174.9575	MB, DB
74	5.288	Unidentified	C_13_ H_20_ N_3_ O_8_ S	378.0957	(M+H)^+^	379.1026	379.1044	4.68	364.0528, 291.0863, 215.0331, 115.0550	MB
75	5.296	Unidentified	C_19_ H_22_ O_10_	410.1214	(M+Na)^+^	433.1106	433.1105	−0.13	373.0897, 342.0707, 327.0475	MB
76	5.420	Genistin [M, H]	C_21_ H_20_ O_10_	432.1036	(M+H)^+^	433.1109	433.1129	4.76	401.0843, 373.0894, 369.0579, 342.0711, 327.0470	DB
77	5.463	6′-Hydroxyangolensin [M, H]	C_16_ H_16_ O_5_	288.1000	(M+H)^+^	289.1073	289.1071	−0.92	271.0967, 247.0966, 243.1013, 229.0856, 205.0864	DB
					(M-H)^-^	287.0920	287.0925	1.70	269.0821, 254.0605, 245.0823, 203.0702	DB
78	5.578	(*S*)-Rutaretin [M,H]	C_14_ H_14_ O_5_	262.0835	(M-H)^-^	261.0761	261.0768	2.71	246.0527, 231.0291, 218.0561, 203.0352	DB
79	5.611	Unidentified	C_35_ H_30_ O_11_	626.1773	(M+HCOO)^-^	671.1753	671.177	2.60	509.1242, 416.1098, 254.0577	TB, TD
80	5.650	Pratenol A [M,H]	C_14_ H_12_ O_5_	260.0687	(M+H)^+^	261.0759	261.0757	−0.49	243.0656, 215.0705, 200.0470, 187.0749, 159.0439	DB
81	5.743	Gingerenone C [M, H]	C_20_ H_22_ O_4_	326.1521	(M+H)^+^	327.1592	327.1591	−0.46	203.1049, 171.0802, 151.0758, 148.1110, 137.0600	DB
82	5.848	Unidentified	C_53_ H_26_ N_3_ O_2_	736.2027	(M+Na)^+^	759.1920	759.1917	−0.37	664.0398, 504.1286, 418.1196, 299.0856, 256.0729	MB
83	6.222	Afzelechin [M, H]	C_15_ H_14_ O_5_	274.0841	(M-H)^-^	273.0768	273.0768	0.29	229.0501, 202.026	MB, DB
84	6.342	Ducunolide E [M, H]	C_26_ H_28_ O_9_	484.1724	(M-H)^-^	483.1650	483.1661	2.19	468.1412, 439.1389, 424.1156, 409.0887	DB
85	6.472	Rhamnocitrin [M]	C_16_ H_12_ O_6_	300.0637	(M+H)^+^	301.0711	301.0707	−1.35	286.0458, 179.03331, 167.0344, 121.0286	MB, DB, DT
					(M-H)^-^	299.0556	299.0561	1.78	284.0310, 271.0605, 240.0420, 178.0257, 165.0189	MB, DB, DT
86	6.607	7-Hydroxy-3,4′,8-trimethoxyflavone [M, H]	C_18_ H_16_ O_6_	328.0949	(M+H)^+^	329.1023	329.1020	−1.04	314.0786, 313.0702, 285.0766, 198.0922, 121.1025	DB
87	6.698	Acerosin [M, H]	C_18_ H_16_ O_8_	360.0834	(M-H)^-^	359.0761	359.0772	3.11	344.0538, 297.0054, 269.0084, 171.2585	MB, DB
88	6.732	Unidentified	C_13_ H_13_ N_6_ O_7_	365.0841	(M+2Na)^+^^2^	205.5309	205.5315	2.91	320.7446, 254.9948, 205.1755, 155.0088, 141. 5110, 112.4964	MB
89	6.766	Alfalone [M, H]	C_17_ H_14_ O_5_	298.0841	(M+H)^+^	299.0916	299.0914	−0.54	271.3851, 213.8909, 189.0528, 112.7128	DB, DT
90	6.775	Rhamnazin [M, H]	C_17_ H_14_ O_7_	330.0743	(M+H)^+^	331.0816	331.0812	−1.22	316.0577, 299.0542, 288,0634, 179.0327, 167.0338	MB, DB
					(M-H)^-^	329.0664	329.0667	0.74	315.0457, 314.0424, 286.0478, 254.0217, 241.051, 170.0353	MB, DB
91	6.924	Xanthoxyletin [M, H]	C_15_ H_14_ O_4_	258.0894	(M+H)^+^	259.0967	259.0965	−0.73	244.0734, 241.0863, 226.0628, 217.0862, 213.0906, 195.0799, 167.0879	DB
92	6.981	Barpisoflavone A [M, H]	C_16_ H_12_ O_6_	300.0636	(M+H)^+^	301.0708	301.0707	−0.43	287.0570, 269.0441, 236.9047, 185.0603, 127.0056	MB, TB, DT
					(M-H)^-^	299.0560	299.0561	0.24	267.0297, 240.0422, 212.0476	MB, TB, DB
93	7.015	(+)-(*R*)-Ventilagolin [S]	C_17_ H_16_ O_7_	332.0897	(M+H)^+^	333.0971	333.0969	0.60	318.0736, 301.0710, 276.0630, 259.0606, 213.0544, 185.0596	MB, DB, DT
					(M+HCOO)^-^	377.0873	377.0878	1.30	317.066, 306.0738, 303.0506, 259.0245, 174.9557	MB, DB, DT
94	7.123	Caryatin [M, H]	C_17_ H_14_ O_7_	330.0741	(M+H)^+^	331.0813	331.0812	−0.12	299.0551, 276.0625, 259.0611, 211.3641, 167.0181	MB, DB
					(M-H)^-^	329.0660	329.0667	2.00	314.0423, 299.0194, 286.0488, 271.0240, 165.0184	MB, MT, DB, DT
95	7.349	Kanzonol O [M, H]	C_22_ H_22_ O_6_	382.1418	(M+Na)^+^	405.1310	405.1309	−0.29	335.0526, 270.0508, 143.0333	DB
96	7.548	Unidentified	C_12_ H_24_ Cl_2_ N_2_ O_8_ S	426.0635	(M+Na)^+^	449.0527	449.0523	−0.91	408.2483, 388.7627, 287.1038	MB
97	7.887	Unidentified	C_34_ H_30_ N_3_ O_11_	656.1874	(M-H)^-^	655.1800	655.1808	1.18	557.9872, 254.0580	MB, MT
98	8.003	Unidentified	C_33_ H_28_ N_3_ O_11_	642.1727	(M-H)^-^	641.1655	641.1651	−0.57	509.1224, 491.1100, 254.0579	MB, MT
99	8.120	Unidentified	C_16_ H_11_ N O	233.0844	(M+Na)^+^	256.0734	256.0733	−0.36	240.0926, 210.0659, 1821.0653, 157.0646, 140.9164	MB
100	8.507	Dihydromorelloflavone [M, H]	C_30_ H_22_ O_11_	558.1161	(M+H)^+^	559.1236	559.1235	−0.15	541.1141, 523.0991, 517.1109, 513.1141, 499.1013, 313.0354, 257.0795	DB
					(M-H)^-^	557.1085	557.1089	0.80	539.0915, 526.0836, 359.8609, 155.1055	DB
101	8.938	Emodin [M, H, S]	C_15_ H_10_ O_5_	270.0528	(M+H)^+^	271.0601	271.0601	−0.16	229.0509, 225.0560, 201.0539, 197.0590, 140.0222	MB, MT, DB, DT
					(M-H)^-^	269.0452	269.0455	1.33	241.0511, 225.0562, 210.0316, 195.0415, 135.0911	MB, MT, DB, DT
102	9.187	Formononetin [M, H]	C_16_ H_12_ O_4_	268.0740	(M+H)^+^	269.0813	269.0808	−1.60	254.0572, 239.0708, 226.0618, 151.0543	DB
103	9.383	6*α*-Hydroxymaackiain [M, H]	C_16_ H_12_ O_6_	300.0637	(M+H)^+^	301.0709	301.0707	−0.76	255.0638, 117.0696	MB, DB
104	9.986	Unidentified	C_22_ H_18_N_7_O_3_	428.1473	(M+Na)^+^	451.1363	451.1363	−0.01	319.0570, 292.0353, 133.0864	DB
105	9.991	Artonin L [M, H]	C_22_ H_20_ O_7_	396.1213	(M+H)^+^	397.1283	397.1282	−0.36	379.1160, 366.1054, 337.1045, 327.1201, 295.0939, 287.0557	DB
106	10.699	Muscomin [M, H]	C_18_ H_18_ O_7_	346.1053	(M+H)^+^	347.1125	347.1125	−0.01	332.0896, 315.0864, 290.0781, 273.0764, 227.0696	DB
107	10.824	Unidentified	C_15_ H_11_ O_4_	255.0658	(M+H)^+^	256.0731	256.073	−0.41	241.0502, 238.0625, 210.0683, 198.9302, 182.0727	MB, MT, DB
108	11.423	2′,3,5-Trihydroxy-5′,7-dimethoxyflavanone [M, H]	C_19_ H_20_ O_9_	332.0885	(M+CH_3_COO)^-^	391.1024	391.1035	2.64	317.0658, 302.0387, 242.6421, 209.8790, 130.2329	DB
109	11.796	Palmidin A [M, H]	C_30_ H_22_ O_8_	510.1312	(M+H)^+^	511.1387	511.1387	0.16	256.0733, 133.0854	MB, MT, DB, DT
					(M-H)^-^	509.1238	509.1242	0.80	254.0583	MB, MT, DB, DT
110	12.237	1,3,5,8-Tetrahydroxy-6-methoxy-2-	C_16_ H_12_ O_7_	316.0585	(M+H)^+^	317.0658	317.0656	−0.56	299.0575, 254.8649, 193.0125, 135.1168, 127.0534	MB, DB
		methylanthraquinone [M, H]			(M-H)^-^	315.0505	315.0510	1.57	300.0261, 272.0305, 216.9344, 163.1615, 112.9849	MB
111	12.742	Khelmarin D [M, H]	C_28_ H_24_ O_8_	488.1460	(M+CH_3_COO)^-^	547.1599	547.1610	2.00	457.0900	DB
112	12.798	Amentoflavone [M, H]	C_30_ H_18_ O_10_	538.0889	(M-H)^-^	537.0814	537.0827	2.44	469.0870, 400.8285, 333.5261, 173.9422, 107.5508	MB, MT, DB
113	12.837	Isophysalin G [M, H]	C_28_ H_30_ O_10_	526.1860	(M+Na)^+^	549.1752	549.1731	−3.87	517.1481, 475.1364, 246.0893	DB
114	13.326	Yuccaol C [M, H]	C_30_ H_22_ O_10_	542.1201	(M-H)^-^	541.1126	541.1140	2.6	523.0998, 511.0683, 493.0539, 308.0347, 231.1206	DB
115	13.632	Ephedrannin A [M, H]	C_30_ H_20_ O_11_	556.0997	(M+CH_3_COO)^-^	615.1134	615.1144	1.59	299.0208, 289.0709	DB
116	14.355	Unidentified	C_29_ H _22_ N_3_ O_7_	524.1462	(M-H)^-^	523.1387	523.1385	−0.47	254.0580	MB
117	14.718	Unidentified	C_16_ H_13_ O_4_	269.0814	(M+H)^+^	270.0885	270.0887	0.78	227.07006, 179.0025, 151.9915, 105.0345	MB
118	14.748	Palmidin B [M, H]	C_30_ H_22_ O_7_	494.1349	(M-H)^-^	493.1279	493.1293	2.74	386.1758, 340.4709, 254.0581, 224.0460, 213.0023, 161.4482	DB, DT
119	16.060	Murrayazolinine [M, H]	C_23_ H_27_ N O_2_	349.2042	(M+NH_4_)^+^	367.2390	367.2380	−2.81	323.2308, 268.2613, 172.1157, 156.1387, 116.0538	MB, MT, DB. DT
120	16.060	Unidentified	C_32_ H_28_ N_2_ S_3_	536.1416	(M+Na)^+^	559.1318	559.1307	−1.94	521.0807, 466.7954, 409.8348, 401.2433	MB
121	17.810	Rheidin B [M, H]	C_30_ H_20_ O_8_	508.1146	(M-H)^-^	507.1074	507.1085	2.24	479.1105, 304.9145	MB, MT, DT
122	18.371	Copalic acid [M, H]	C_20_ H_32_ O_2_	304.2407	(M+H)^+^	305.2479	305.2475	−1.40	259.2411, 149.1327, 137.1326, 123.1165, 109.1010	MB
123	20.707	γ-Pinacene [M, H]	C_20_ H_32_	272.2506	(M+H)^+^	273.2578	273.2577	−0.45	231.2105, 175.1484, 163.1482, 149.1327, 135.1169, 121.1014, 109.1013, 107.0856	DB
124	20.750	Pipericine [M, H]	C_22_ H_41_ N O	335.3190	(M+H)^+^	336.3264	336.3261	−0.90	240.2341, 184.1702, 142.1230, 170.1534, 100.0761	MB, MT, DB, DT
125	22.976	Araliacerebroside [M, H]	C_40_ H_77_ N O_10_	731.5543	(M+Na)^+^	754.5435	754.5440	0.63	ND	MB, MT, DT
					(M-H)^-^	730.5462	730.5475	1.71	568.4923, 416.3272, 326.2700, 271.2258, 179.0551, 131.0328, 119.0354	MB, MT
126	23.282	Unidentified	C_26_ H_51_ N_13_	545.4386	(M+Na)^+^	568.4274	568.4283	1.52	476.3663, 371.2275, 250.1754, 185.1303, 133.0845	MB
127	24.262	Unidentified	C_26_ H_45_ N_4_	413.3639	(M+H)^+^	414.3710	414.3717	1.59	112.0989	MB
128	24.301	Unidentified	C_26_ H_49_ N O	391.3819	(M+H)^+^	392.3893	392.3887	−1.51	282.2781, 198.1852, 156.1385, 130.1590	MB, MT
129	24.466	Unidentified	C_36_ H_38_ N_4_ O_5_	606.2843	(M+H)^+^	607.2917	607.2915	−0.35	547.27	MB
130	24.500	Clerosterol 3-glucoside [M, H]	C_35_ H_58_ O_6_	574.4219	(M+CH_3_COO)^-^	633.4359	633.4372	2.00	559.3987, 541.3890, 383.3517, 175.0401, 133.0300	DB
131	24.755	Unidentified	C_24_ H_25_ N_9_ O_2_ S_2_	535.1579	(M+H)^+^	536.1658	536.1645	−2.30	503.1070, 415.0364, 341.0176, 221.0841, 147.0655	MB, TB
132	24.913	Unidentified	C_37_ H_38_ N_5_ O_2_	584.3020	(M+Na)^+^	607.2911	607.2918	1.17	547.2713, 460.2258, 367.0213, 280.2360, 167.1421, 107.0840	MB
133	25.117	AS 1-5 [M, H]	C_40_ H_77_ N O_9_	715.5597	(M+Na)^+^	738.5489	738.5489	0.15	ND	MB, MT, DT
					(M+HCOO)^-^	760.5560	760.5580	2.69	655.7664, 552.4965, 534.4872, 299.4631, 179.0584, 101.0237	MB, MT
134	25.474	3-Dehydroteasterone [M, H]	C_28_ H_46_ O_4_	446.3401	(M+Na)^+^	469.3293	469.3288	-0.93	385.1727, 329.1716, 189.0170, 171.0054, 113.1314	MB, MT, DB, DT
135	25.552	Unidentified	C_42_ H_74_ N_6_ O_10_	822.5471	(M-H)^+^	821.5396	821.5394	-0.32	775.5344, 613.0880, 523.3704, 339.4486, 277.2172, 261.1697, 175.6021, 103.9958	MB
136	25.644	Unidentified	C_29_ H_41_ N_2_ O_2_ S_5_	609.1765	(M+H)^+^	610.1843	610.1844	0.30	489.0548, 355.0700, 281.0509, 221.0844, 147.0659	MB
137	26.561	Secasterone [M, H]	C_28_ H_46_ O_4_	446.3395	(M+Na)^+^	469.3286	469.3286	0.44	329.1732, 284.1760, 268.0679, 109.1008	MB, MT
138	26.727	Unidentified	C_42_ H_76_ N_6_ O_10_	824.5628	(M-H)^-^	823.5555	823.5550	−0.60	778.5514, 713.2510, 657.5735, 579.3840, 513.3079, 456.2245, 388.2563, 277.2178	MB
139	26.799	Unidentified	C_36_ H_76_ N_9_ O_7_ S	778.5595	(M+Na)^+^	801.5482	801.5482	−0.14	519.2919, 121.1020	MB
140	27.173	Unidentified	C_37_ H_67_ N_13_ O_3_	741.5491	(M+Na)^+^	764.5381	764.5382	0.15	102.0913	MB, MT
141	29.246	Unidentified	C_22_ H_48_ Cl_2_ N_5_ O_2_ S	516.2900	(M+H)^+^	517.2957	517.2979	4.07	312.0957, 244.0374, 175.9745	MB, MT
142	29.348	Unidentified	C_34_ H_68_	476.5322	(M+NH_4_)^+^	494.5662	494.5659	−0.53	453.3644, 271.3170, 151.1298	MB, MT
143	30.655	Lansiol [M, H]	C_33_ H_56_ O	468.4326	(M+CH_3_COO)^-^	527.4465	527.4470	0.91	478.6391, 447.7013, 413.8984, 365.2430, 305.1114, 258.1590, 192.0016	MB
144	32.152	Unidentified	C_6_ H_12_ N_6_ O_3_	216.0977	(M+H)^+^	217.1049	217.1044	−2.52	204.0959, 161.0979, 134.0842, 107.0513	MB

^a^ MB: MeOH crude extract of bark; MT: MeOH crude extract of trunk; DB: CH_2_Cl_2_ crude extract of bark; DT: CH_2_Cl_2_ crude extract of trunk.

**Table 2:** ^1^H (400 MHz) and ^13^C (100 MHz) NMR spectroscopic data for ventilatone C (**16**).

Position	Ventilatone C (16)			
	NMR Data in CDCl_3_		NMR Data in Acetone-*d_6_*	
	*δ*_H_, Multiplicity (*J* in Hz)	*δ*_C_, Type	*δ*_H_, Multiplicity (*J* in Hz)	*δ*_C_, Type
1	-	166.18, C	-	167.05, C
3	4.53, ddq (10.5, 6.3, 3.4)	74.97, CH	4.65, ddq (10.6, 6.3, 3.4)	76.06, CH
4	3.00, ddd (16.3, 10.6, 1.4) 3.15, ddd (16.4, 3.3, 0.8)	34.23, CH_2_	3.03, ddd (16.5, 10.7, 1.6) 3.25, ddd (16.5, 3.1, 0.8)	34.50, CH_2_
4a	-	127.70, C	-	129.66, C
5	7.24, s	120.60, CH	7.43, s	121.32, CH
5a	-	138.35, C	-	139.79, C
6	6.72, s	99.96, CH	6.94, d (2.3)	100.52, CH
7	-	161.13, C,	-	161.07, C
8	6.72, s	103.11, CH	6.64, d (2.3)	103.44, CH
9	-	156.05, C	-	156.74, C
9a	-	107.00, C	-	107.90, C
10	-	152.72, C	-	153.48, C
10a	-	104.37, C	-	105.33, C
12	-	161.72, C	-	162.44, C
13	5.74, s	90.81, CH	5.63, s	90.82, CH
3-Me	1.56, d (6.4)	20.79, CH_3_	1.54, d (6.3)	20.83, CH_3_
7-OMe	3.91, s	55.50, CH_3_	3.92, s	55.88, CH_3_
9-OH	8.81, br s	-	8.94, s	-

**Table 3:** Antibacterial and antifungal activities of crude extracts, fractions and isolated compounds.

Crude Extracts/ Fractions/ Compounds	Zone of Inhibition (mm)					
	Bacteria/Fungus					
	*B. cereus*	*S. aureus*	*E. coli*	*S. enterica*	*P. aeruginosa*	*C. albicans*
MB ^a^	13	15	8	14	10	13
DB ^a^	21	18	9	19	8	16
MT ^a^	7	14	8	7	7	8
DT ^a^	0	13	0	0	0	0
FM1 ^b^	11	14	9	0	10	0
FM2 ^b^	11	12	9	8	8	10
FM3 ^b^	9	7	12	10	8	0
FM4 ^b^	0	0	0	14	0	0
FM5 ^b^	0	0	0	0	0	0
FM6 ^b^	0	0	9	9	0	0
FD1 ^c^	18	15	14	18	11	17
FD2 ^c^	19	22	17	23	9	0
FD3 ^c^	26	25	25	30	12	0
FD4 ^c^	17	12	13	24	12	0
FD5 ^c^	14	15	16	14	9	0
FD6 ^c^	11	17	11	16	10	9
**7**	0	11	0	0	0	8
**8**	10	0	11	0	0	12
**9**	9	9	0	0	13	6
**10**	9	11	12	0	0	0
**11**	9	14	10	0	0	0
**12**	11	11	0	18	0	12
**13**	ND	7	ND	ND	0	0
**15**	13	17	ND	18	0	0
**16**	13	13	ND	14	0	0
Chloramphenicol **^d^**	44	37	50	50	28	ND
Tetracycline **^d^**	40	39	40	44	29	ND
Amphotericin B **^e^**	ND	ND	ND	ND	ND	23

^a^ MB: MeOH crude extract of bark; DB: CH_2_Cl_2_ crude extract of bark; MT: MeOH crude extract of trunk; DT: CH_2_Cl_2_ crude extract of trunk. ^b^ FM1-FM6: Fractions obtained from HPLC isolation of MeOH crude extract of bark eluted at retention times (t_R_) of 1.0–6.0 min (FM1), 6.0–8.5 min (FM2), 8.5–12.0 min (FM3), 12.0–20.0 min (FM4), 20.0–28.0 min (FM5) and 28.0–34.0 min (FM6), respectively. HPLC conditions are in the Section 3.9. ^c^ FD1-FD6: HPLC fractions from CH_2_Cl_2_ crude extract of bark eluted at retention times (t_R_) of 1.0–5.5 min (FD1), 5.5–6.6 min (FD2), 6.6–7.1 min (FD3), 7.1–8.3 min (FD4), 8.3–9.5 min (FD5) and 9.5–13.0 min (FD6), respectively. HPLC conditions are in the Section 3.9. ^d^ Chloramphenicol and tetracycline are standard drugs for antibacterial activity. ^e^ Amphotericin B is a standard drug for antifungal activity. ND = Not determined.

**Table 4:** Antibacterial and antifungal agents from the HPLC fractions of *V. denticulata*, tentatively identified by ESI-HRMS analysis based on the putative compounds listed in Table 1.

Fraction	Compounds in Fractions
FM1 ^a^	Kaempferol (285.0391 [M-H]^−^), chrysoeriol (299.0589 [M-H]^−^), unidentified C_13_H_20_N_3_O_8_S (377.0851 [M-H]^−^), kaempferol 3-rhamninoside (739.2091 [M-H]^−^), isopimpinellin (305.0657 [M+CH_3_COO]^−^), 3-hydroxyphloretin (335.0760 [M+HCOO]^−^), rhamnocitrin 3-rhamninoside (377.0851 [M-H]^−^), unidentified C_37_H_32_N_3_O_15_ (757.1768 [M-H]^−^), rhamnetin 3-rhamninoside (769.2162 [M-H]^−^), rhamnazin 3-rhamninoside (783.2310 [M-H]^−^)
FM2 ^a^	Rhamnetin (315.0475 [M-H]^−^), luteolin (285.0391 [M-H]^−^), 3,5,7-trihydroxy-4′,6-dimethoxyflavanone (377.0846 [M+CH_3_COO]^−^)
FM3 ^a^	Emodin (269.0445 [M-H]^−^), rhamnocitrin (299.0563 [M-H]^−^), palmidin A (509.1218 [M-H]^−^), unidentified (523.1351 [M-H]^−^)
FD1 ^b^	Eriodyctiol (287.0554 [M-H]^−^), cartorimine (289.0705 [M-H]^−^), chrysoeriol (299.0558 [M-H]^−^), rhamnetin (315.0507 [M-H]^−^), 3-hydroxyphloretin (335.0771 [M+HCOO]^−^), xanthotoxol glucoside (363.0709 [M-H]^−^), furocoumarinic acid glucoside (365.0875 [M-H]^−^)
FD2 ^b^	Ventilagodenin A (275.0846 [M-H]^−^), physcion (283.0650 [M-H]^−^), rhamnocitrin (299.0613 [M-H]^−^), ventilatone A (311.0602 [M-H]^−^), 3′,7-dihydroxy-4′,8-dimethoxyisoflavone (313.0761 [M-H]^−^), rhamnazin (329.0726 [M-H]^−^), 3,5,7-trihydroxy-4′,6-dimethoxyflavanone (331.0803 [M-H]^−^), ventilatone B (327.0556 [M-H]^−^), unidentified C_17_H_18_O_8_ (349.0965 [M-H]^−^)
FD3 ^b^	Afzelechin (273.0727 [M-H]^−^), (+)-(*R*)-ventilagolin (331.0827 [M-H]^−^), mukurozidiol (333.0968 [M-H]^−^)
FD4 ^b^	Emodin (269.0450 [M-H]^−^), 6α-hydroxymaackiain (299.0550 [M-H]^−^), 2′,3,5-trihydroxy-5′,7-dimethoxyflavanone (331.0816 [M-H]^−^), palmidin A (509.1261 [M-H]^−^), unidentified C_15_H_11_O_4_ (254.0592 [M-H]^−^), unidentified C_29_H_22_N_3_O_7_ (523.1416 [M-H]^−^),

^a^ FM1-FM3 = Fractions obtained from HPLC isolation of MeOH crude extract of bark eluted at retention times (t_R_) of 1.0–6.0 min (FM1), 6.0–8.5 min (FM2) and 8.5–12.0 min (FM3), respectively. ^b^ FD1-FD4 = Fractions from HPLC isolation of CH_2_Cl_2_ crude extract of bark eluted at retention times (t_R_) of 1.0–5.5 min (FD1), 5.5–6.6 min (FD2), 6.6–7.1 min (FD3) and 7.1–8.3 min (FD4), respectively. HPLC conditions are in the Section 3.9.

## Supplementary Materials

The following supporting materials are available online at https://www.mdpi.com/2079-6382/9/9/606/s1 (Figures S1–S46); Overlay of TIC chromatograms of MeOH and CH_2_Cl_2_ crude extracts (Figure S1); molecular networking of crude extracts in a negative ionization mode (Figure S2); MS/MS spectra of compounds **1** and **2**–**8** (Figures S3–S9); ^1^H, ^13^C NMR and MS spectra of compounds **7**–**11** (Figures S10–S24); MS/MS spectra of ventilatone B (**12**) and ventilatone A (**15**) (Figures S25 and S26); ^1^H, ^13^C NMR and MS spectra of compounds **12**, **13** and **15** (Figures S27–S34); and 1D and 2D NMR spectra, MS and UV spectra of ventilatone C (**16**) (Figures S35–S46).
