# Supplementary material for: UHPLC-ESI-QTOF-MS/MS-Based Molecular Networking Guided Isolation and Dereplication of Antibacterial and Antifungal Constituents of Ventilago denticulata
Source: Antibiotics (Basel). 2020 Sep 15;9(9):606. doi: 10.3390/antibiotics9090606 (PMC7558283; doi:10.3390/antibiotics9090606)

antibiotics

Supplementary data

UHPLC-ESI-QTOF-MS/MS-Based Molecular Networking Guided Isolation and Dereplication of Antibacterial and Antifungal Constituents of *Ventilago denticulata*

Muhaiminatul Azizah [1], Patcharee Pripdeevech [2,3], Tawatchai Thongkongkaew [1], Chulabhorn Mahidol [1,4], Somsak Ruchirawat [1,4,5] and Prasat Kittakoop [1,4,5]*

1 Chulabhorn Graduate Institute, Chemical Biology Program, Chulabhorn Royal Academy, Laksi, Bangkok 10210, Thailand; prasat@cri.or.th (P.K.); mimi.hufflepuf@gmail.com (M.A.); tawatchait@cgi.ac.th (T.T.)
2 School of Science, Mae Fah Luang University, Muang, Chiang Rai 57100, Thailand; patcharee.pri@mfu.ac.th (P.P.)
3 Center of Chemical Innovation for Sustainability (CIS), Mae Fah Luang University, Muang, Chiang Rai 57100, Thailand
4 Chulabhorn Research Institute, Kamphaeng Phet 6 Road, Laksi, Bangkok 10210, Thailand; mahidol_natlab@cri.or.th (C.M.); somsak@cri.or.th (S.R.)
5 Center of Excellence on Environmental Health and Toxicology (EHT), CHE, Ministry of Education, Bangkok, Thailand
* Correspondence: prasat@cri.or.th; Tel.: +66869755777 (P.K.)

Figure S1. Overlay of TIC chromatograms of MeOH crude extract of bark, MeOH crude extract of trunk, CH_2Cl_2 crude extract of bark, and CH_2Cl_2 crude extract of trunk.
Figure S2. Molecular networking of crude extracts from *V. denticulata* in a negative ionization mode
Figure S3. MS/MS spectra of (+)-(*R*)-ventilagolin (**1**) in a positive ionization mode
Figure S4. MS/MS spectra of a putative new compound of (+)-(*R*)-ventilagolin derivative (**3**) or (**4**) in a positive ionization mode
Figure S5. MS/MS spectrum of rutin (**2**) parent ion at m/z 633.1422 $[M+Na]^+$
Figure S6. MS/MS spectrum of rhamnazin 3-rhamninoside (**7**) parent ion at m/z 785.2430 $[M+H]^+$
Figure S7. MS/MS spectrum of rhamnocitrin 3-rhamninoside (**8**) parent ion at m/z 755.2394 $[M+H]^+$
Figure S8. MS/MS spectrum of rhamnetin 3-rhamninoside (**9**) parent ion at m/z 771.2343 $[M+H]^+$
Figure S9. MS/MS spectrum of kaempferol 3-rhamninoside (**10**) parent ion at m/z 741.2233 $[M+H]^+$
Figure S10. ^{1}H NMR (400 MHz) spectrum of rhamnazin 3-rhamninoside (**7**) in methanol-d_4
Figure S11. ^{13}C NMR (100 MHz) spectrum of rhamnazin 3-rhamninoside (**7**) in methanol-d_4
Figure S12. ESI-HRMS spectrum of rhamnazin 3-rhamninoside (**7**) in a negative ionization mode
Figure S13. ^{1}H NMR (400 MHz) spectrum of rhamnocitrin 3-rhamninoside (**8**) in methanol-d_4
Figure S14. ^{13}C NMR (100 MHz) spectrum of rhamnocitrin 3-rhamninoside (**8**) in methanol-d_4
Figure S15. ESI-HRMS spectrum of rhamnocitrin 3-rhamninoside (**8**) in a negative ionization mode
Figure S16. ^{1}H NMR (400 MHz) spectrum of rhamnetin 3-rhamninoside (**9**) in methanol-d_4
Figure S17. ^{13}C NMR (100 MHz) spectrum of rhamnetin 3-rhamninoside (**9**) in methanol-d_4
Figure S18. ESI-HRMS spectrum of rhamnetin 3-rhamninoside (**9**) in a negative ionization mode
Figure S19. ^{1}H NMR (400 MHz) spectrum of kaempferol 3-rhamninoside (**10**) in methanol-d_4
Figure S20. ^{13}C NMR (100 MHz) spectrum of kaempferol 3-rhamninoside (**10**) in methanol-d_4
Figure S21. ESI-HRMS spectrum of kaempferol 3-rhamninoside (**10**) in a negative ionization mode
Figure S22. ^{1}H NMR (400 MHz) spectrum of quercetin 3-rhamninoside (**11**) in methanol-d_4

Figure S23. ^{13}C NMR (100 MHz) spectrum of quercetin 3-rhamninoside (**11**) in methanol-d_4

Figure S24. ESI-HRMS spectrum of quercetin 3-rhamninoside (**11**) in a negative ionization mode

Figure S25. MS/MS spectrum of ventilatone B (**12**) parent ion at *m/z* 329.0659 $[M+H]^+$

Figure S26. MS/MS spectrum of ventilatone A (**15**) parent ion at *m/z* 313.0706 $[M+H]^+$

Figure S27. ^1H NMR (400 MHz) spectrum of ventilatone B (**12**) in $CDCl_3$

Figure S28. ^{13}C NMR (100 MHz) spectrum of ventilatone B (**12**) in $CDCl_3$

Figure S29. ESI-HRMS spectrum of ventilatone B (**12**) in a negative ionization mode

Figure S30. ^1H NMR (400 MHz) spectrum of lupeol (**13**) in $CDCl_3$

Figure **S31.** ^{13}C NMR (100 MHz) spectrum of lupeol (**13**) in $CDCl_3$

Figure S32. ^1H NMR (400 MHz) spectrum of ventilatone A (**15**) in $CDCl_3$

Figure S33. ^{13}C NMR (100 MHz) spectrum of ventilatone A (**15**) in $CDCl_3$

Figure S34. ESI-HRMS spectrum of ventilatone A (**15**) in a negative ionization mode

Figure S35. ^1H NMR (400 MHz) spectrum of ventilatone C (**16**) in $CDCl_3$

Figure S36. ^{13}CNMR (100 MHz) spectrum of ventilatone C (**16**) in $CDCl_3$

Figure S37. ^1H-^1H COSY spectrum of ventilatone C (**16**) in $CDCl_3$

Figure S38. HSQC spectrum of ventilatone C (**16**) in $CDCl_3$

Figure S39. HMBC spectrum of ventilatone C (**16**) in $CDCl_3$

Figure S40. ^1H NMR (400 MHz) spectrum of ventilatone C (**16**) in acetone-d_6

Figure S41. ^{13}C NMR (100 MHz) spectrum of ventilatone C (**16**) in acetone-d_6

Figure S42. ^1H-^1H COSY spectrum of ventilatone C (**16**) in acetone-d_6

Figure S43. HSQC spectrum of ventilatone C (**16**) in acetone-d_6

Figure S44. HMBC spectrum of ventilatone C (**16**) in acetone-d_6

Figure S45. ESI-HRMS spectrum of ventilatone C (**16**) in a positive ionization mode

Figure S46. UV spectrum of ventilatone C (**16**) in CH_3CN

Figure S1. Overlay of TIC chromatograms of MeOH crude extract of bark, MeOH crude extract of trunk, CH_2Cl_2 crude extract of bark, and CH_2Cl_2 crude extract of trunk.

Crude extracts were analyzed by UHPLC connected to Q-TOF MS. UHPLC column was ACE Excel C_{18} AR (100 x 2.1 mm,1.7 µm), and a flow rate was 0.2 mL/min with an injection volume of 0.5 µL. The gradient elution was performed using the following conditions: (i) linear gradient from 40% CH_3CN (0.1% formic acid) in H_2O (0.1% formic acid) to 100% CH_3CN (0.1% formic acid) for 0-25 min, (ii) isocratic elution of 100% CH_3CN (0.1% formic acid) for 5 min (at time of 25-30 min), (iii) a linear gradient from 100% CH_3CN (0.1% formic acid) to 40% CH_3CN (0.1% formic acid) in H_2O (0.1% formic acid) for 4 min (at time of 30-34 min, and (iv) equilibrium time by isocratic elution with 40% CH_3CN (0.1% formic acid) in H_2O (0.1% formic acid) for 6 min (at time of 34-40 min). The total run time was 40 min.

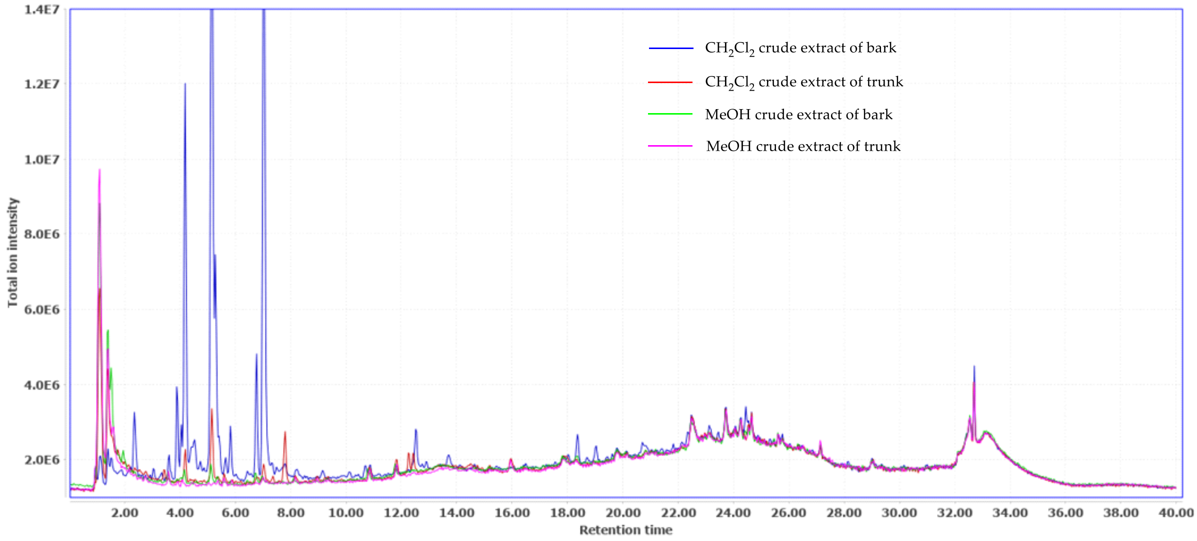

Figure S2. Molecular networking of crude extracts from *V. denticulata* in a negative ionization mode

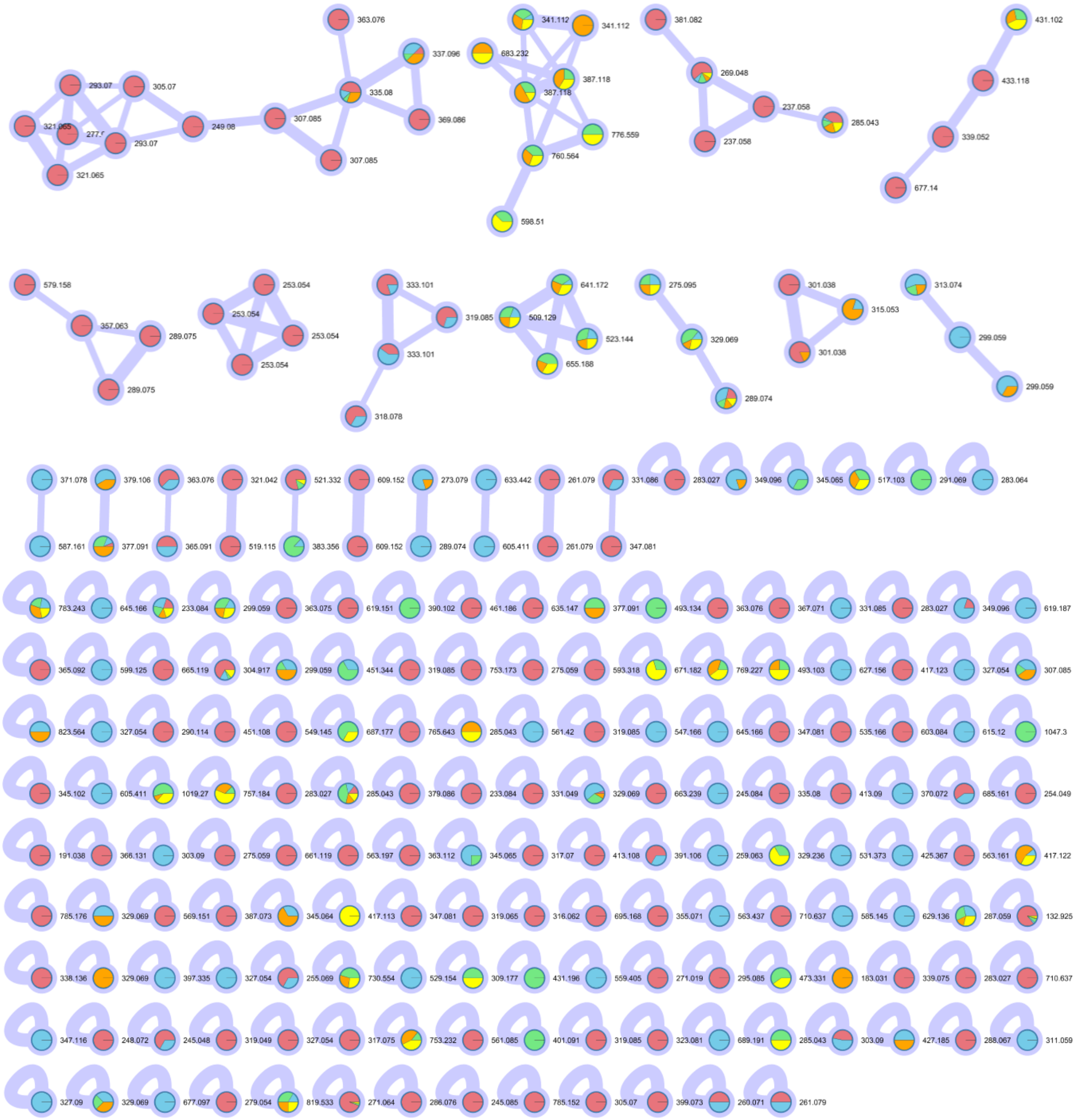

Figure S3. MS/MS spectra of (+)-(*R*)-ventilagolin (**1**) in a positive ionization mode

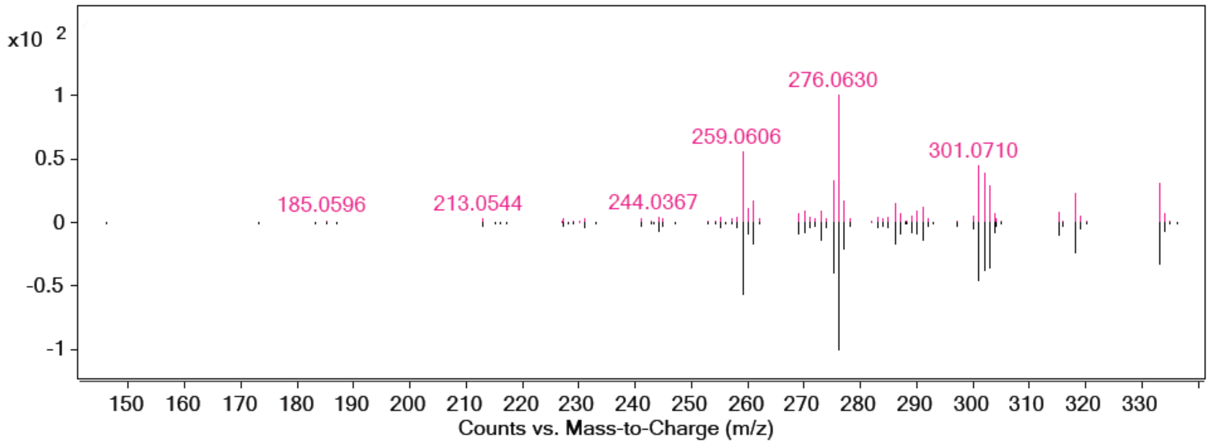

Figure S4. MS/MS spectra of a putative analog compound of (+)-(*R*)-ventilagolin (**3**) or (**4**) in a positive ionization mode

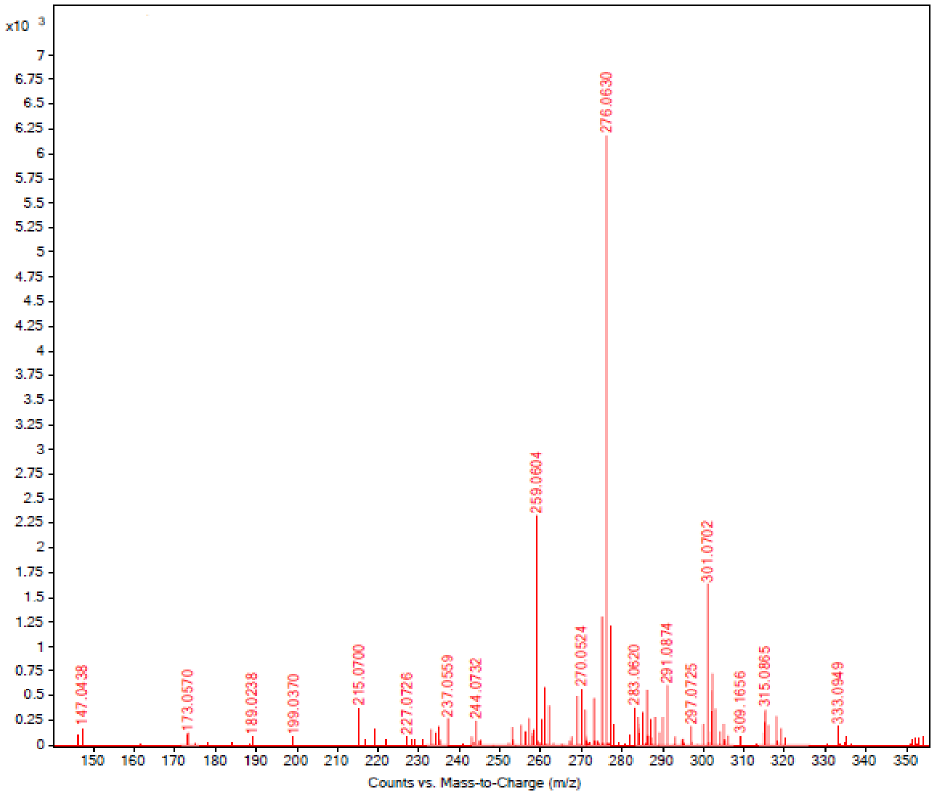

Figure S5. MS/MS spectrum of rutin (**2**) parent ion at *m/z* 633.1422 [M+Na]⁺

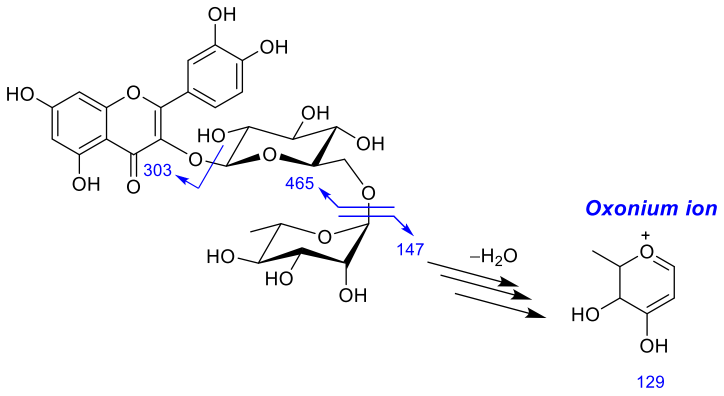

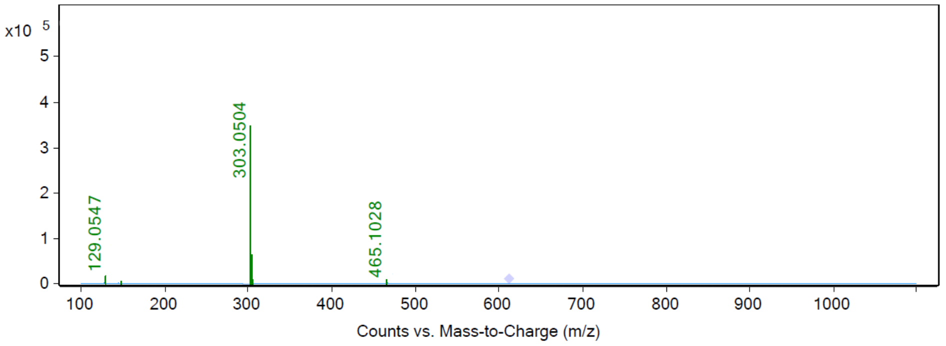

m/z	Calc *m/z*	Diff(ppm)	z	Abund	Formula	Ion
→ 129.0547	129.0546	-0.26	1	19122.84	C6 H9 O3	M+
→ 147.0653	147.0652	-0.46	1	4683.12	C6 H11 O4	M+
→ 303.0504	303.0499	-1.56	1	354485.83	C15 H11 O7	M+
304.0537	304.0533	-1.22	1	65541.49	C15 H11 O7	M+
305.0554	305.0554	-0.02	1	9412.25	C15 H11 O7	M+
→ 465.1028	465.1028	-0.02	1	7947.04	C21 H21 O12	M+

Figure S6. MS/MS spectrum of rhamnazin 3-rhamninoside (**7**) parent ion at *m/z* 785.2430 [M+H]+

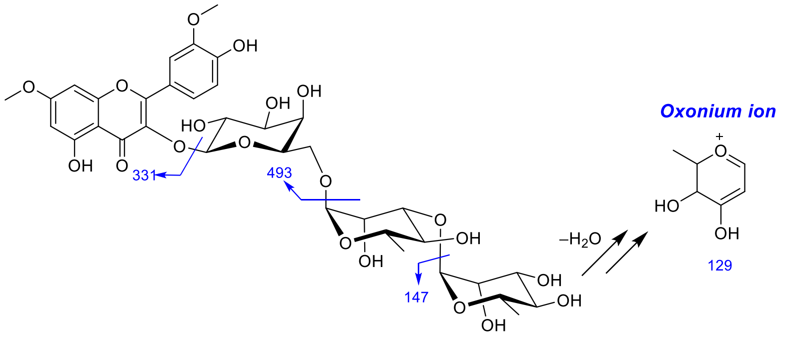

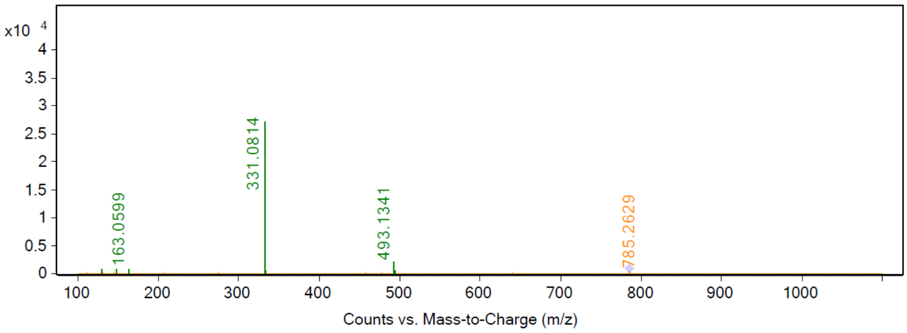

m/z	Calc *m/z*	Diff(ppm)	z	Abund
→ 129.054	129.0546	4.75	1	734.81
→ 147.0653	147.0652	-0.82	1	588.41
→ 163.0599	163.0588	-6.97	1	744.76
→ 331.0814	331.0812	-0.59	1	27225.84
332.0841	332.0838	-1	1	5593.48
333.0868	333.0862	-1.79	1	389.38
→ 493.1341	493.1367	5.28	1	2089.53
494.1361	494.1395	6.96	1	410.03
785.2629				447.26

Figure S7. MS/MS spectrum of rhamnocitrin 3-rhamninoside (**8**) parent ion at *m/z* 755.2394 [M+H]⁺

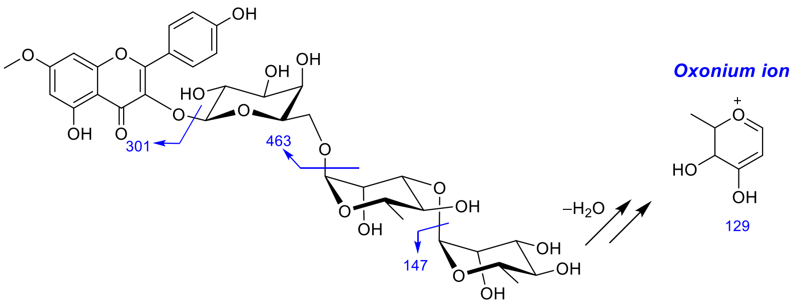

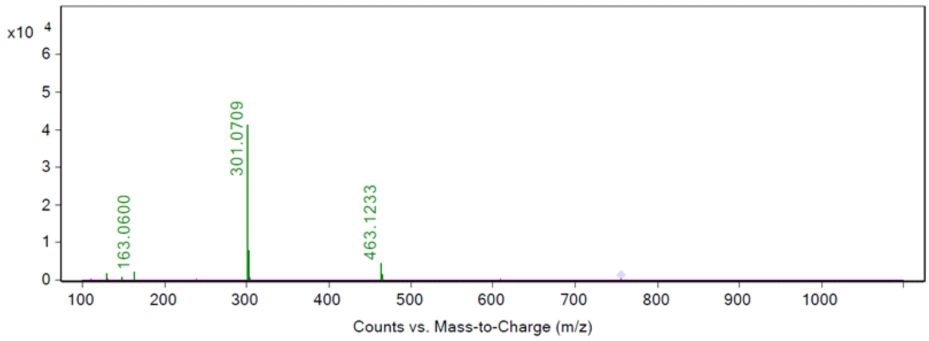

m/z	Calc *m/z*	Diff(ppm)	z	Abund	Formula	Ion
→ 129.0543	129.0546	2.48	1	1820.91	C6 H9 O3	M+
→ 147.0651	147.0652	0.39	1	633.51	C6 H11 O4	M+
→ 163.06	163.0601	0.46	1	1941.96	C6 H11 O5	M+
→ 301.0709	301.0707	-0.64	1	41217.6	C16 H13 O6	M+
302.074	302.0741	0.03	1	7913.13	C16 H13 O6	M+
303.0763	303.0763	0.07	1	1178.09	C16 H13 O6	M+
→ 463.1233	463.1235	0.41	1	4554.14	C22 H23 O11	M+
464.127	464.1269	-0.18	1	1349.28	C22 H23 O11	M+

Figure S8. MS/MS spectrum of rhamnetin 3-rhamninoside (**9**) parent ion at *m/z* 771.2343 [M+H]+

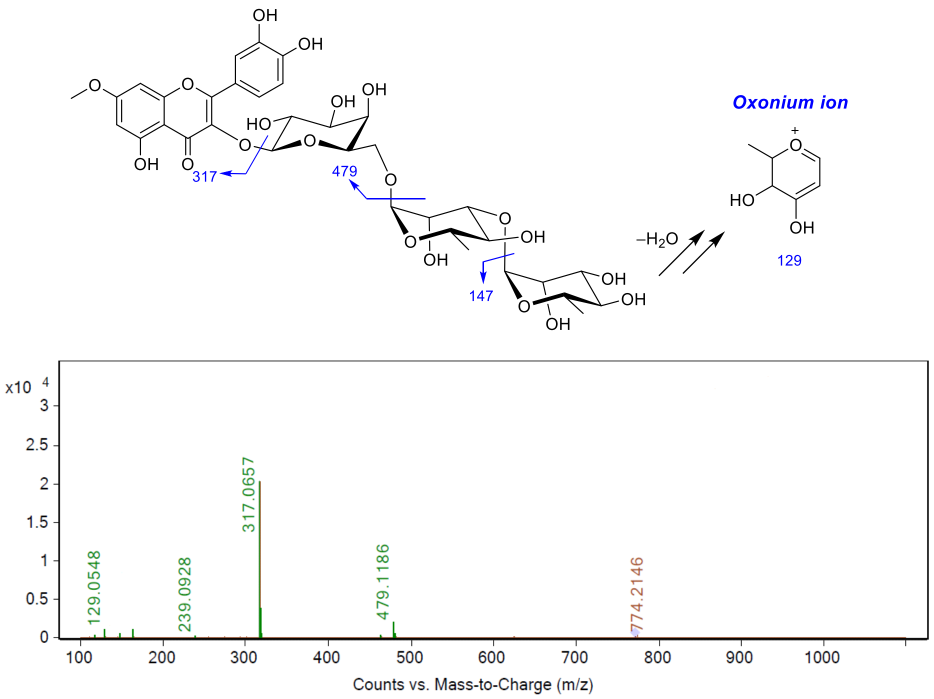

m/z	Calc *m/z*	Diff(ppm)	z	Abund	Formula	Ion
117.0541	117.0546	4.59	1	366.75	C5 H9 O3	M+
→ 129.0548	129.0546	-1.49	1	1200.41	C6 H9 O3	M+
→ 147.0653	147.0652	-0.51	1	581.61	C6 H11 O4	M+
→ 163.0602	163.0601	-0.57	1	1033.11	C6 H11 O5	M+
→ 317.0657	317.0656	-0.47	1	21235	C16 H13 O7	M+
318.0692	318.069	-0.84	1	3967.16	C16 H13 O7	M+
319.0717	319.0711	-1.74	1	543.41	C16 H13 O7	M+
→ 479.1186	479.1184	-0.44	1	1983.18	C22 H23 O12	M+
480.1232	480.1218	-2.81	1	527.01	C22 H23 O12	M+
774.2146			2	332.07		

Figure S9. MS/MS spectrum of kaempferol 3-rhamninoside (**10**) parent ion at *m/z* 741.2233 [M+H]+

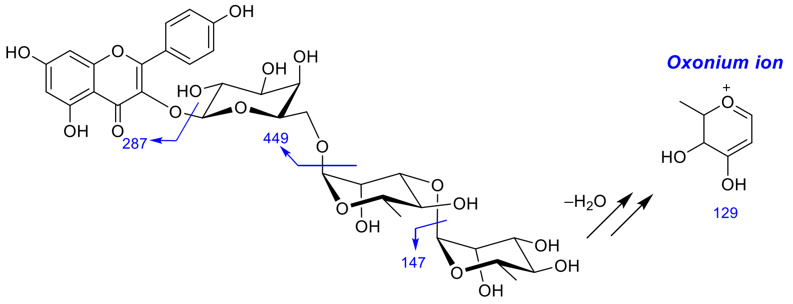

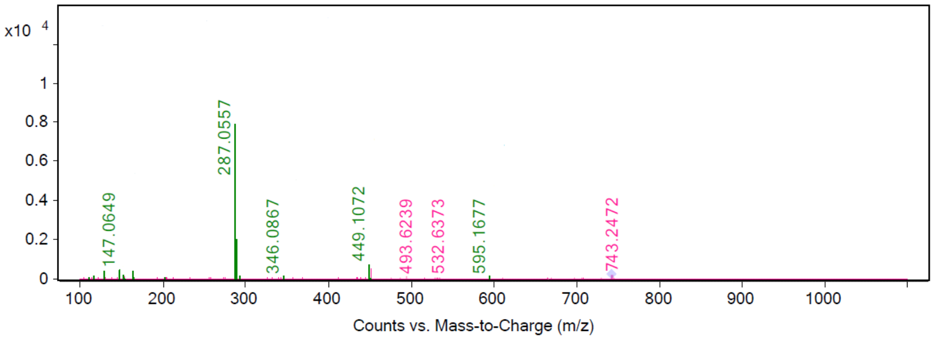

m/z	Calc *m/z*	Diff(ppm)	z	Abund	Formula	Ion
→ 129.0535	129.0546	8.6	1	415.27	C6 H9 O3	M+
→ 147.0649	147.0652	1.87	1	421.79	C6 H11 O4	M+
→ 163.0597	163.0601	2.62	1	374.84	C6 H11 O5	M+
→ 287.0557	287.055	-2.31	1	7893.47	C15 H11 O6	M+
288.0582	288.0584	0.71	1	1992.06	C15 H11 O6	M+
289.0614	289.0606	-2.98	1	305.41	C15 H11 O6	M+
→ 449.1072	449.1078	1.37	1	749.58	C21 H21 O11	M+
450.1112	450.1098	-3.11	1	567.32	C28 H18 O6	M+
741.2358				196.44		
743.2472				210.43		

Figure S10. ^{1}H NMR (400 MHz) spectrum of rhamnazin 3-rhamninoside (**7**) in methanol-d_4

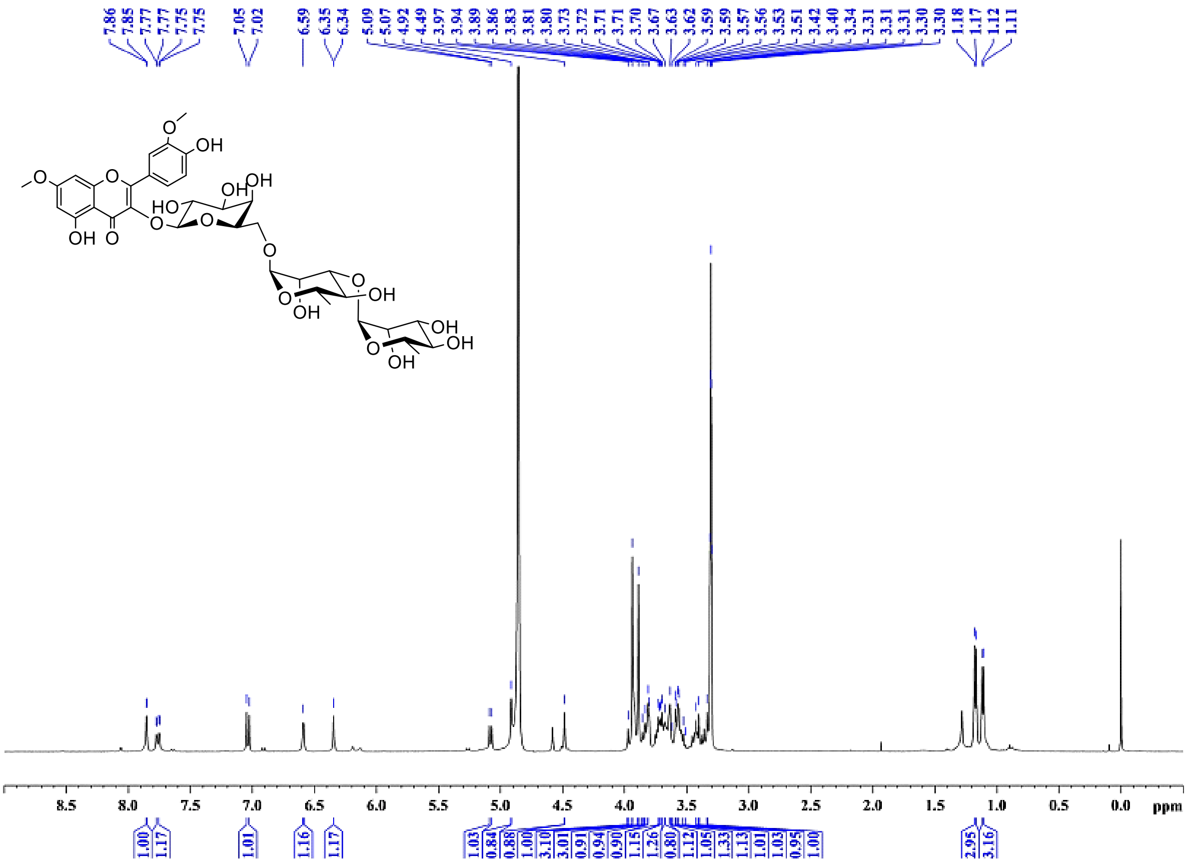

Figure S11. ^{13}C NMR (100 MHz) spectrum of rhamnazin 3-rhamninoside (**7**) in methanol-d_4

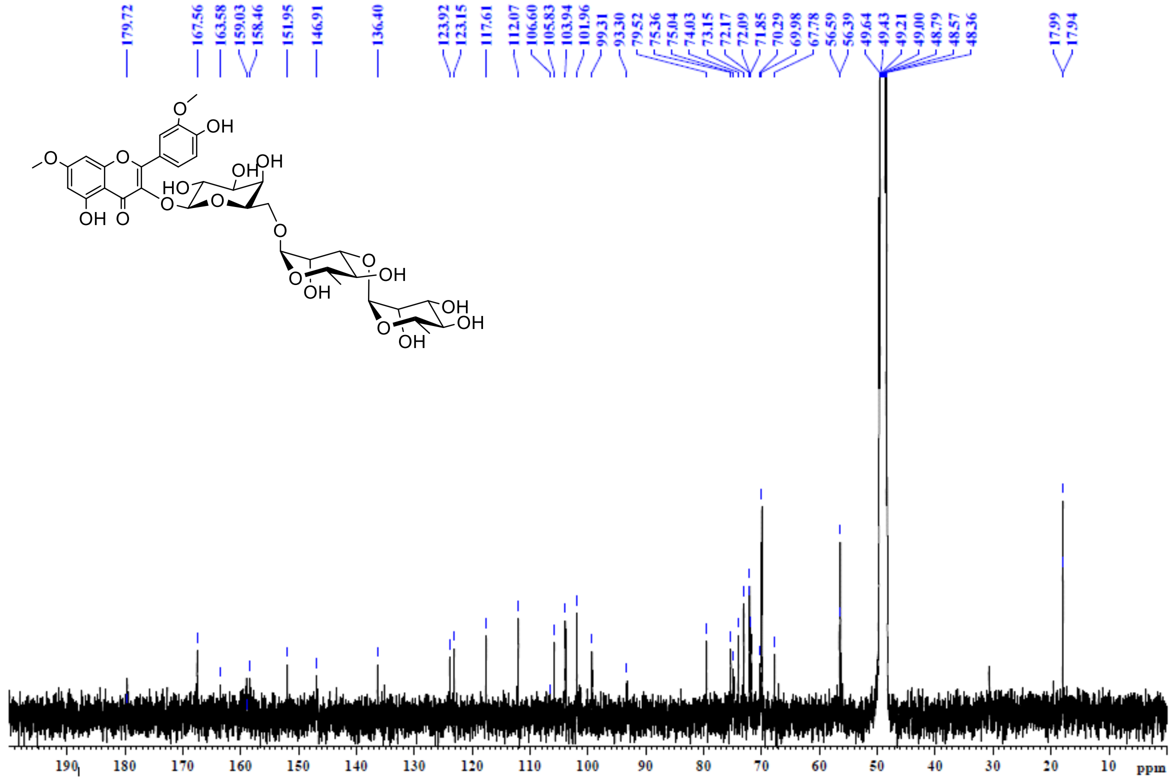

Figure S12. ESI-HRMS spectrum of rhamnazin 3-rhamninoside (**7**) in a negative ionization mode

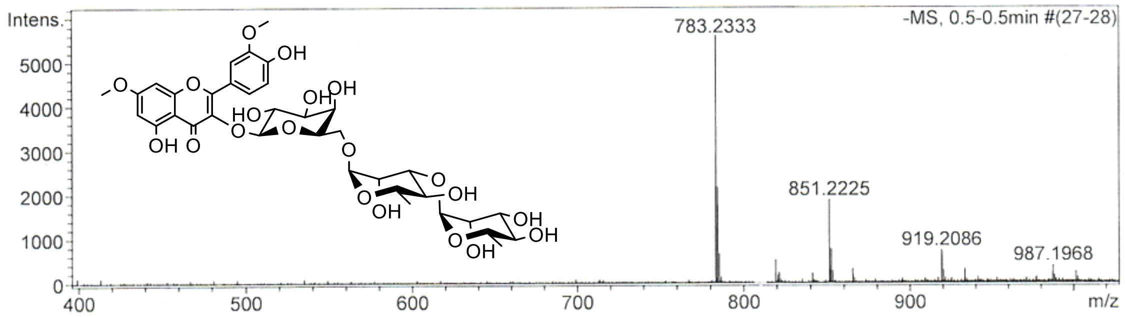

Rhamnazin 3-rhamninoside (**7**) had the observed precursor ion at *m/z* 783.2333 [M-H]$^-$, calcd for [$C_{35}H_{44}O_{20}$ - H]$^-$, 783.2348, $\Delta_{m/z}$ = 1.92 ppm, and thus having the molecular formula of $C_{35}H_{44}O_{20}$.

Figure S13. ^{1}H NMR (400 MHz) spectrum of rhamnocitrin 3-rhamninoside (**8**) in methanol-d_4

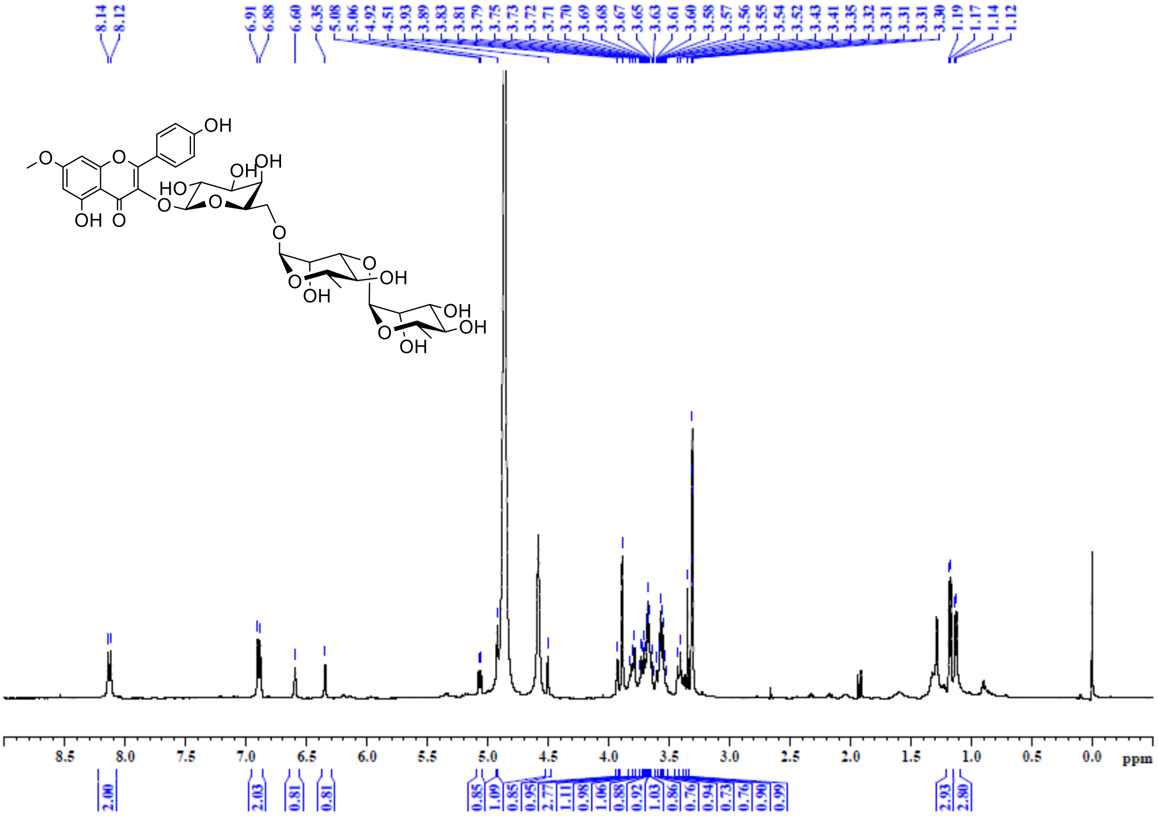

Figure S14. ^{13}C NMR (100 MHz) spectrum of rhamnocitrin 3-rhamninoside (**8**) in methanol-d_4

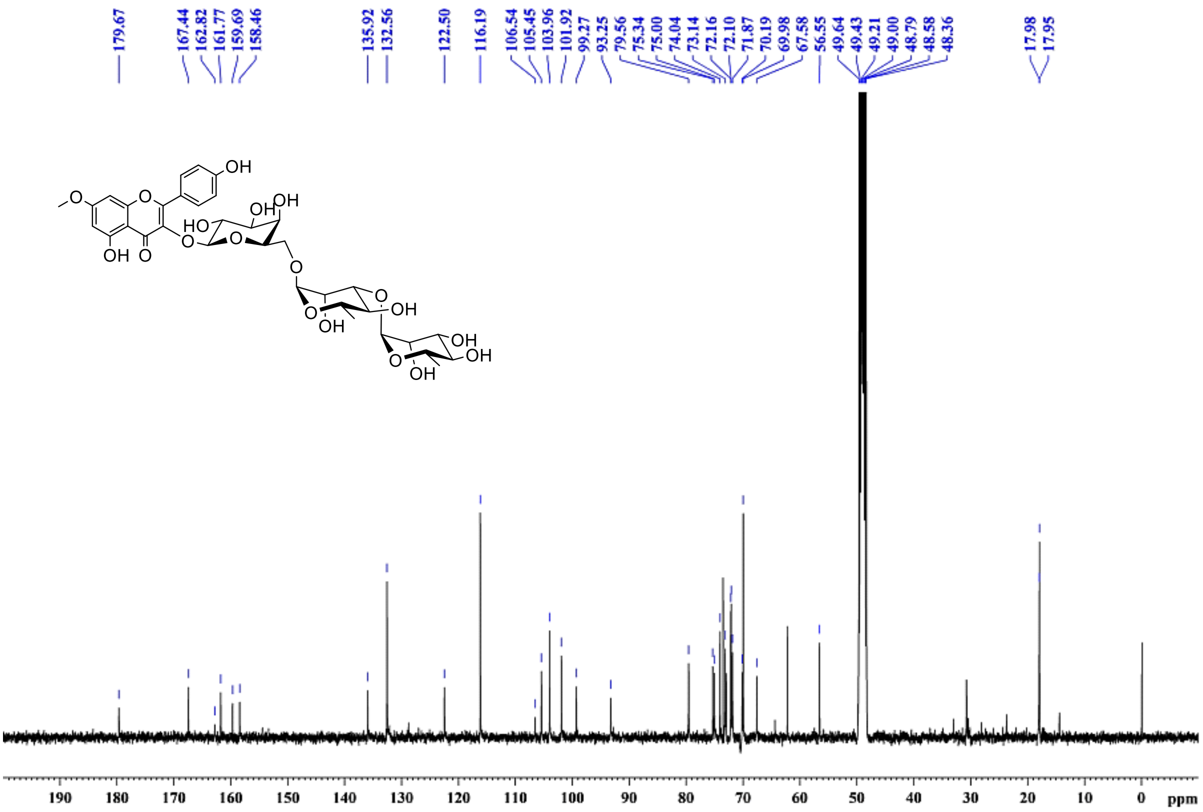

Figure S15. ESI-HRMS spectrum of rhamnocitrin 3-rhamninoside (**8**) in negative ionization mode

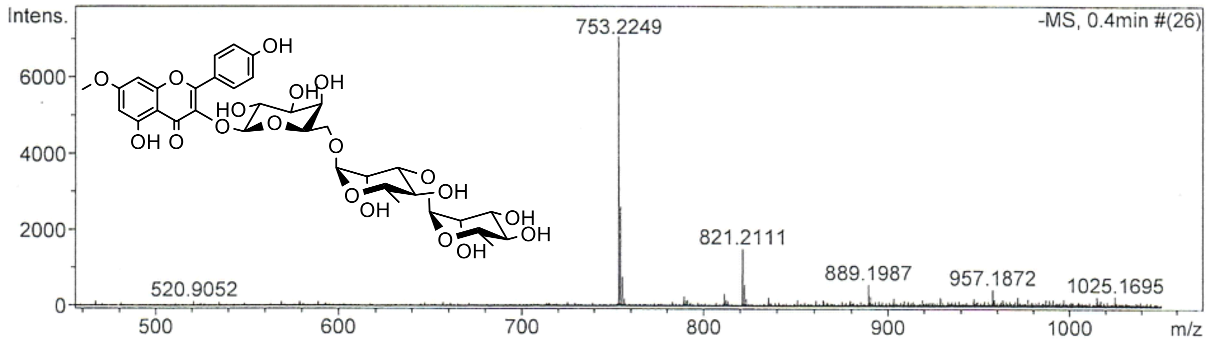

Rhamnocitrin 3-rhamninoside (8) had the observed precursor ion at *m/z* 753.2249 [M-H]$^-$, calcd for [$C_{34}H_{42}O_{19}$ - H]$^-$, 753.2242, $\Delta_{m/z}$ = 0.93 ppm, and thus having the molecular formula of $C_{34}H_{42}O_{19}$.

Figure S16. [13]HNMR (400 MHz) spectrum of rhamnetin 3-rhamninoside (**9**) in methanol-d_4

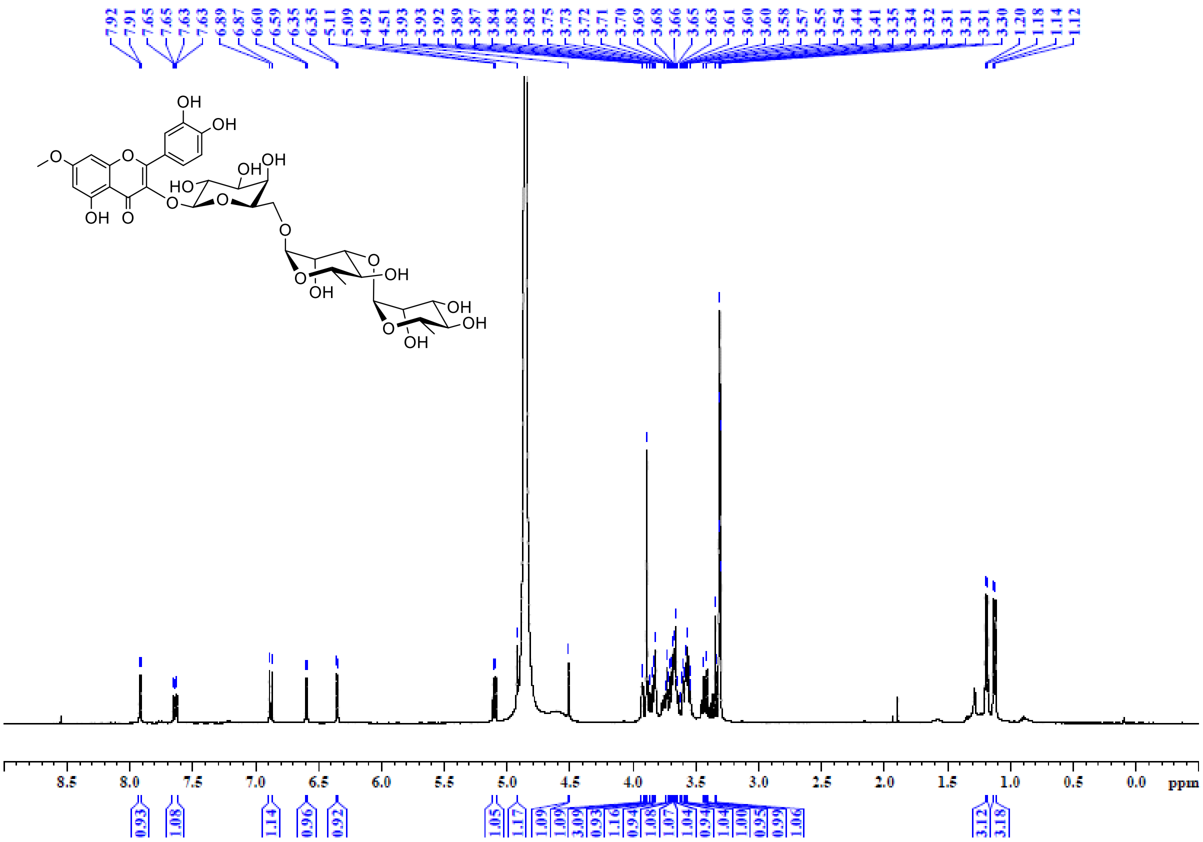

Figure S17. ^{13}CNMR (100 MHz) spectrum of rhamnetin 3-rhamninoside (**9**) in methanol-d_4

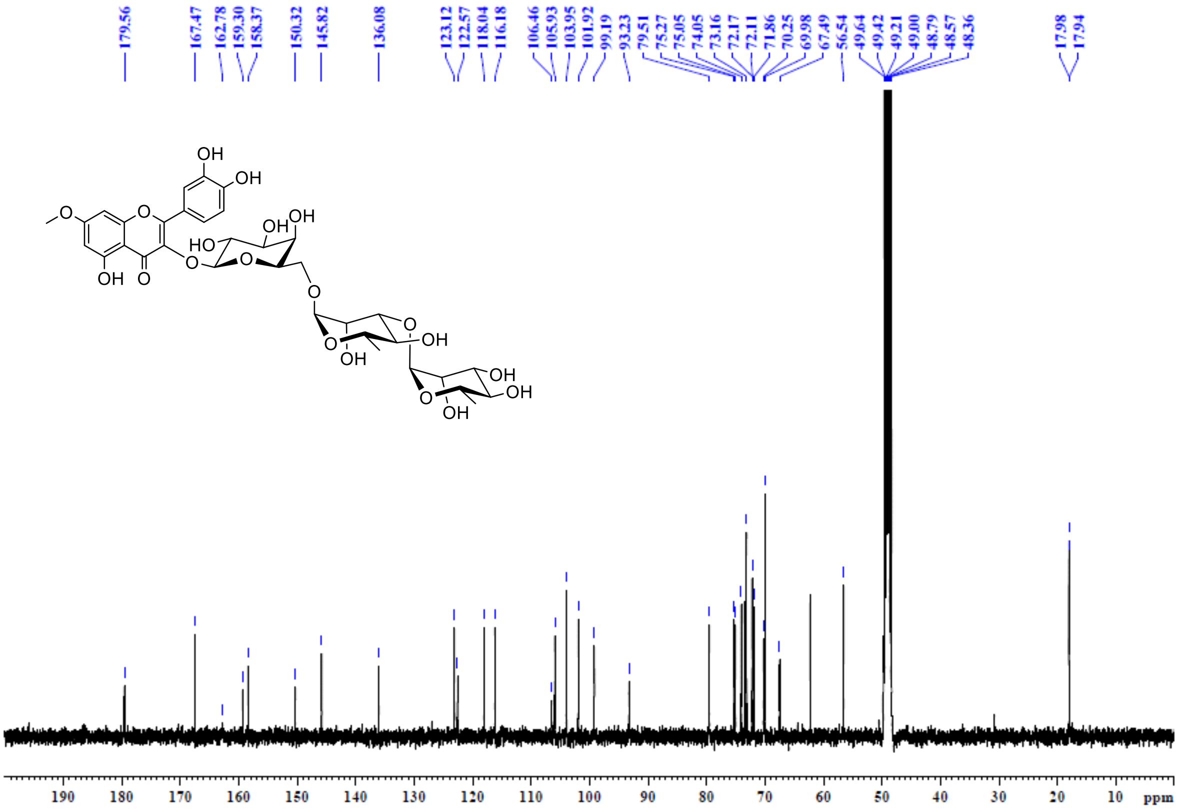

Figure S18. ESI-HRMS spectrum of rhamnetin 3-rhamninoside (**9**) in a negative ionization mode

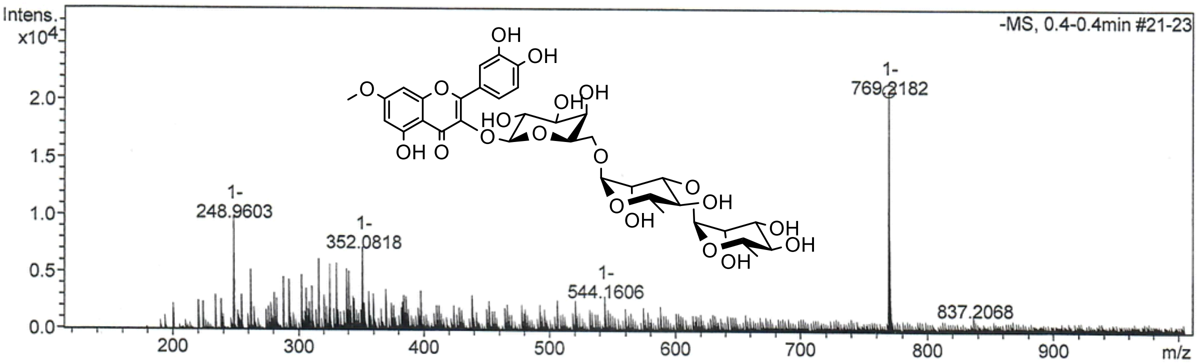

Rhamnetin 3-rhamninoside (**9**) had the observed precursor ion at *m/z* 769.2182 [M-H]⁻, calcd for [$C_{34}H_{42}O_{20}$ - H]⁻, 769.2191, $\Delta_{m/z}$ = 1.17 ppm, and thus having the molecular formula of $C_{34}H_{42}O_{20}$.

Figure S19. ^{1}HNMR (400 MHz) spectrum of kaempferol 3-rhamninoside (**10**) in methanol-*d₄*

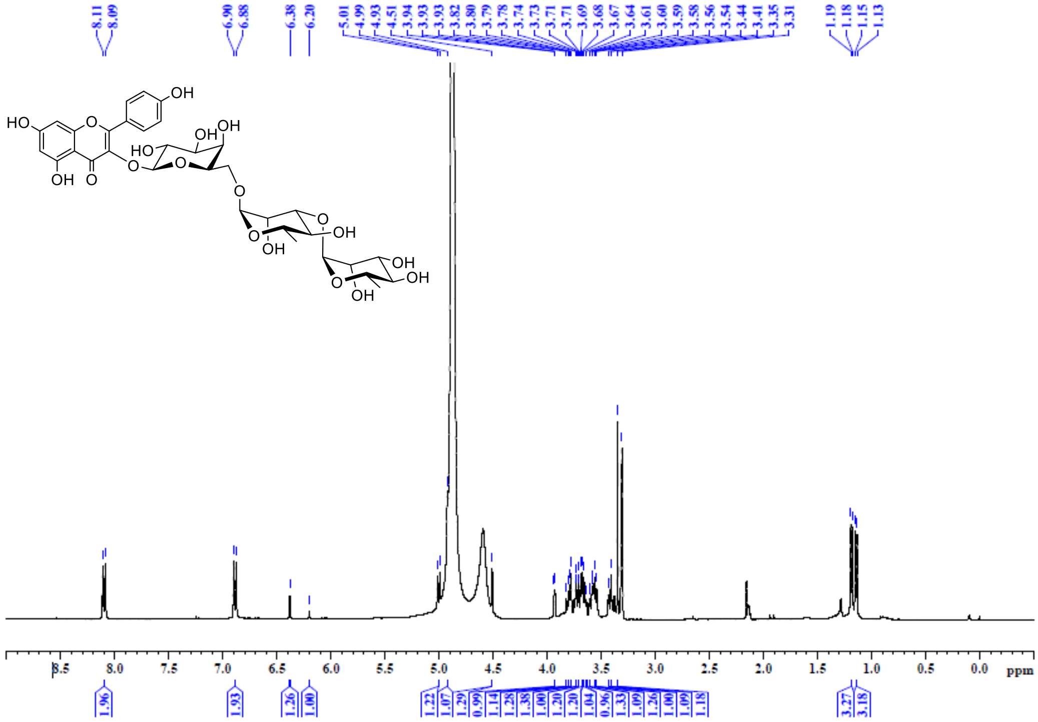

Figure S20. ^{13}CNMR (100 MHz) spectrum of kaempferol 3-rhamninoside (**10**) in methanol-d_4

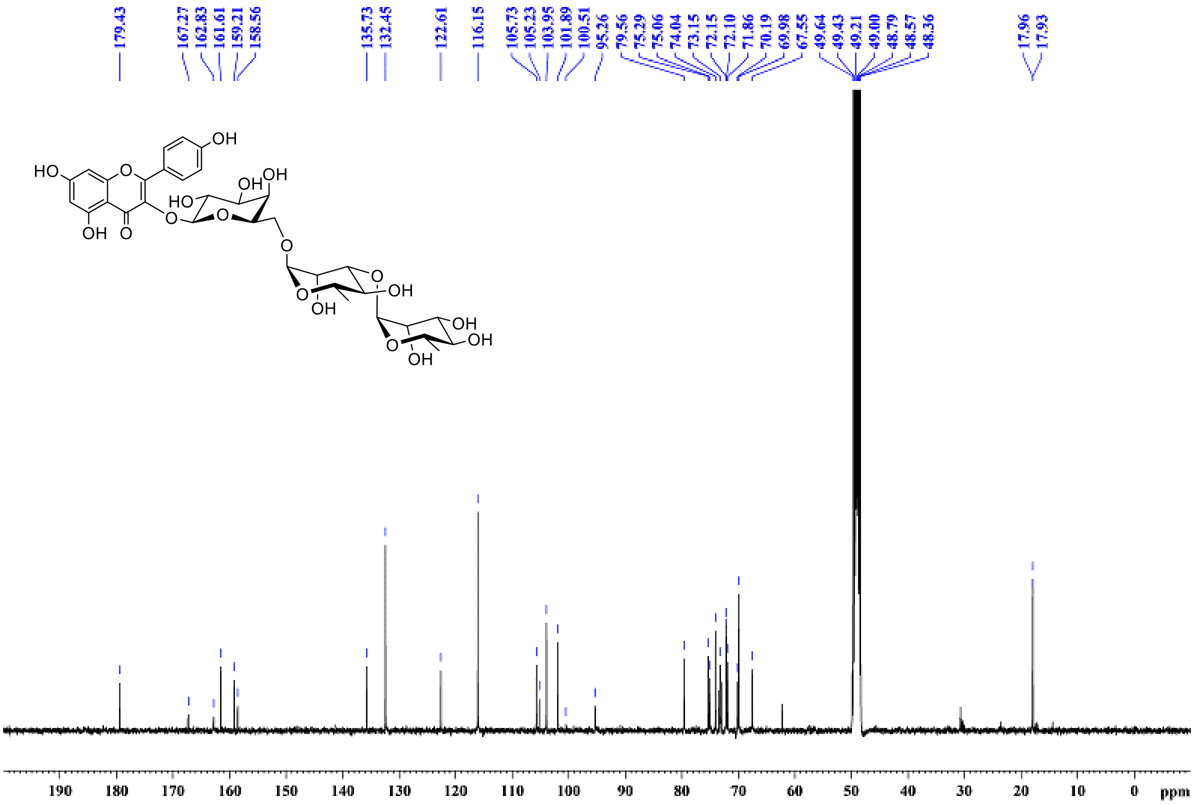

Figure S21. ESI-HRMS spectrum of kaempferol 3-rhamninoside (**10**) in a negative ionization mode

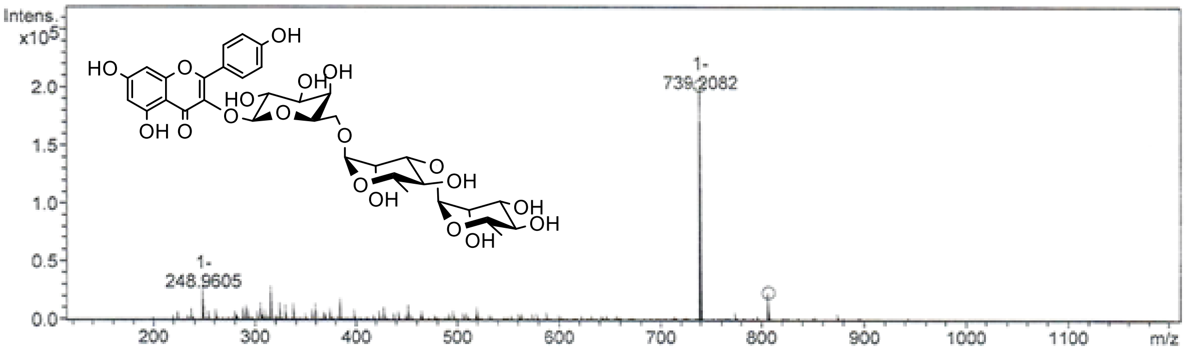

Kaempferol 3-rhamninoside (**10**) had the observed precursor ion at *m*/*z* 739.2082 [M-H]$^-$, calcd for [$C_{33}H_{40}O_{19}$ - H]$^-$, 739.2086, $\Delta_{m/z}$ = 0.54 ppm, and thus having the molecular formula of $C_{33}H_{40}O_{19}$.

Figure S22. ^{1}H NMR (400 MHz) spectrum of quercetin 3-rhamninoside (**11**) in methanol-*d*$_4$

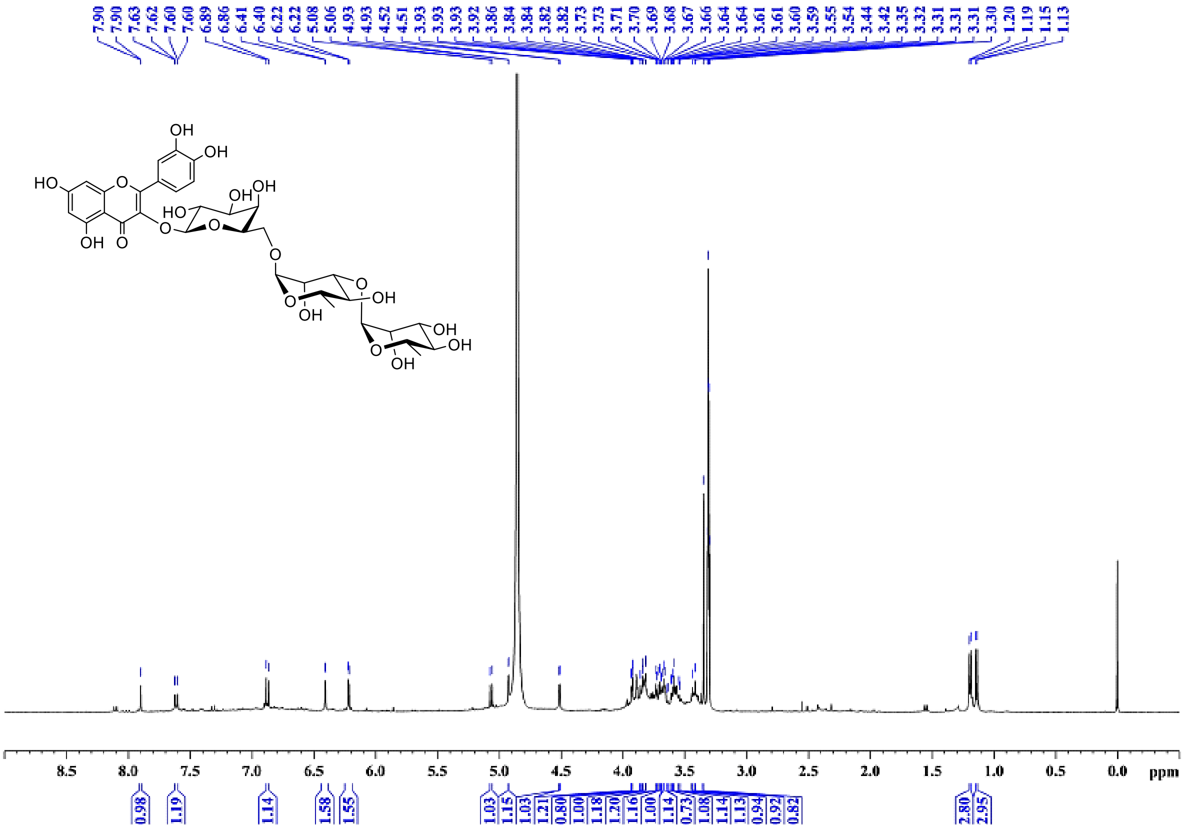

Figure S23. ^{13}C NMR (100 MHz) spectrum of quercetin 3-rhamninoside (**11**) in methanol-*d4*

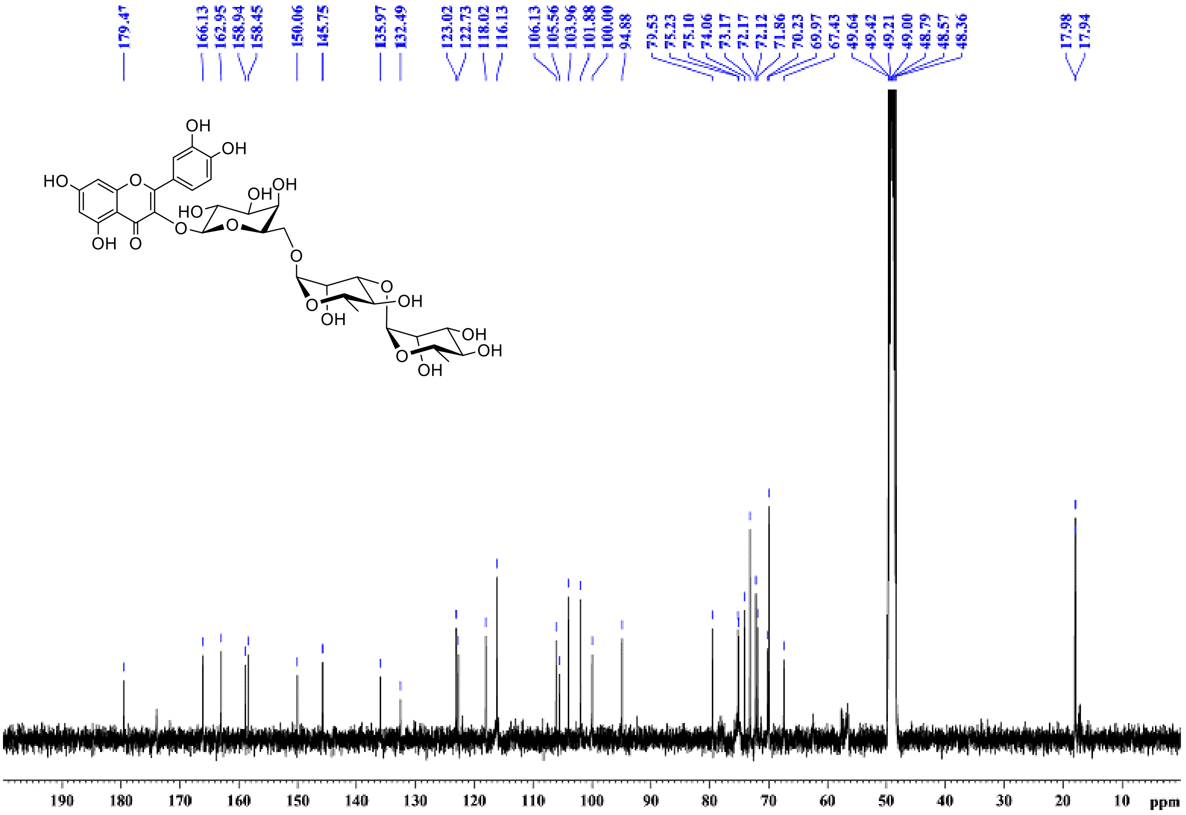

Figure S24. ESI-HRMS spectrum of quercetin 3-rhamninoside (**11**) in a negative ionization mode

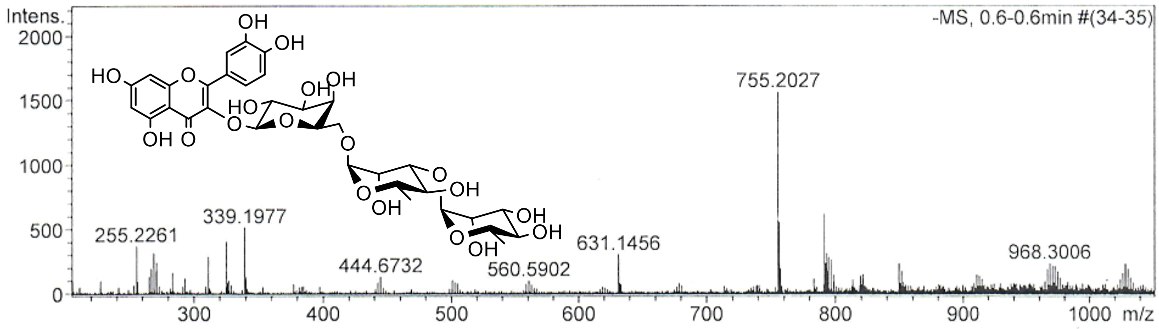

Quercetin 3-rhamninoside (**11**) had the observed precursor ion at *m/z* 755.2027 [M-H]$^-$, calcd for [$C_{33}H_{40}O_{20}$ - H]$^-$, 755.2035, $\Delta_{m/z}$ = 1.06 ppm, and thus having the molecular formula of $C_{33}H_{40}O_{20}$.

Figure S25. MS/MS spectrum of ventilatone B (**12**) parent ion at *m/z* 329.0659 [M+H]$^+$

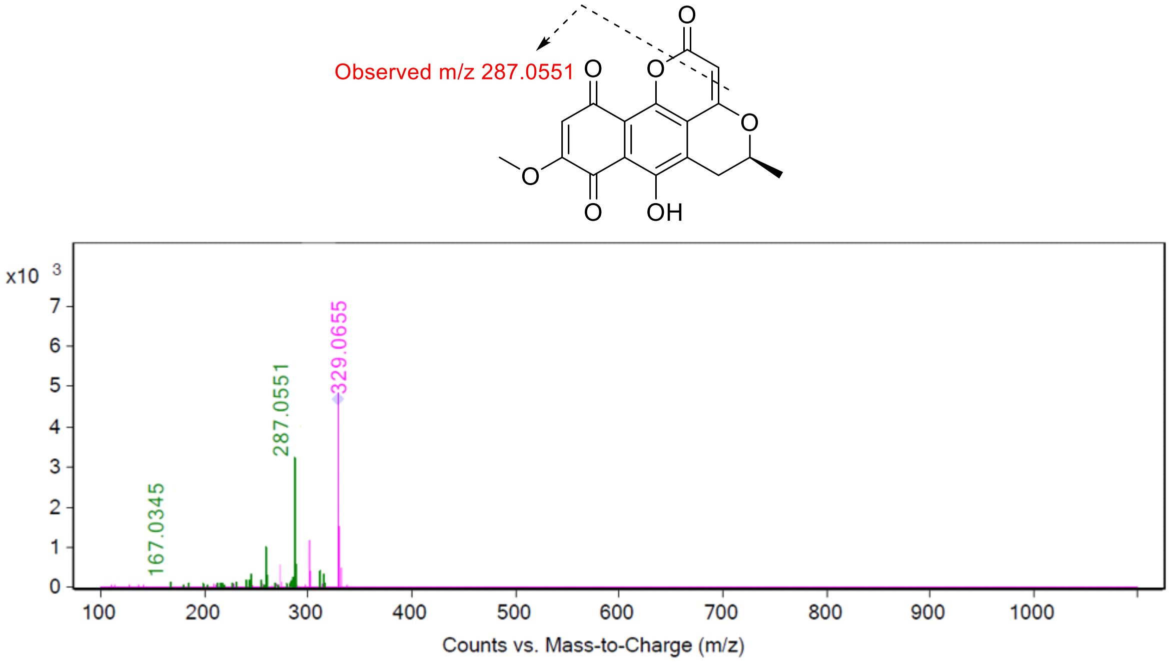

m/z	Calc *m/z*	Diff(ppm)	z	Abund	Formula	Ion
259.0607	259.0601	-2.14	1	1003.07	C14 H11 O5	M+
273.0753			1	580.2		
287.0551	287.055	-0.45	1	3259.39	C15 H11 O6	M+
288.0587	288.0584	-1.06	1	552.34	C15 H11 O6	M+
301.0707			1	1172.65		
302.0741			1	399.07		
311.0551	311.055	-0.15	1	386.88	C17 H11 O6	M+
329.0655			1	4854.88		
330.0697			1	1524.62		
331.0806				468.03		

Figure S26. MS/MS spectrum of ventilatone A (**15**) parent ion at *m/z* 313.0706 [M+H]+

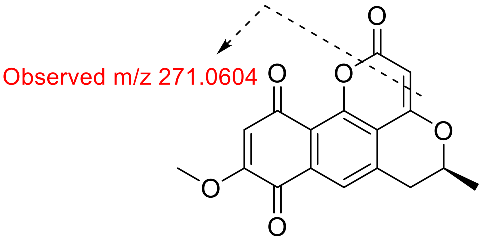

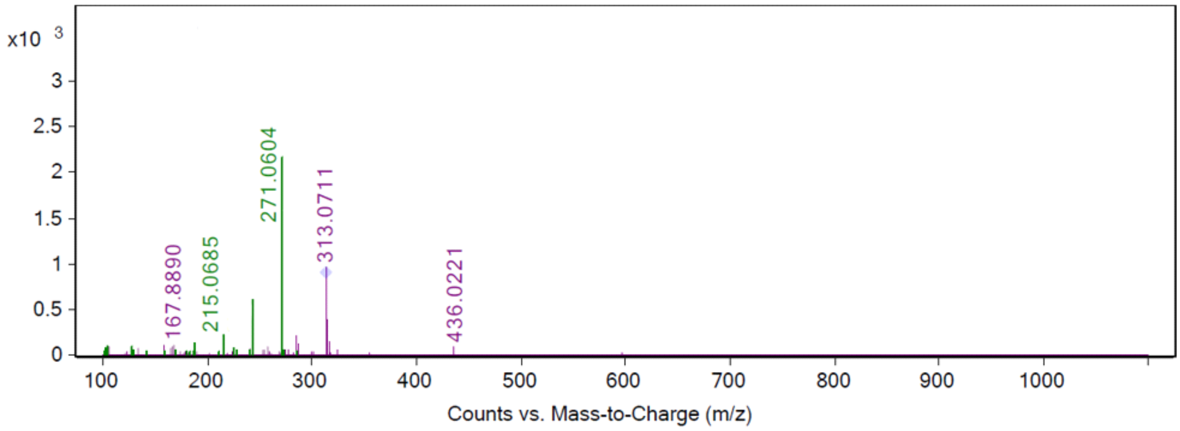

m/z	Calc *m/z*	Diff(ppm)	z	Abund	Formula	Ion
188.0655	188.0679	13.05	1	129.39	C8 H12 O5	M+
215.0685	215.0703	8.05	1	225.83	C13 H11 O3	M+
243.0659	243.0652	-2.9	1	611.12	C14 H11 O4	M+
→ 271.0604	271.0601	-1.11	1	2225.66	C15 H11 O5	M+
272.0616	272.0635	7	1	528.79	C15 H11 O5	M+
285.0759				217.36		
313.0711			1	970.16		
314.074			1	397		
315.0493				140.02		
316.0547			1	144		

Figure S27. ^{1}H-NMR (400 MHz) spectrum of ventilatone B (**12**) in $CDCl_3$

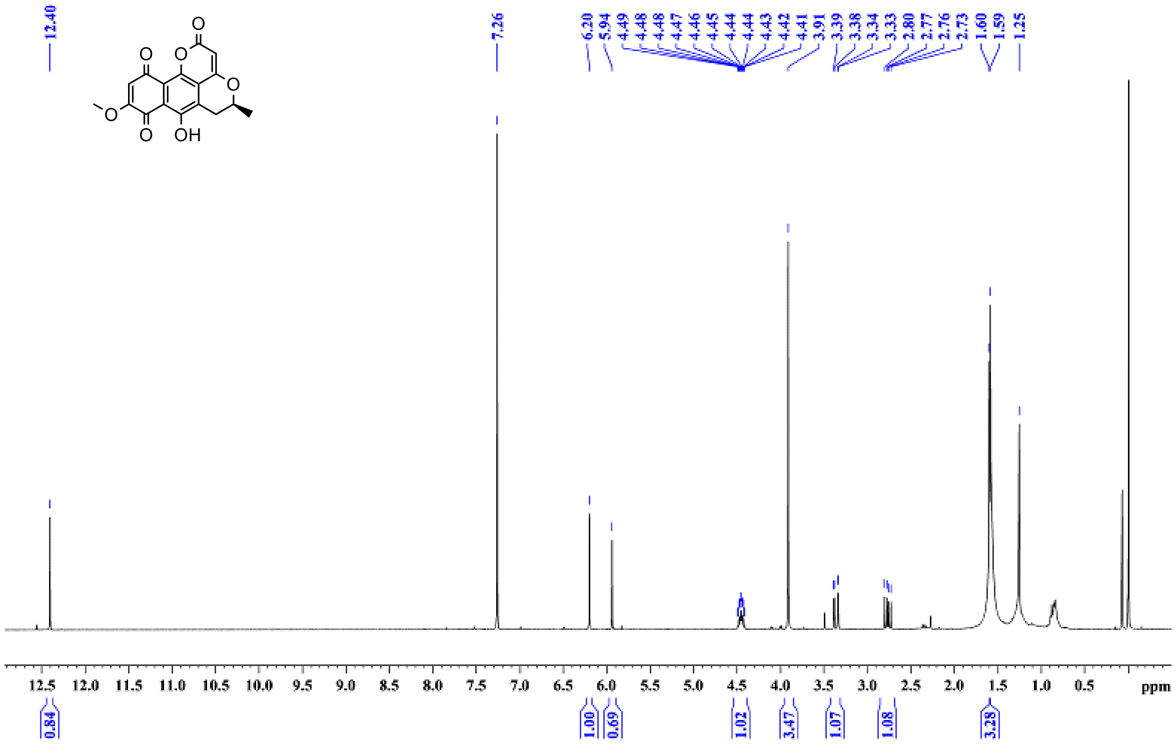

Figure S28. ^{13}C-NMR (100 MHz) spectrum of ventilatone B (**12**) in $CDCl_3$

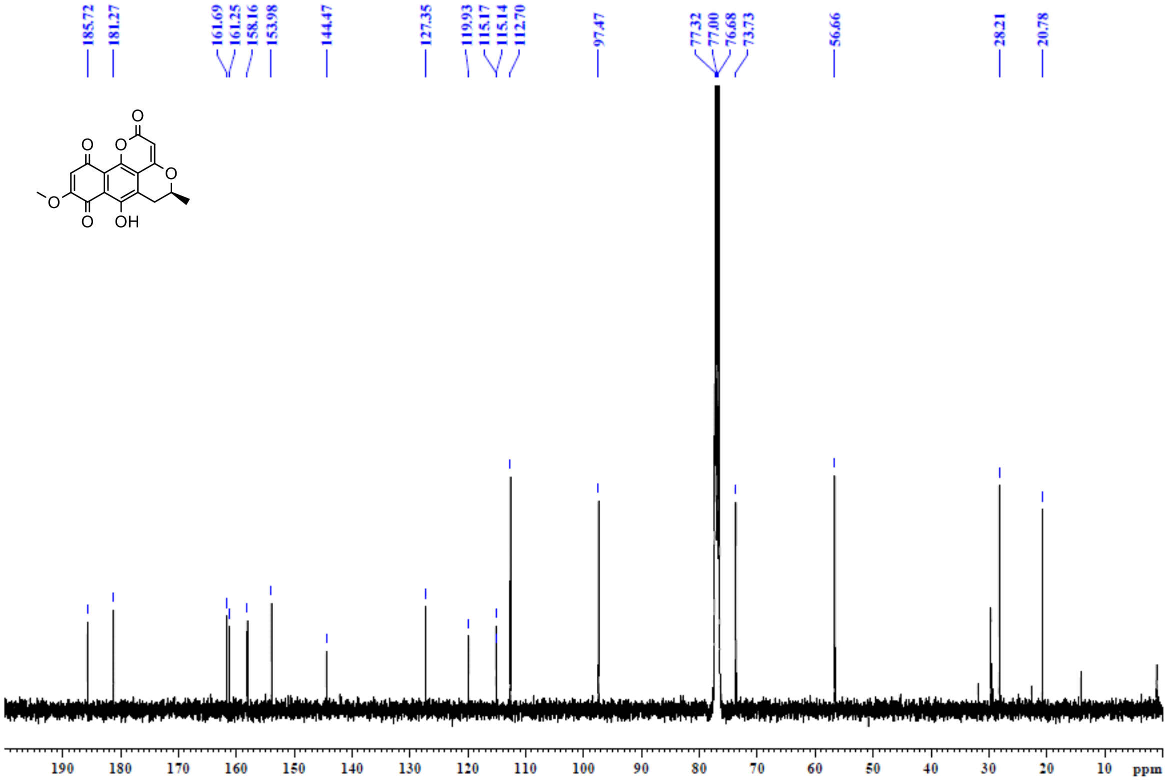

Figure S29. ESI-HRMS spectrum of ventilatone B (**12**) in a negative ionization mode

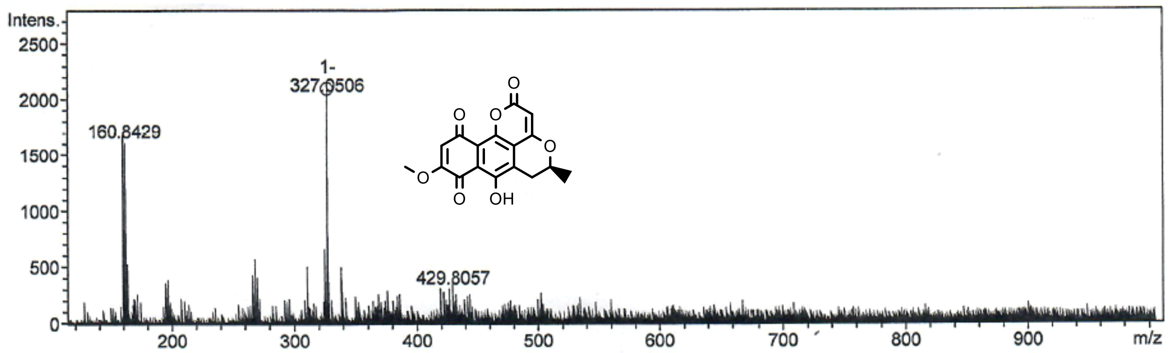

Ventilatone B (**12**) had the observed precursor ion at *m/z* 327.0506 [M-H]⁻, calcd for [$C_{17}H_{12}O_7$ - H]⁻, 327.0505, $\Delta_{m/z}$ = 0.31 ppm, and thus having the molecular formula of $C_{17}H_{12}O_7$.

Figure S30. ^{1}H NMR (400 MHz) spectrum of lupeol (**13**) in $CDCl_3$

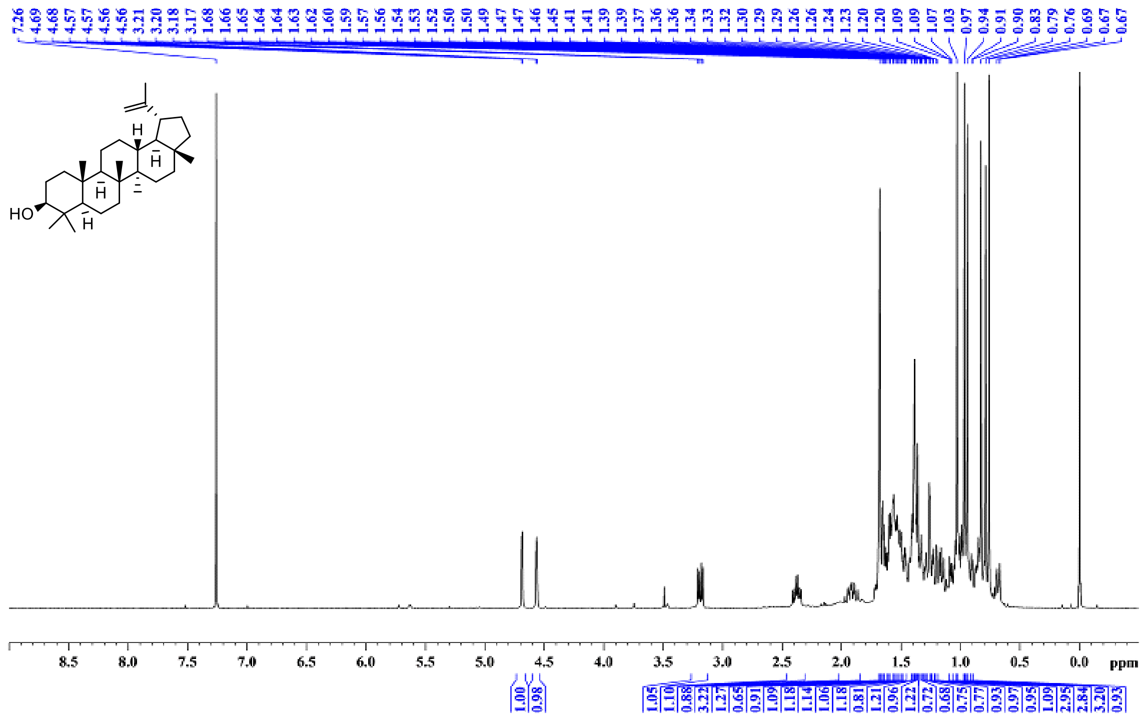

Figure S31. ^{13}C NMR (100 MHz) spectrum of lupeol (**13**) in $CDCl_3$

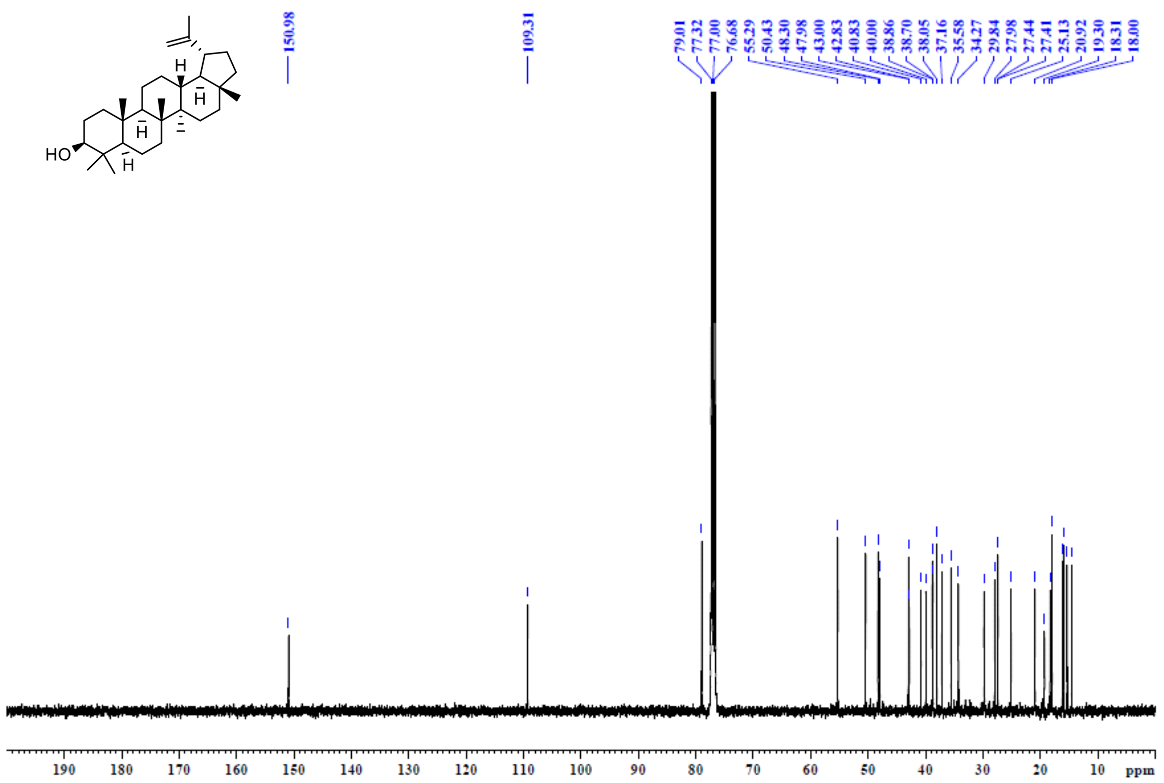

Figure S32. ^{1}H NMR (400 MHz) spectrum of ventilatone A (**15**) in $CDCl_3$

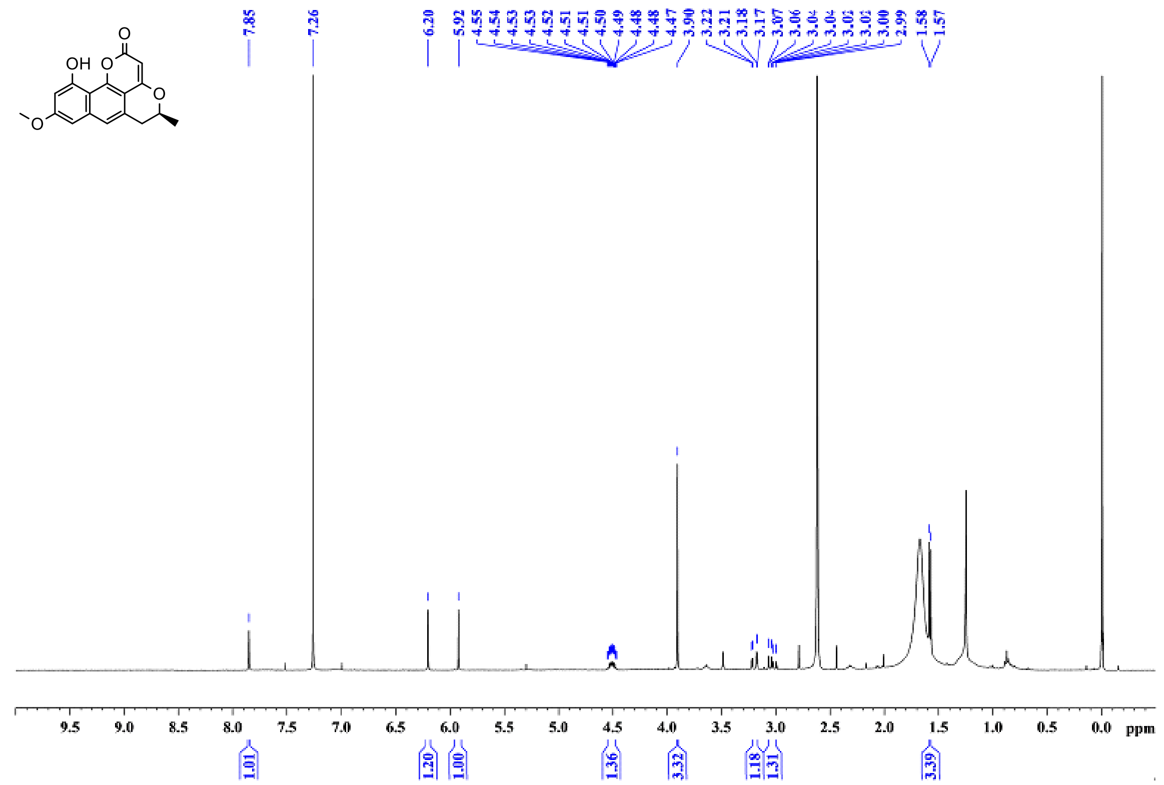

Figure S33. ^{13}C NMR (100 MHz) spectrum of ventilatone A (**15**) in $CDCl_3$

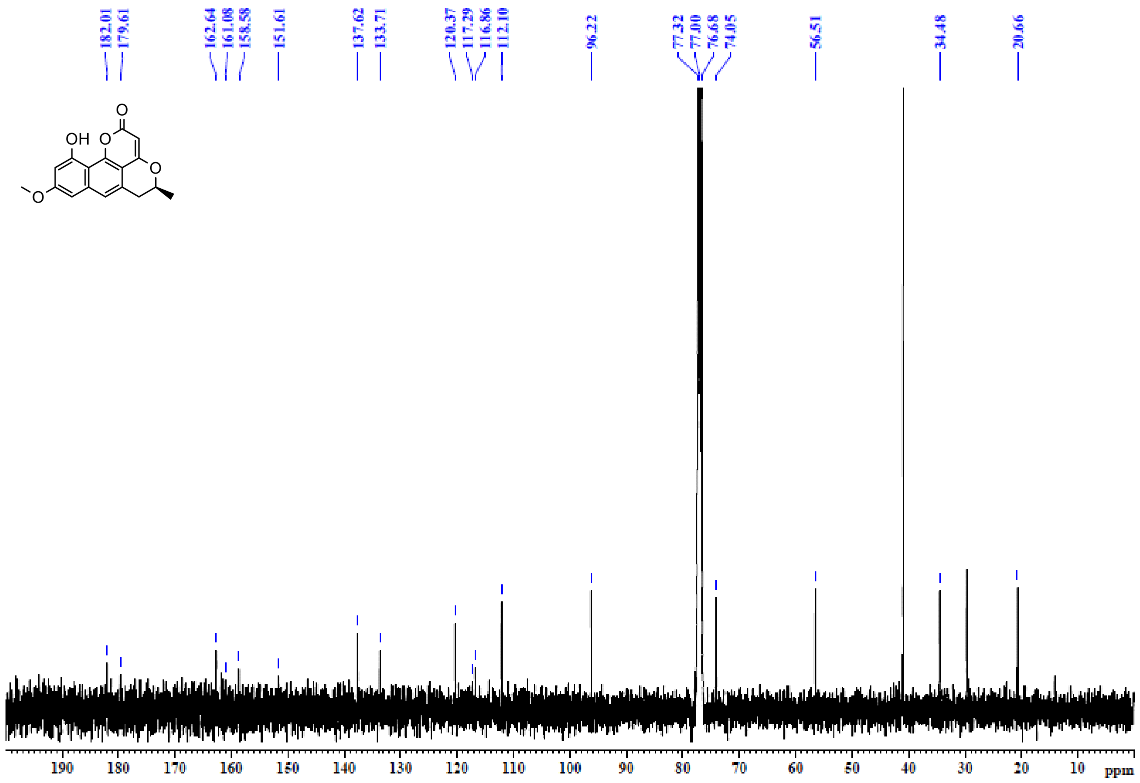

Figure S34. ESI-HRMS spectrum of ventilatone A (**15**) in a negative ionization mode

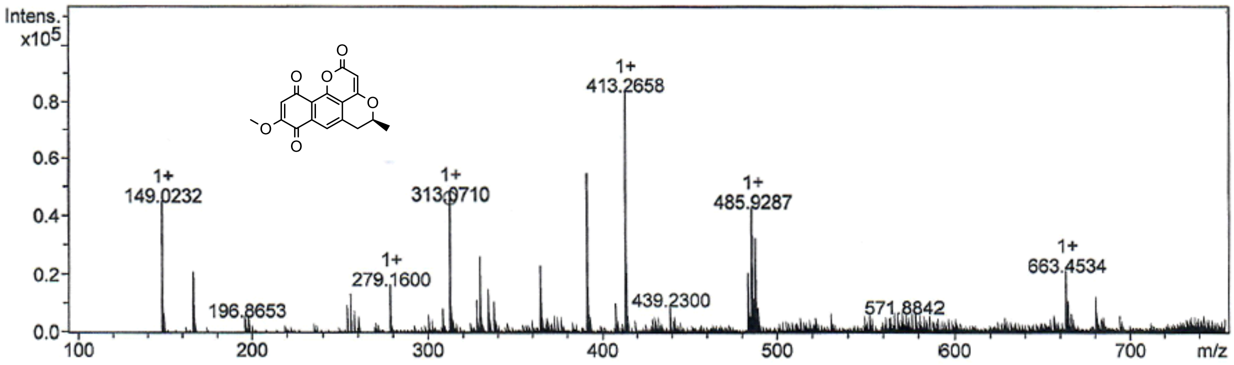

Ventilatone A (**15**) had the observed precursor ion at *m*/*z* 313.0710 $[M+H]^+$, calcd for $[C_{17}H_{12}O_6 + H]^+$, 313.0712, $\Delta_{m/z}$ = 0.64 ppm, and thus having the molecular formula of $C_{17}H_{12}O_6$.

Figure S35. ^{1}H NMR (400 MHz) spectrum of ventilatone C (**16**) in $CDCl_3$

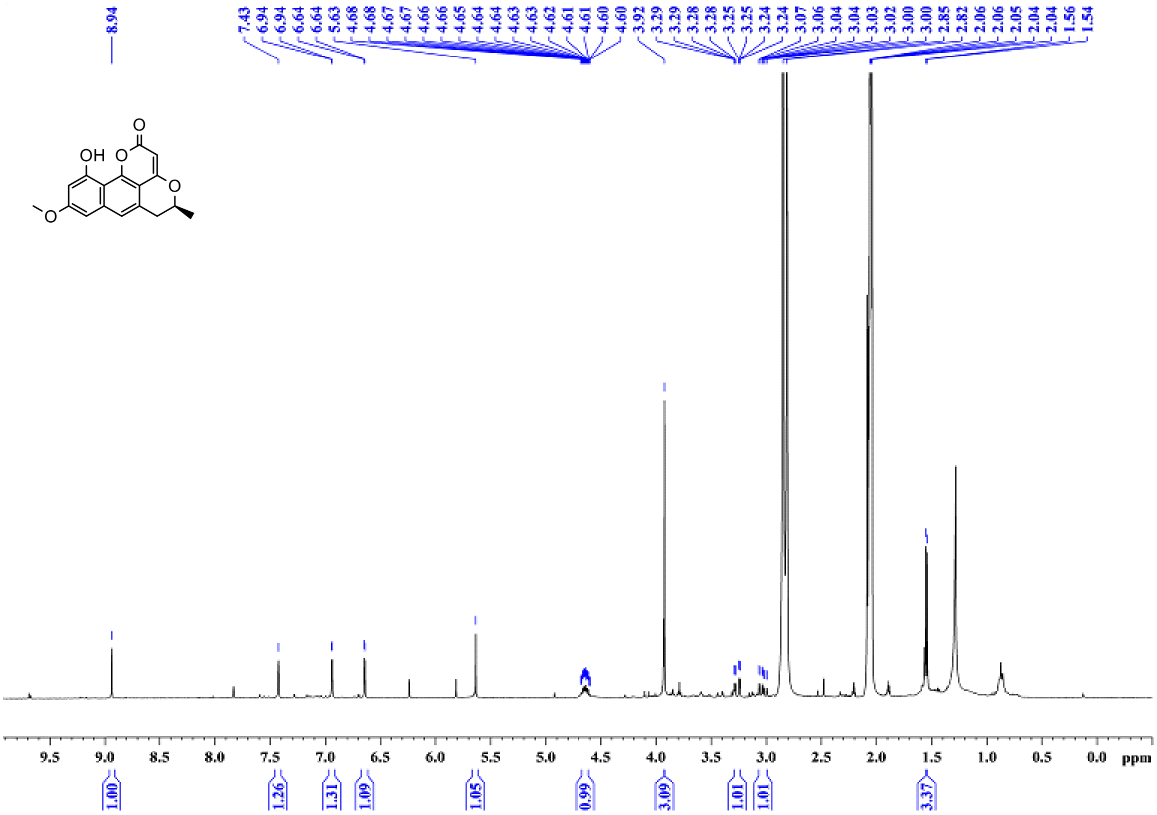

Figure S36. ^{13}C NMR (100 MHz) spectrum of ventilatone C (**16**) in $CDCl_3$

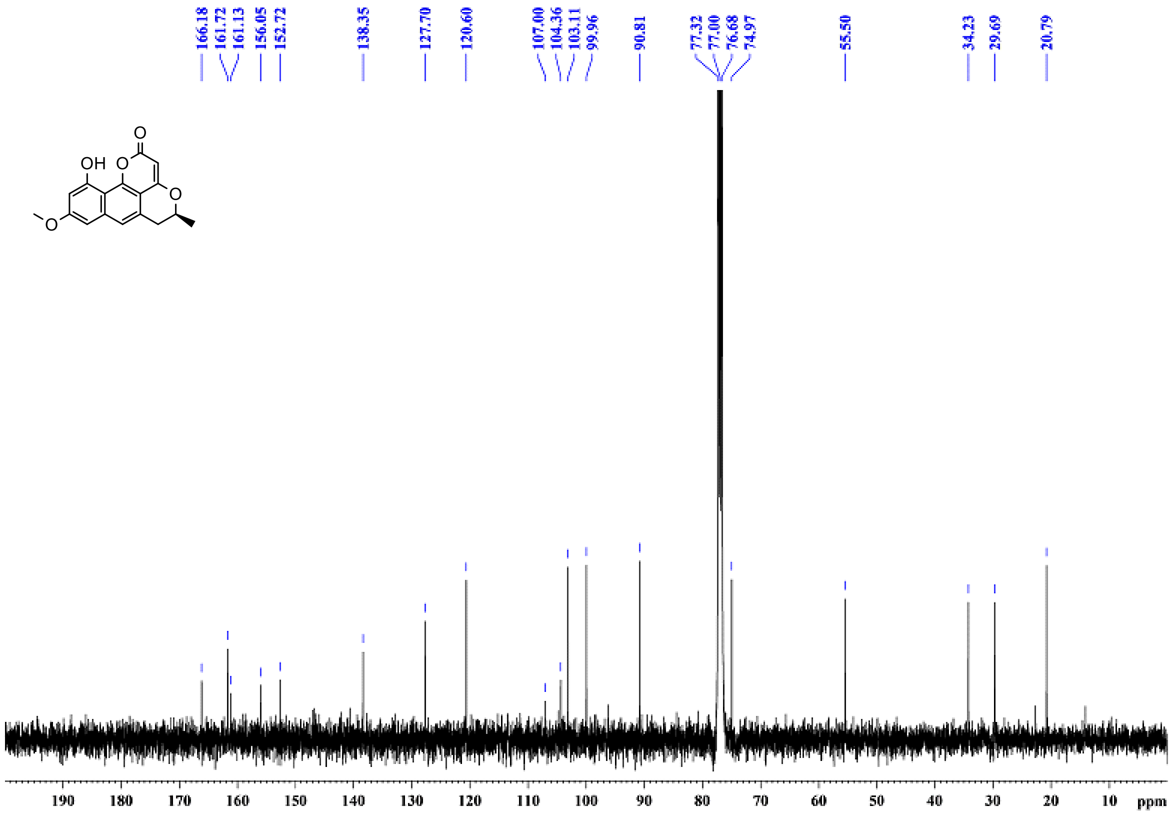

Figure S37. ^{1}H-^{1}H COSY spectrum of ventilatone C (**16**) in $CDCl_3$

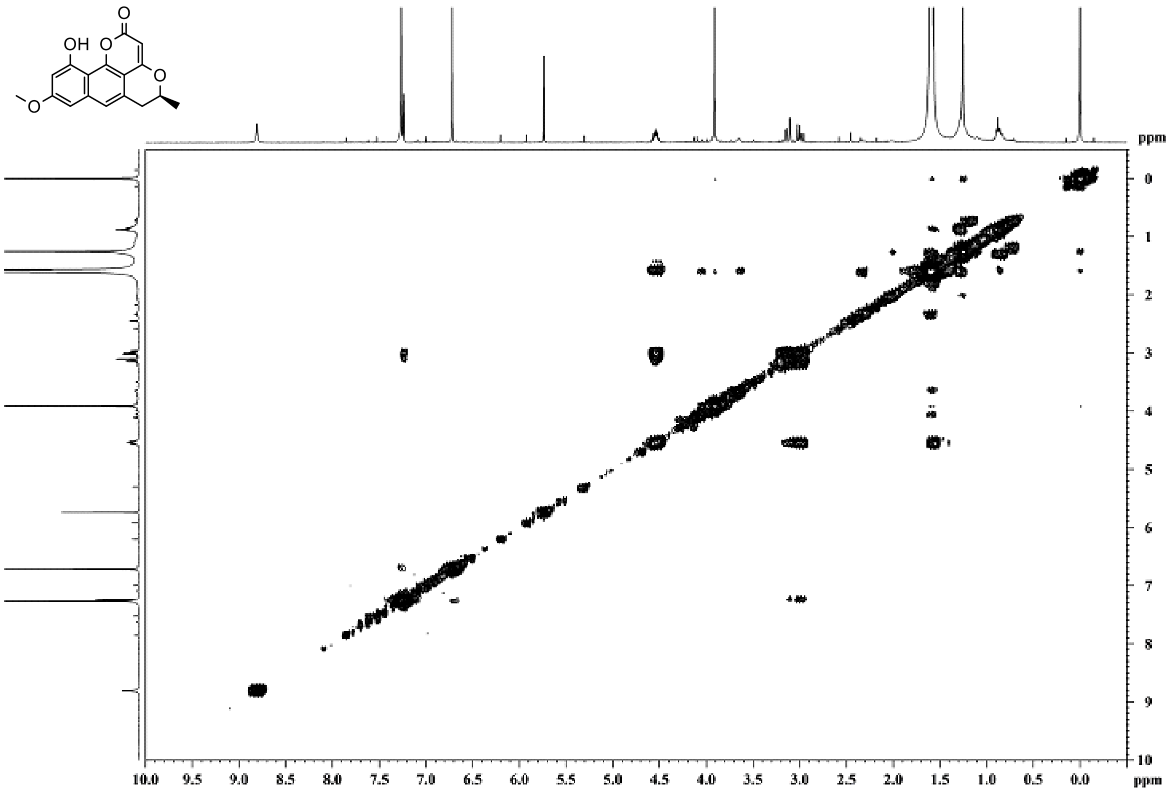

Figure S38. HSQC spectrum of ventilatone C (**16**) in $CDCl_3$

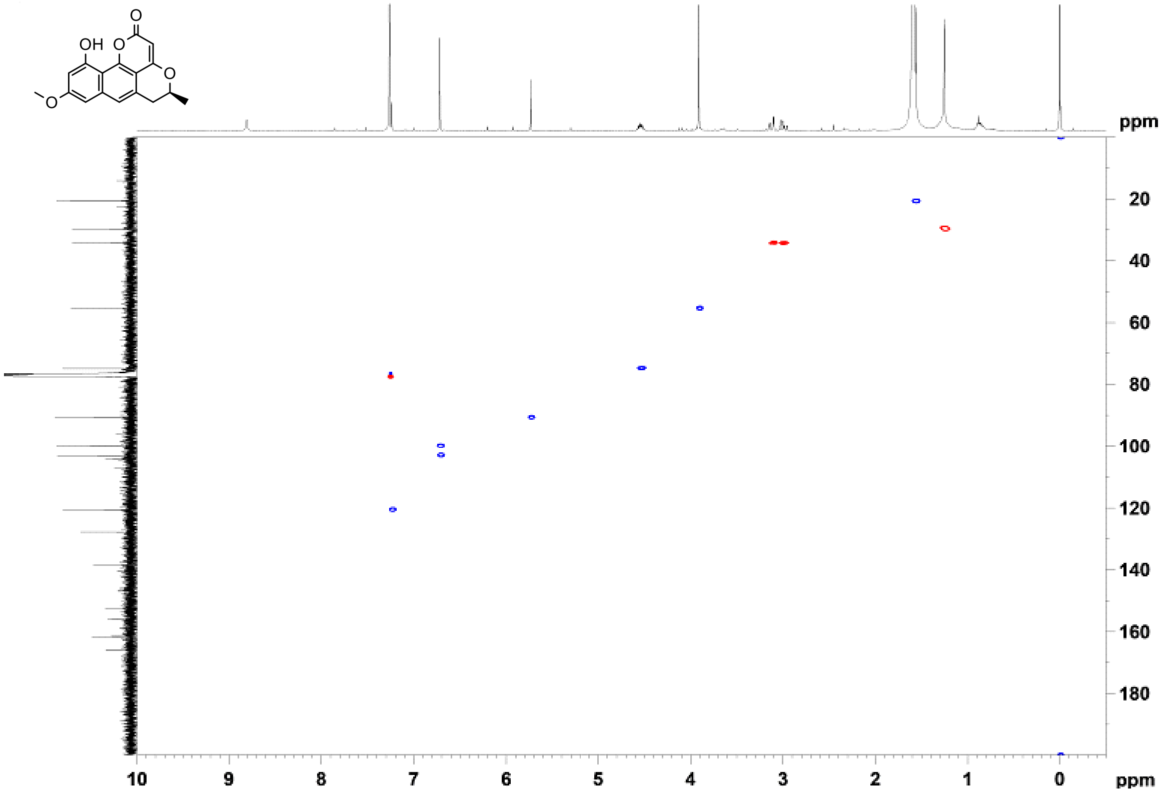

Figure S39. HMBC spectrum of ventilatone C (**16**) in $CDCl_3$

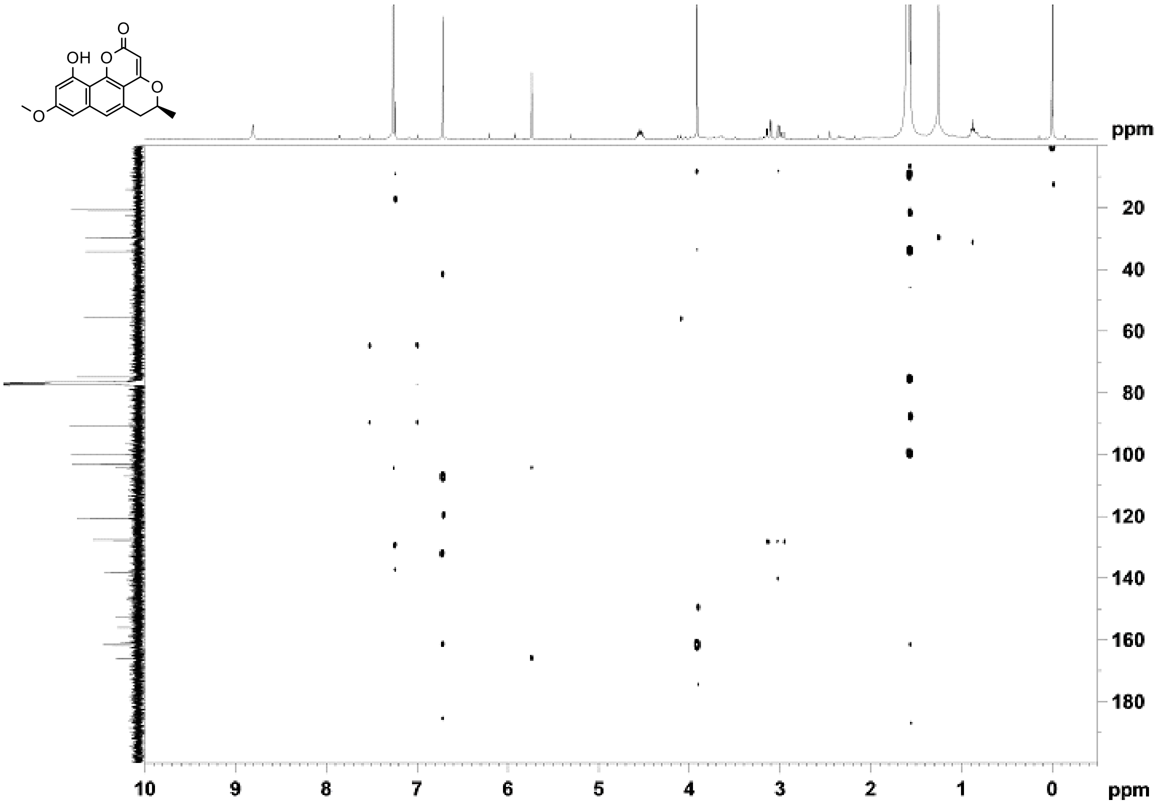

Figure S40. ^{1}H NMR (400 MHz) spectrum of ventilatone C (**16**) in acetone-d_6

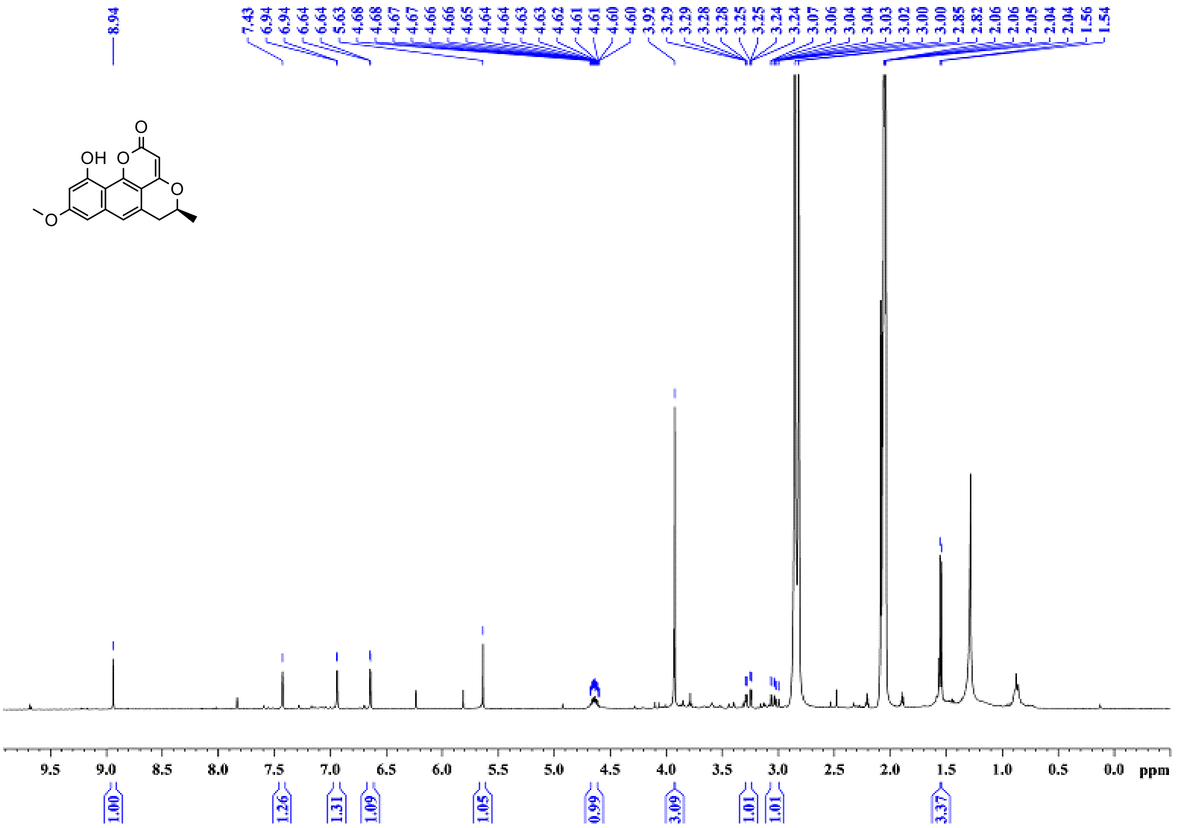

Figure S41. ^{13}C NMR (100 MHz) spectrum of ventilatone C (**16**) in acetone-d_6

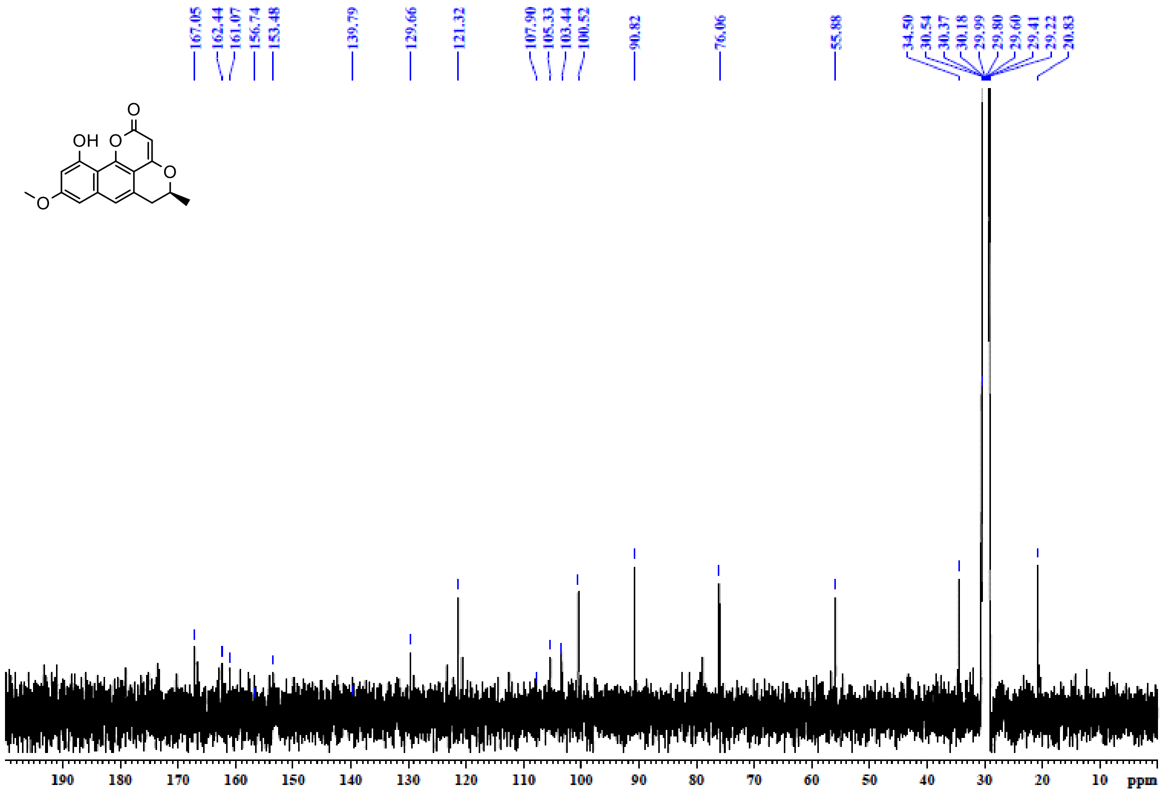

Figure S42. ^{1}H-^{1}H COSY spectrum of ventilatone C (**16**) in acetone-d_6

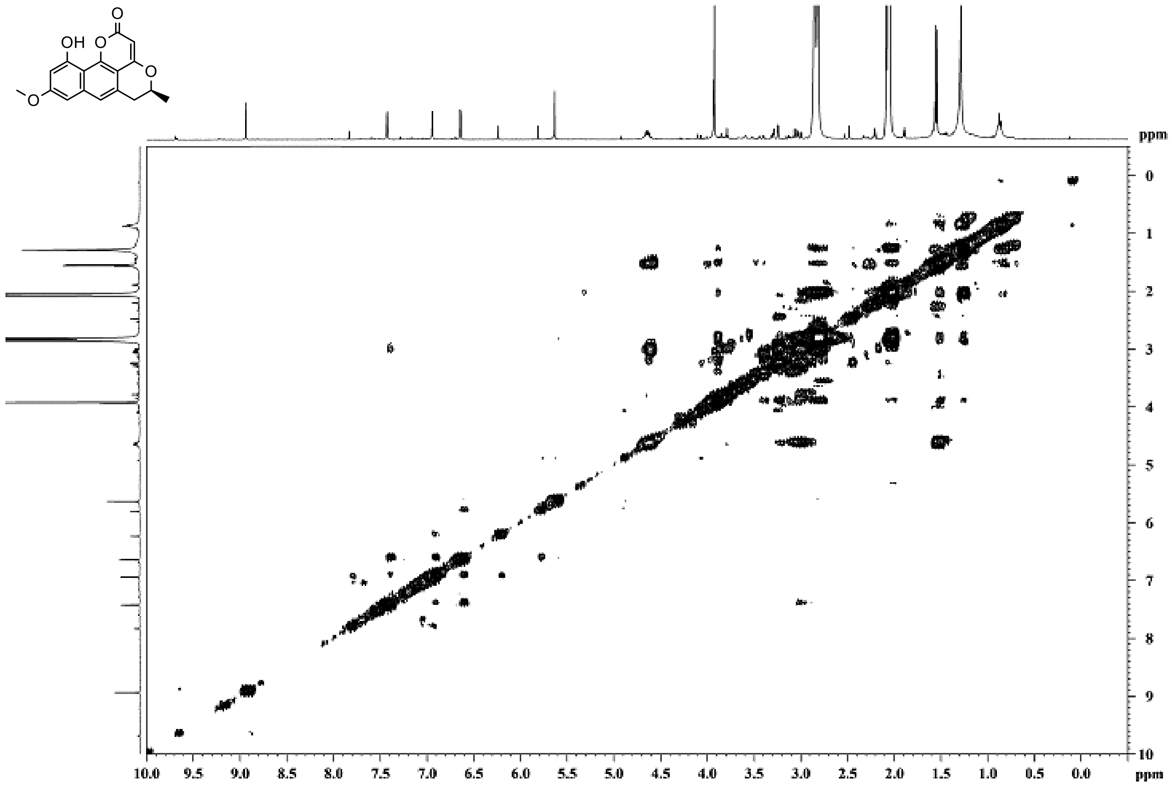

Figure S43. HSQC spectrum of ventilatone C (**16**) in acetone-*d*6

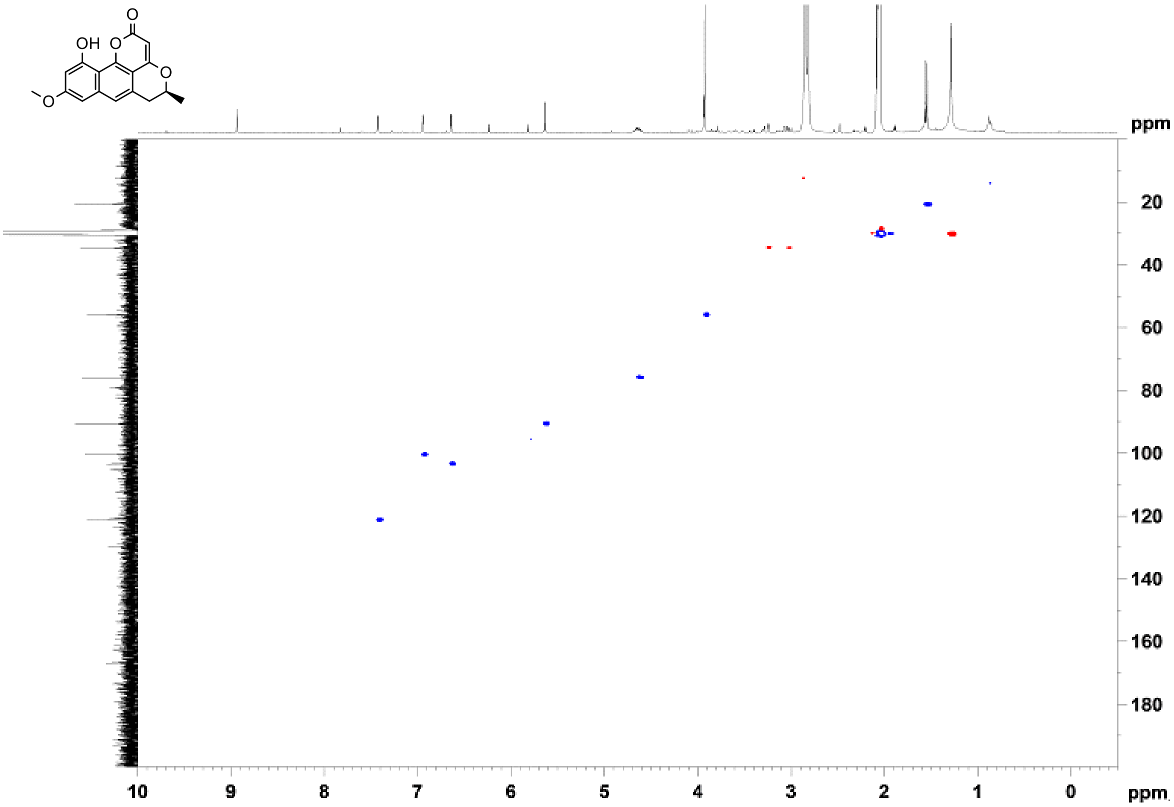

Figure S44. HMBC spectrum of ventilatone C (**16**) in acetone-*d*6

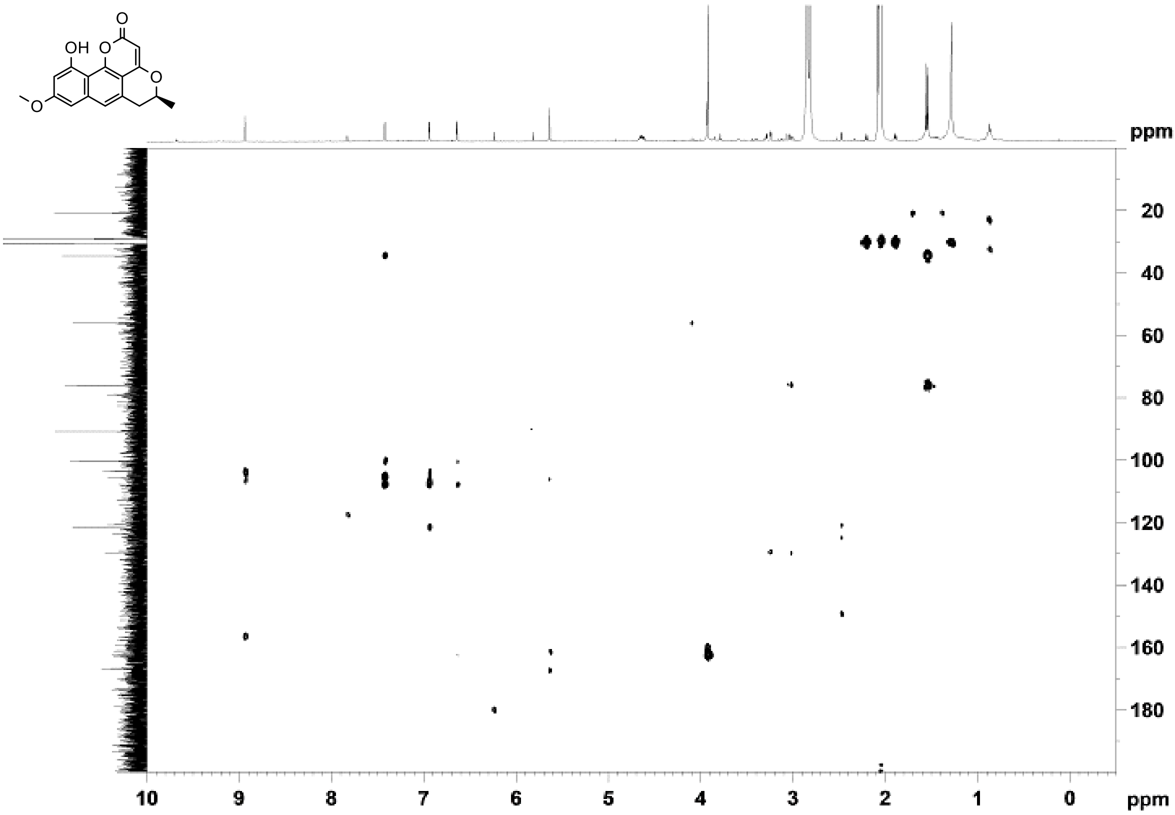

Figure S45. ESI-HRMS spectrum of ventilatone C (**16**) in a positive ionization mode

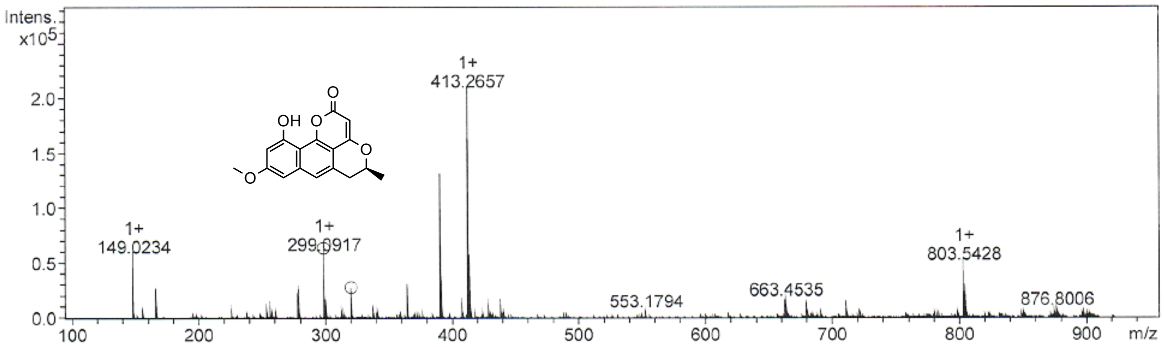

Ventilatone C (**16**) had the observed precursor ion at *m*/*z* 299.0917 $(M+H)^+$, calcd for $[C_{17}H_{14}O_5 + H]^+$, 299.0919, $\Delta_{m/z}$ = 0.67 ppm, and thus having the molecular formula of $C_{17}H_{14}O_5$.

Figure S46. UV spectrum of ventilatone C (**16**) in H_2O:CH_3CN (30:70). This UV spectrum was from a photodiode array detector of HPLC.

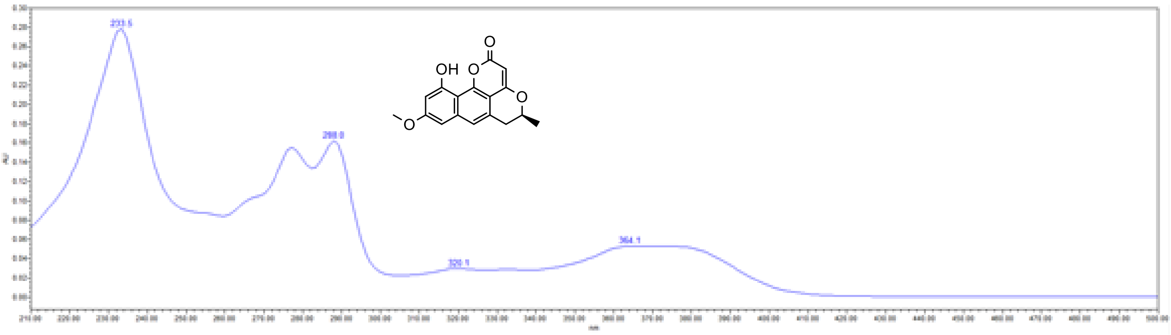

Supplement: Supplementary file 1 [file antibiotics-09-00606-s001.pdf]
